# Infection prevention and control in Indonesian hospitals: identification of strengths, gaps, and challenges

**DOI:** 10.1186/s13756-023-01211-5

**Published:** 2023-02-03

**Authors:** Indri Rooslamiati Supriadi, Cynthia P. Haanappel, Leli Saptawati, Nani H. Widodo, Gortap Sitohang, Yuslely Usman, Ida Bagus Anom, Ratih Dian Saraswati, Michal Heger, Pieter A. Doevendans, Hindra Irawan Satari, Anne F. Voor in ‘t holt, Juliëtte A. Severin

**Affiliations:** 1 Center for Health Policy on Resilience System and Resource, Health Policy Agency, Ministry of Health of Indonesia, Percetakan Negara 23, Jakarta, Indonesia; 2 Department of Cardiology, University Medical Centre Utrecht, Utrecht, The Netherlands; 3 Department of Medical Microbiology and Infectious Diseases, Erasmus MC University Medical Centre, Rotterdam, The Netherlands; 4 Department of Microbiology, Faculty of Medicine, Universitas Sebelas Maret, Surakarta, Indonesia; 5 Department of Microbiology, Moewardi Teaching Hospital, Surakarta, Indonesia; 6 Directorate of Referral Health Care, Ministry of Health of Indonesia, Jakarta, Indonesia; 7 Infection Prevention and Control Committee, Dr. Cipto Mangunkusumo General Hospital, Jakarta, Indonesia; 8 Center for Health Financing and Decentralization Policy, Health Policy Agency, Ministry of Health of Indonesia, Jakarta, Indonesia; 9 Department of Pharmaceutics, Utrecht Institute for Pharmaceutical Sciences, Utrecht University, Utrecht, The Netherlands; 10 Membrane Biochemistry and Biophysics, Department of Chemistry, Faculty of Science, Utrecht University, Utrecht, The Netherlands; 11 Laboratory for Experimental Oncology, Department of Pathology, Erasmus MC University Medical Centre, Rotterdam, The Netherlands; 12 Jiaxing Key Laboratory for Photonanomedicine and Experimental Therapeutics, Department of Pharmaceutics, College of Medicine, Jiaxing University, Jiaxing, Zhejiang China; 13 Department of Child Health, Faculty of Medicine, Universitas Indonesia, Jakarta, Indonesia

**Keywords:** Healthcare-associated infections, Microbiology, World Health Organization, Patient safety, Survey

## Abstract

**Background:**

Infection prevention and control (IPC) in hospitals is key to safe patient care. There is currently no data regarding the implementation of IPC in hospitals in Indonesia. The aim of this study was to assess the existing IPC level in a nationwide survey, using the World Health Organization (WHO) IPC assessment framework tool (IPCAF), and to identify strengths, gaps, and challenges.

**Methods:**

A cross-sectional study was conducted from July to November 2021. Of all general hospitals in Indonesia, 20% (N = 475) were selected using stratified random sampling based on class (A, B, C and D; class D with a maximum of 50 beds and class A with ≥ 250 beds) and region. The IPCAF was translated into Indonesian and tested in four hospitals. Questions were added regarding challenges in the implementation of IPC. Quantitative IPCAF scores are reported as median (minimum–maximum). IPC levels were calculated according to WHO tools.

**Results:**

In total, 355 hospitals (74.7%) participated in this study. The overall median IPCAF score was 620.0 (535.0–687.5). The level of IPC was mostly assessed as advanced (56.9% of hospitals), followed by intermediate (35.8%), basic (7.0%) and inadequate (0.3%). In the eastern region of the country, the majority of hospitals scored intermediate level. Of the eight core components, the one with the highest score was IPC guidelines. Almost all hospitals had guidelines on the most important topics, including hand hygiene. Core components with the lowest score were surveillance of healthcare-associated infections (HAIs), education and training, and multimodal strategies. Although > 90% of hospitals indicated that surveillance of HAIs was performed, 57.2% reported no availability of adequate microbiology laboratory capacity to support HAIs surveillance. The most frequently reported challenges in the implementation of IPC were communication with the management of the hospitals, followed by the unavailability of antimicrobial susceptibility testing results and insufficient staffing of full-time IPC nurses.

**Conclusion:**

The IPC level in the majority of Indonesian hospitals was assessed as advanced, but there was no even distribution over the country. The IPCAF in combination with interviews identified several priority areas for interventions to improve IPC in Indonesian hospitals.

**Supplementary Information:**

The online version contains supplementary material available at 10.1186/s13756-023-01211-5.

## Background

The World Health Organization (WHO) recently estimated that 15% of patients in low- and middle-income countries (LMICs) will acquire at least one healthcare-associated infection (HAI) during their hospital stay [1]. Infection prevention and control (IPC) is key to reduce these infections, including those caused by antimicrobial resistant (AMR) bacteria [2].

The level of implementation of IPC programs and practices can be measured by tools provided by the WHO, such as the Infection Prevention and Control Assessment Framework (IPCAF), which is based on previously defined eight core components (CCs) of IPC (Additional file 1). The IPCAF is a structured questionnaire-like tool with 81 indicators and a scoring system [3]. Based on the total score of the eight CCs, a healthcare facility can be assigned to one of four levels of implementation: inadequate (score of 0–200), basic (score of 201–400), intermediate (score of 401–600) and advanced (score of 601–800). In a recent global study, 4440 healthcare facilities in 81 countries participated in a survey using this IPCAF tool to assess IPC programs [4]. The data revealed that implementation of IPC programs was significantly inferior in low-income countries and lower-middle-income countries compared to high-income countries. However, data of only six low-income countries and 13 lower-middle-income countries were included, and only four of 11 Southeast Asian countries. Large economically upcoming countries such as Indonesia were not included in the analysis.

Indonesia is a lower-middle-income country in South East Asia and the fourth most populous country in the world [5]. The Ministry of Health of Indonesia released a national guideline for IPC implementation in 2017 [6]. However, an assessment of the degree of IPC implementation has not been performed hitherto. Baseline data is needed for hospitals as well as for policymakers to formulate strategies and interventions to improve IPC management in Indonesia, if necessary. The aim of the study was therefore to assess the existing IPC practices in a nationwide survey, using the IPCAF tool and additional interviews, and to identify strengths, gaps, and challenges to Indonesian IPC policies.

## Methods

### Study design and selection of hospitals

A cross-sectional study was conducted from July to November 2021. Of all general hospitals in Indonesia, 20% (N = 475) were selected using stratified random sampling on the basis of class and region. Hospitals in Indonesia are classified into four classes (A, B, C and D) based on the number of beds and services (Additional file 2) [7]. There were 2,373 general hospitals in Indonesia (per February 2021): class A, 24 hospitals (1.0%), class B, 376 hospitals (15.8%), class C, 1146 hospitals (48.3%) and class D, 827 hospitals (34.9%) [8]. Since Indonesia is a large country, regions were taken into consideration to ensure all regions were well represented. The regions, as defined by the National Development Agency, were used [9]: region 1 (Sumatra, Java, and Bali), region 2 (Kalimantan, Sulawesi, and West Nusa Tenggara), and region 3 (East Nusa Tenggara, Papua, West Papua, and Maluku). Of the participating hospitals, 10% were selected for two additional semi-structured interviews; one with the management and one with the IPC team or committee. For the selection, stratified random sampling by class and region of the hospitals was applied as well. The interviews were conducted after the IPCAF assessment was completed. The study was approved by the Health Research Ethical Board of National Health Research and Development (No: LB.02.01/2/KE.494/2021).

### Questionnaires

The WHO IPCAF questionnaire was translated into Indonesian by the first author and an independent researcher (both Indonesian native speakers) and adapted to the situation in hospitals in Indonesia (Additional file 3). The most important adaptation was the ratio of IPC nurse (IPCN) per number of beds. In the WHO IPCAF, this is set at a minimum of 1 IPCN per 250 beds, while the Indonesian government has mandated a minimum of 1 IPCN per 100 beds. Several discussions were held to review the questions with local IPC experts from the National Committee of IPC, the Ministry of Health, and the WHO.

Several questions were added regarding the characteristics of the hospitals, challenges, and recommendations for the improvement of IPC implementation on a hospital and national level, but these were kept separate from the IPCAF questions in such a way that the scoring as per the original IPCAF tool could be performed (Additional file 4). For the implementation challenge questions, 11 challenges were given in the questionnaire, and each hospital was asked to rank these challenges in order from high to low priority based on the situation in their hospital. A low number corresponded with a high priority. Subsequently, the median rank of each challenge was determined from all hospitals.

The questionnaire used for the semi-structured interviews with managers and IPC committees was translated into Indonesian from the questionnaires previously used in a study from Georgia that described the challenges and opportunities in implementing IPC [10]. Additional open questions about recommendations for implementing IPC in hospitals were asked to the management of the hospital. Translated questionnaires were entered into an online collection tool (Lime Survey). To obtain complete questionnaires, answering all questions was mandatory. Translated questionnaires for the semi-structured interviews can be found in Additional file 5 (interview for management of hospitals) and Additional file 6 (interview for IPC team/committee).

### Pilot testing

A pilot study was conducted to test the online questionnaires and interviews in four hospitals (one of each class) in Java, which were not included in the study sample selection. An online introduction meeting was organized to provide the four hospitals with information needed to properly conduct the pilot study. The steps for filling out the questionnaires were explained and shown in an instructional video that was uploaded on YouTube (https://youtu.be/pyykZCY_H0A). Telephone numbers and emails were provided to the hospitals to contact organizing staff when they encountered any difficulty in filling out the questionnaires. A link to the questionnaire was sent to all hospitals and interviews were conducted with the management and IPC committee of the hospitals. Focus group discussions were held with all four hospitals for the improvement of data collection and questionnaire procedures.

### Data collection and scoring

Data collection was performed by a team consisting of IPC experts who received training about the IPCAF CCs, study protocols, and interview technique. Data were collected from August until November 2021. The steps for collecting data were the same as those carried out in the pilot study. Introduction meetings for the selected hospitals were conducted and informed consent to use the data was also included in the questionnaires and obtained from all participants. Participation was voluntary, the facility data were kept confidential, access was restricted to the research team, and results would not affect the participating hospitals’ accreditation status.

The questionnaires were filled in by members of management, the IPC committee, and other units that were involved in the implementation of IPC. Reminders by WhatsApp, telephone, text messages, and email were sent once a week. Submitted complete questionnaires were checked and, in case of any unclarity, the team contacted the respective hospital. Interviews with management of the hospitals and IPC committees were organized after all questionnaires had been submitted. These interviews were all conducted separately and online. The questions in the interview with the IPC committee focused on the three CCs that had the lowest score in the IPCAF scores of all hospitals taken together. The interviewer also asked for proof of documents (pictures, video documentation, certificates) when applicable during the interviews. The flow of the study is presented in Additional file 7.

Based on the total score of the eight CCs, a healthcare facility can be assigned to one of four levels of implementation: inadequate (score of 0–200), basic (score of 201–400), intermediate (score of 401–600) and advanced (score of 601–800) (Additional file 8).

### Statistical analysis

Data from IPCAF questionnaires were analyzed using SPSS version 28.0 (IBM, Armonk, NY, USA). Hospital characteristics are presented as absolute numbers and proportions. Quantitative IPCAF scores are presented as medians with a range of minimum and maximum. ANOVA, Kruskal–Wallis, Welch, and Mann–Whitney U tests were used where appropriate to test for differences between questionnaire scores and hospital characteristics such as hospital class, region/island, ownership (private/government), accreditation status, and/or presence of a microbiological laboratory. Post-hoc analyses were performed using the Tukey, Mann–Whitney U, and Games-Howell test where appropriate. A *P* value of < 0.05 was considered statistically significant.

## Results

**Fig. 1:**
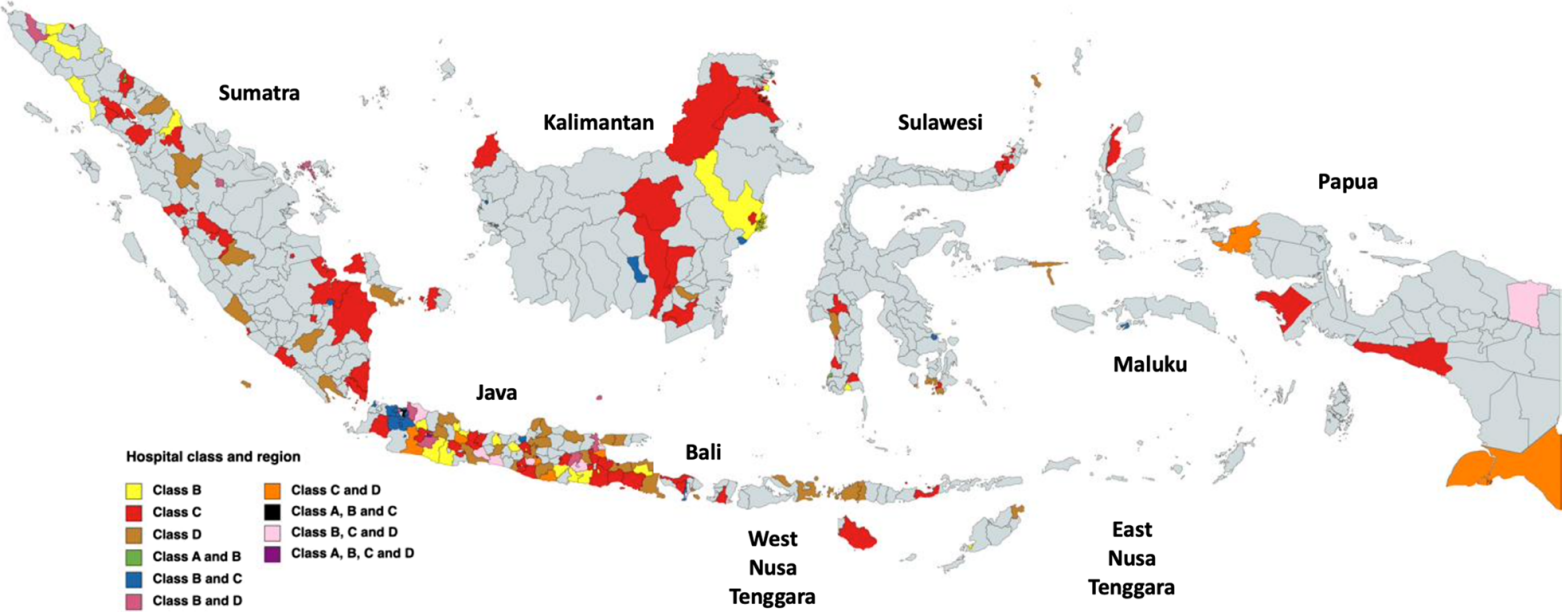
Regional distribution of the 355 participating hospitals

The invitation was sent to 475 hospitals, of which 355 (74.7%) hospitals participated. All classes of hospitals and all regions in Indonesia were represented (Fig. 1). The characteristics of participating hospitals are presented in Table 1. Interviews were conducted with management and the IPC committee of 38 (10%) hospitals.

**Table 1:** Level of implementation of infection prevention and control (IPC) in 355 participating hospitals

	Inadequate (0–200) N (%)	Basic (201–400) N (%)	Intermediate (401–600) N (%)	Advanced (601–800) N (%)	Total N (%)
Class					
A	0 (0.0)	0 (0.0)	1 (12.5)	7 (87.5)	8 (100.0)
B	0 (0.0)	2 (2.0)	19 (19.4)	77 (78.6)	98 (100.0)
C	0 (0.0)	13 (7.9)	67 (40.6)	85 (51.5)	165 (100.0)
D	1 (1.2)	10 (11.9)	40 (47.6)	33 (39.3)	84 (100.0)
Region					
1 (Java, Sumatra, Bali)	0 (0.0)	16 (5.8)	96 (35.0)	162 (59.1)	274 (100.0)
2 (Sulawesi, Kalimantan, West Nusa Tenggara)	1 (2.0)	5 (9.8)	16 (31.4)	29 (56.9)	51 (100.0)
3 (Maluku, Papua, West Papua, East Nusa Tenggara)	0 (0.0)	4 (13.3)	15 (50.0)	11 (36.7)	30 (100.0)
Ownership of the hospital					
Private	0 (0.0)	6 (3.0)	66 (33.3)	126 (63.6)	198 (100.0)
State-owned enterprise	0 (0.0)	0 (0.0)	4 (44.4)	5 (55.6)	9 (100.0)
Government	1 (0.7)	19 (12.8)	57 (38.3)	71 (47.7)	149 (100.0)
Level of national accreditation*					
First pass	0 (0.0)	0 (0.0)	9 (42.9)	12 (57.1)	21 (100.0)
Basic	1 (2.8)	9 (25.0)	16 (44.4)	10 (27.8)	36 (100.0)
Intermediate	0 (0.0)	3 (6.3)	25 (52.1)	20 (41.7)	48 (100.0)
Main	0 (0.0)	6 (11.8)	23 (45.1)	22 (43.1)	51 (100.0)
Plenary	0 (0.0)	2 (1.1)	45 (25.3)	130 (73.0)	178 (100.0)

*The highest level of national accreditation is plenary, and the lowest level of national accreditation is first pass

### Overall IPCAF scores

The overall median (range) IPCAF score of all participating hospitals was 620.0 (535.0–687.5) (Additional file 9), which corresponds to an advanced IPC implementation level. With respect to the total IPCAF score, 202 hospitals (56.9%) had an advanced level of IPC implementation, 127 hospitals (35.8%) had an intermediate level, 25 hospitals (7.0%) had a basic level, and there was one hospital (0.3%) with an inadequate level. The IPCAF scores per hospital class, region, ownership, and accreditation level are presented in Table 1.

As shown in Table 2, there was a strong relationship between IPCAF score and hospital class. The lowest median IPCAF score (577.5) was found in region 3, or the eastern part of Indonesia. The regulation applied in Indonesia regarding one IPCN responsible for 100 beds was followed by 82.9% of hospitals, and hospitals with sufficient IPCN had significantly higher IPCAF scores. Furthermore, a significant difference in median IPCAF score was found between hospitals with the availability of IPC link nurses (IPCLN) compared to those that did not employ IPCLNs. IPCLNs act as a link between a clinical area and the infection control team and raise awareness of IPC by educating colleagues and motivating staff to improve IPC practice [11]. Private hospitals were associated with the highest median IPCAF score, whereas government-sponsored hospitals had the lowest median IPCAF score. A strong link was also found between IPCAF score and the availability of electronic medical records. National accreditation (any level) was unrelated to the IPCAF score.

**Table 2:** Characteristics of participating hospitals in relation to their IPCAF score

Variable	Subgroup	N	Median IPCAF score	Range (min–max)	*P* value
Hospital Class					< 0.001^a^
	A	8	730.0	565.0–787.5	
	B	98	665.0	351.0–777.5	
	C	165	603.8	252.5–780.0	
	D	85	577.5	200.0–750.0	
Region					0.016^b^
	1 (Java, Sumatra, Bali)	274	626.3	240.0–787.5	
	2 (Sulawesi, Kalimantan, West Nusa Tenggara)	51	605.0	200.0–767.5	
	3 (Maluku, Papua, West Papua, East Nusa Tenggara)	30	577.5	245.0–745.0	
Ownership of hospital					0.006^c^
	Private	198	631.3	335.0–780.0	
	State-owned enterprises	9	606.0	465.0–762.5	
	Government	148	595.0	200.0–787-5	
National Accreditation					0.061
	No	22	525.0	245.0–787.5	
	Yes	333	620.0	200.0–780.0	
Ratio of IPCN: beds (1:100)					0.006
	No	61	567.5	245.0–773.5	
	Yes	294	624.3	200.0–787.5	
Availability of IPCLN					< 0.001
	No	18	473.8	245.0–742.5	
	Yes	337	623.5	200.0–787.5	
Electronic medical records					< 0.001^a^
	No	164	582.5	200.0–767.5	
	Yes, but only in several units	148	637.5	311.0–787.5	
	Yes, in all units	43	702.5	450.0–777.5	

*IPCAF* infection prevention and control assessment framework, *IPCN* infection prevention and control nurse, *IPCLN* infection prevention and control link nurse

^a^Significant difference found between all groups

^b^Significant difference found between region 1 and 3. The mean ± SD IPCAF score per region were: region 1, 606.6 ± 115.3; region 2, 584.0 ±  = 126.5; and region 3, 544.6 ± 125.9

^c^Significant difference found between private and government-owned hospitals

**Fig. 2:**
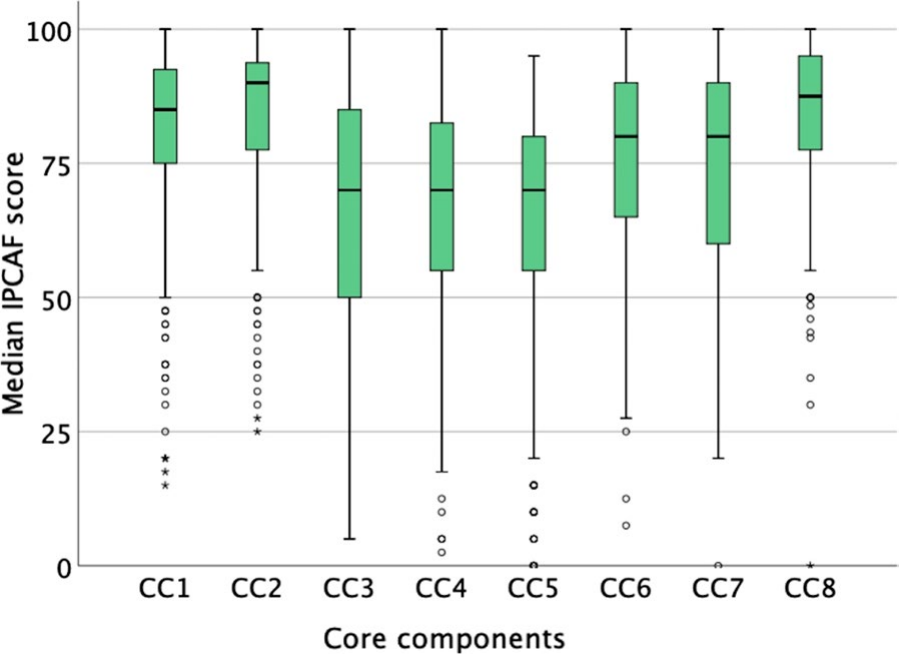
Distribution of results per core component infection prevention and control assessment framework (IPCAF) score. CC1: IPC program, CC2: IPC guidelines, CC3: Education and training, CC4: Surveillance of HAIs, CC5: Multimodal strategies, CC6: Monitoring/audit and feedback, CC7: Workload, staffing and bed occupancy, CC8: Built environment, materials and equipment for IPC

An analysis of scores for each CC is presented in Fig. 2. The highest median score among all CCs was IPC guidelines (CC2) with a median score of 90.0. In addition, multimodal strategies (CC5), education and training (CC3) and surveillance of HAIs (CC4) were the CCs with the lowest median score (70.0 for each).

### Indicators with overall high and low scores

To get more detailed insight into the strengths and gaps in IPC programs in Indonesian hospitals, indicators with a score of > 90% (suggesting a strength) and < 50% (suggesting a gap) were analyzed. In total, 20 out of 81 indicators in the IPCAF questionnaire had > 90% of answers indicative of good IPC implementation (Additional file 10). The indicators regarding guidelines showed that more than 90% of hospitals had nine out of 14 guidelines that should be available in healthcare facilities. Surveillance for HAIs was conducted for surgical site infections and device-associated infections in 93.8% and 92.7% of hospitals, respectively. In total, 72.7% of hospitals diagnose HAIs based only on clinical signs or symptoms in the absence of microbiological testing. In CC6, i.e., monitoring/audit IPC practices and feedback, there are 9 indicators. Four out of the 9 indicators scored over 90%, namely hand hygiene compliance, cleaning the ward environment, disinfection and sterilization of medical equipment/instruments, and waste management.

From all 81 indicators in the IPCAF questionnaire, there were 8 indicators for which < 50% of hospitals provided answers that indicated de facto implementation of the subtopic, suggesting an overall gap in Indonesian healthcare facilities (Additional file 11). Lack of expertise (in IPC and/or infectious diseases) required for developing or adapting guidelines (38%) and to organize and lead IPC training programs (45.4%) were included in this group. Parameters related to microbiology and AMR also scored low, especially in CC4 such as: the availability of adequate microbiology and laboratory capacity to support surveillance of HAIs (43%), surveillance of colonization or infections caused by multidrug-resistant (MDR) pathogens according to the local epidemiological situation (35.8%), the analysis of AMR on a regular basis (for example, quarterly/half-yearly/annually) (33.5%), and in CC6, the monitoring of consumption/usage of antimicrobial agents (31.0%).

### Additional questions

The greatest challenge expressed by most hospitals in Indonesia (median of 3) was communication with the management of the hospitals. Two other challenges that received a high score of priority were the capability to perform Antimicrobial Susceptibility Testing (AST) and sufficient staffing of full-time IPCN (Table 3).

**Table 3:** Identified challenges in implementing IPC in the 355 participating hospitals

Challenges	Median score*
Communication with hospital management	3
Capability to perform AST	4
Sufficient staffing of full-time IPCN	5
Funding for the IPC programs and activities	6
Sterilization and disinfection	6
Facilities (microbiology laboratory, incinerator, logistic support, solid waste treatment plant, internet, and hand hygiene facilities)	7
Dissemination of IPC programs, guidelines of IPC and update of the IPC knowledge	7
Surveillance of HAIs	7
Training and education in IPC	7
Changes in organization	7
Changes in behavior of HCWs	7

Hospitals were provided 11 challenges in the questionnaire, and each hospital was asked to rank these challenges from high to low priority based on the situation in their hospital

*A lower (median) score corresponds to a challenge of higher priority

*AST* antimicrobial susceptibility testing, *HAIs* healthcare-associated infections, *HCW* healthcare worker, *IPCN* infection prevention and control nurse

### Interviews with the management representatives of 38 hospitals

Interviews were held with management representatives of 38 hospitals (10% of participating hospitals with one additional class A hospital). Overall, 68.4% of senior management (medical director, head of medical division, head of nurse division) joined the interview, with 26.3% being the director of the hospital (Additional file 12). More than half of the management (55.3%) joined IPC meetings at least once in the three months preceding the interview. Further results of the interviews with the management are available in Additional file 13.

For the hospital level, the most frequently mentioned recommendation by the management was to have IPC committee members regularly join IPC training (36.8%). The top recommendation for the national level was to provide more free and online training for all hospitals (34.4%). Recommendations for both hospital and national level can be found in Table 4.

**Table 4:** Recommendations on IPC implementation on a hospital and a national level based on the interviews with the management of participating hospitals (38 hospitals)

Recommendations on hospital level	N (%)	Recommendations on national level	N (%)
Update knowledge, especially about regulations and guidelines for IPC committee members by participating in IPC training regularly	14 (36.8)	Provide more national training (free and online) for all hospitals, including private hospitals and hospitals in the eastern part of Indonesia	22 (57.9)
Additional funding for IPC (IPC budget is still joined with other units, especially for participation in training programs)	11 (28.9)	Update of the IPC guidelines/regulations and dissemination to all hospitals	10 (26.3)
Adding more personnel in IPC committees (full-time IPCN, IPCLN, and training new IPCN)	10 (26.3)	Additional funding for IPC	8 (21.1)
Raise more awareness, increase compliance, and improve implementation of IPC	9 (23.7)	Guidance and regular evaluation of IPC implementations in hospitals	7 (18.4)
Enhance the coordination with other units, including management	9 (23.7)	Improve facilities (e.g., availability and capability of microbiology laboratory, incinerator, logistic support, solid waste treatment plant, internet, and hand hygiene facilities)	6 (15.8)
Promotion and dissemination of IPC programs to all personnel in hospital (bundles, preparing videos or posters)	5 (13.2)	Implement program that promotes IPC program continuously (e.g., hand hygiene week)	5 (13.2)
Improve facilities and infrastructure (e.g., availability and capability of microbiology laboratory, waste water, and solid waste treatment plant)	5 (13.2)	Consider the capacity of local hospitals when implementing a new program (e.g., conduct a feasibility study before introducing a new program, including the budget for implementation, into the regional allocation fund)	2 (5.3)
Monitoring and evaluation of IPC program	3 (7.9)	Requirements related to IPC program should be adapted to local conditions (e.g., requirements for IPCN, license for waste treatment plant)	2 (5.3)
Develop IPC program tailored to the conditions in the hospital	3 (7.9)	Guidelines for PPE should be integrated between governments, occupational organization	1 (2.6)
Add more IPC trainers by having staff join IPC training programs	2 (5.3)	Integrated program within or between local and central government to avoid redundant projects	1 (2.6)
Optimize follow-up of recommendation of IPC	2 (5.3)		
Availability of electronic information system (especially for HAI surveillance and medical records)	2 (5.3)		

*IPCN* infection prevention and control nurse, *IPCLN* infection prevention and control link nurse, *HAIs* healthcare-associated infections, *PPE* personnel protective equipment

### Interviews with the IPC committee/team

Interviews were also performed with the IPC committee/team from the same hospitals as the management, with IPCNs comprising the largest fraction (39.5%) of interviewees (Additional file 14). Based on the overall results of the IPCAF questionnaires (Fig. 2), interviews were focused on the three CCs that had the lowest scores, namely education and training (CC3), surveillance of HAIs (CC4), and multimodal strategies (CC5). The biggest challenge faced for education and training (CC3) was lack of funding. Availability of a microbiology laboratory and culture facilities was the biggest challenge for surveillance of HAIs (CC4). Lastly, difficulty in behavioral changes was a considerable challenge in multimodal strategies (CC5) (Table 5).

**Table 5:** Challenges in implementing IPC per CC identified through interviews with the IPC committee/team of 38 hospitals

CC3. Education and training	N (%)	CC4. Surveillance of HAIs	N (%)	CC5. MMS	N (%)
Lack of funding	29 (76.3)	Microbiology laboratory and culture unavailable	13 (34.2)	Difficulties in changing behavior	18 (47.4)
Awareness of and compliance in implementing IPC	13 (34.2)	Awareness and compliance (i.e., lag in data collection and reporting)	11 (28.9)	Lack of funding	14 (36.8)
Lack of human resources; double job and there is no full time IPCN	11 (28.9)	Lack of opportunities to join surveillance training	10 (26.3)	Awareness and compliance (e.g., hand hygiene and safety injection)	14 (36.8)
Difficulty in changing behavior	10 (26.3)	Lack of coordination between IPCN, IPCLN, management, and quality committee	7 (18.4)	Lack of support from management	8 (21.1)
Limited time to join training and low attendance	8 (21.1)	Lack of funding	6 (15.8)	Facilities and infrastructure are still inadequate	8 (21.1)
Facilities and infrastructure are inadequate	7 (18.4)	Lack of data collection and reporting	6 (15.8)	Limited knowledge about MMS	5 (13.2)
Lack of support from management	6 (15.8)	Facilities and infrastructure are still inadequate	5 (13.2)	Lack of human resources (double job)	5 (13.2)
Lack of IPC training opportunities	6 (15.8)	Lack of support from management	5 (13.2)	Lack of IPC training opportunities	5 (13.2)
Lack of trainer (internal or external)	6 (15.8)	No full-time IPCN	5 (13.2)	No full-time IPCN	4 (10.5)
No full-time IPCN	5 (13.2)	Discrepancy between clinician and IPCN in diagnosing HAIs	5 (13.2)	Lack of coordination between hospital departments and IPC committee	4 (10.5)

*CC* core component, *HAIs* healthcare-associated infections, *MMS* multimodal strategies, *IPCN* infection prevention and control nurse, *IPCLN* infection prevention and control link nurse

## Discussion

This first national study among 355 Indonesian general hospitals showed an overall advanced level of IPC program implementation. Despite of its status as lower-middle-income country, the overall implementation of IPC in Indonesia is on a level that is more comparable to some high-income countries such as Austria and Germany [12, 13]. Our results showed that the level of IPC in Indonesia is higher compared to other LMIC, with both Bangladesh and Ghana showing a basic level of IPC structure on a hospital level [14, 15]. However, the range of the scores was wide, indicating that there is need for further improvement in terms of more ubiquitous implementation and enforcement of high-quality IPC programs.

Specifically, there was a significant difference in IPCAF score between region 1 (Java, Sumatra, and Bali) and region 3 (Maluku, Papua, West Papua, and East Nusa Tenggara), with the scores in the eastern Indonesia (region 3) being more comparable to the LMIC scores in the global survey. Consequently, the uneven distribution in the level of IPC implementation across the country should be addressed.

Results from the 8 CCs in studies from Bangladesh, India, Germany, and Austria [12, 13, 16, 17] and the global survey [18] are comparable to the outcomes of our study with respect to IPC guidelines (CC2) scoring the highest. Although almost all hospitals in Indonesia have guidelines, the necessary IPC expertise to properly adopt and implement the guidelines is lacking. Therefore, IPC training programs should cover this problem to achieve better IPC implementation.

The three lowest scoring CCs were multimodal strategies, surveillance of HAIs, and education and training. This outcome echoes the results of national IPCAF studies from Bangladesh, Germany and Austria [12–14]. Multimodal strategies are strongly recommended by the WHO as the most effective approach to improve IPC practices [19]. This newly introduced term is only known by 21.9% of IPC committees or teams in hospitals in Indonesia. Therefore, training on and dissemination of MMS need to be conducted. The greatest challenge for implementation of MMS is the difficulty in changing the behavior of HCWs. This is in line with multiple global studies, which also revealed that changing behavior is the most significant hurdle in implementing IPC [20–22]. Culturally, leadership plays an important role in Indonesia [23]. Therefore, managers and decision makers play a key role in prioritizing IPC in hospitals so that better implementation of IPC using MMS can be achieved.

The low score of the CC regarding surveillance of HAIs in our study has also been observed in other LMICs [21, 22]. Nevertheless, several studies showed that routine surveillance is effective in reducing the number of HAIs [24–27]. Hospitals are lacking access to basic microbiology infrastructure to diagnose HAIs, so hospitals rely on clinical symptoms only for diagnosis, which is subpar (see Additional file 14). Improvements in microbiology laboratory availability and the capacity of conducting antimicrobial susceptibility testing in the hospitals is needed.

More attention should be given to education and training. Particularly, the lack of personnel with IPC expertise to lead IPC training programs should be addressed. This challenge was also found in Georgia, Ghana, and Bangladesh [10, 14, 15]. Another study from Bangladesh showed that formal training on IPC was absent in 85% of the HCWs [28]. A study in European countries showed that there should be a national training program with learning objectives and support from national professional bodies [29]. Indonesia does not have a harmonized national, structured training program available. Therefore, IPC training is not standardized, and this situation needs to be strengthened. Lack of funding for education remains the greatest challenge in this regard. However, since the COVID-19 pandemic, online training has become more common. This creates an opportunity to limit organization costs and allows inclusion of hospitals from more/all regions of Indonesia. The presence of an electronic medical record came with a higher IPCAF score. This most likely reflects a higher quality standard that thus affects the level of IPC in hospitals.

From the interviews, communication between IPC committees and teams with the management of the hospitals was identified as the greatest challenge. Results from a study from McAlearney et al*.* indicated that strategic communication played an important role to support IPC programs [30]. Effective ways to communicate between IPC committees and teams with the management of the hospitals should be sought.

This study showed that full-time IPCNs are lacking, similar to the condition in other LMICs [21] as well as in the Middle-East and Afghanistan [31]. Even though guidelines recommend one IPCN per 100 beds, employment of IPCNs in hospitals warrants further improvement. The current study unveiled those hospitals with sufficient IPCN and/or IPCLN staff have higher IPCAF scores, which suggests that having full-time IPC(L)Ns is critical to optimal IPC implementation.

## Strengths and limitations of the study

This is the first IPCAF study performed in Indonesia. Another strength of this study is we included analyses for separate hospital classes and regions separately. Third, before actual data collection, a pilot study was conducted in the four classes of hospitals that were excluded from the main study to validate all questionnaires in Indonesian language. The WHO IPCAF was translated and adjusted to the situation in hospitals in Indonesia. This allowed the hospitals to answer the questionnaires in the native language and minimized misunderstanding and miscommunication. Answering each question was mandatory, therefore all submitted questionnaires were complete. To ensure the correct interpretations of the questionnaires and to minimize any bias, a pilot study, introduction meeting, reminders, and all forms of communication (email, WhatsApp, text, telephone) were conducted to assist hospitals in answering the questionnaires. Reminders were sent every week to achieve a high percentage of response rate. Another strength is the addition of interviews with hospital management and IPC committees to elaborate on the challenges in implementing IPC in hospitals.

Despite these strong elements, our study came with limitations. First, IPC implementation is a sensitive issue among hospitals, as a low scoring hospital may be associated with low-quality performance. Therefore, there is a chance of response bias in the answers received in the questionnaire and interviews. To reduce this bias we emphasized, during the introduction meeting, that the results of this study would not affect the hospital’s accreditation nor reputation. Second, a qualitative analysis method would have been the ideal way to analyse the results from semi structured interviews. However, we considered our quantitative approach, in which we presented the results of the semi-structured interviews with frequency or a count of responses appropriate in the context of the intended purposes of this study. Lastly, the WHO IPCAF is a robust tool equipped with full explanations and footnotes for each question [3]. Since the survey is essentially a self-assessment conducted online, there may be some misinterpreted questions due to the variety in one’s ability of the subject to understand the question as well as subjectivity in some of the responses. Hence, there is a possibility that some answers do not reflect the actual situation in hospitals.

## Conclusions

IPC is generally well implemented in Indonesian hospitals with the provision of IPC guidelines as a strength. Potential areas of improvement are MMS, surveillance of HAIs, and education and training. Communication with the management of the hospitals, capability to perform AST and sufficient staffing of IPCN were found as gaps that need to be improved. We strongly advise all healthcare facilities to conduct self-assessments using the translated questionnaires regularly (for example, once a year) to identify the strengths, gaps, and challenges in the implementation of IPC. The results of these self-assessments can be used in all healthcare facilities, including hospitals, to formulate strategies for interventions. From such regular surveys, trends of gaps can be analyzed with root cause analyses. In addition, our approach of combining the IPCAF with additional interviews would be useful for other countries as well.

Based on the IPCAF questionnaires, additional questions on challenges and recommendations, and interviews we identified the following key priorities for further IPC implementation and improvement in Indonesian hospitals: (1) focus on the eastern part of Indonesia, (2) improved access to basic microbiology laboratories, (3) more funding should be made available for IPC training—more specifically for both IPC(L)Ns and the larger healthcare worker population, (4) recruitment of more IPC(L)Ns and development a roadmap for IPC(L)N professionalization, and (5) hospital management along with regular trainings for different levels of HCWs should be enhanced.

## Supplementary Information

** Additional file 1. **Eight core components (CCs) of IPCAF. **Additional file 2. **Classification of hospitals in Indonesia. **Additional file 3.** IPCAF questionnaires (in English and Bahasa Indonesia). **Additional file 4.** Additional questions. **Additional file 5. **Questionnaires for the management of the hospitals (English and Bahasa Indonesia). **Additional file 6. **Questionnaires for IPC team/committee (English and Bahasa Indonesia). **Additional file 7.** Flow of the study. **Additional file 8.** Category of IPCAF score. **Additional file 9.** Total IPCAF score. **Additional file 10.** Core components with a score more than  90%. **Additional file 11.** Core components with a score less than 50%. **Additional file 12.** Characteristics of the interviewed hospitals and interviewees (N = 38 hospitals). **Additional file 13.** Interviews with the management of the hospitals (N = 38 hospitals). **Additional file 14.** Interviews with the IPC committee/team of the hospitals (N = 38 hospitals).

## Data Availability

The datasets generated and/or analyzed during the current study are not publicly available due to privacy of the participating hospitals.

## Abbreviations

IPC: Infection prevention and control
WHO: World Health Organization
IPCAF: Infection Prevention and Control Assessment Framework
HAIs: Healthcare-associated infections
LMICs: Low- and middle-income countries
AMR: Antimicrobial resistance
CCs: Core components
IPCN: Infection prevention and control nurse
IPCLN: Infection prevention and control link nurse
MMS: Multimodal strategies
LPDP: Lembaga Pengelola Dana Pendidikan (Indonesian Endowment Fund for Education)
SD: Standard deviation
