# Supplementary material for: Infection prevention and control in Indonesian hospitals: identification of strengths, gaps, and challenges
Source: Antimicrob Resist Infect Control. 2023 Feb 3;12:6. doi: 10.1186/s13756-023-01211-5 (PMC9894741; doi:10.1186/s13756-023-01211-5)
Supplement: Supplementary file 1 — Additional file 1. Eight core components (CCs) of IPCAF. Additional file 2. Classification of hospitals in Indonesia. Additional file 3. IPCAF questionnaires (in English and Bahasa Indonesia). Additional file 4. Additional questions. Additional file 5. Questionnaires for the management of the hospitals (English and Bahasa Indonesia). Additional file 6. Questionnaires for IPC team/committee (English and Bahasa Indonesia). Additional file 7. Flow of the study. Additional file 8. Category of IPCAF score. Additional file 9. Total IPCAF score. Additional file 10. Core components with a score more than 90%. Additional file 11. Core components with a score less than 50%. Additional file 12. Characteristics of the interviewed hospitals and interviewees (N = 38 hospitals). Additional file 13. Interviews with the management of the hospitals (N = 38 hospitals). Additional file 14. Interviews with the IPC committee/team of the hospitals (N = 38 hospitals). [file 13756_2023_1211_MOESM1_ESM.docx]

**Additional file 1. Eight core components (CCs) of Infection Prevention Control Assessment Framework (IPCAF)**

| **CC** | **Details** |
| --- | --- |
| 1 | Infection prevention control (IPC) program |
| 2 | IPC guidelines |
| 3 | Education and training |
| 4 | Healthcare-associated infection (HAI) surveillance |
| 5 | Multimodal strategies |
| 6 | Monitoring/audit and feedback |
| 7 | Workload, staffing, and bed occupancy |
| 8 | Built environment, materials and equipment for IPC |

**Additional file 2. Classification of hospitals in Indonesia**

|  | **Class A** | **Class B** | **Class C** | **Class D** |
| --- | --- | --- | --- | --- |
| Beds | ≥250 | 200-249 | 100-199 | 50-99 |
| Services | Specialist and sub-specialist | Specialist and sub-specialist | Specialist only | Specialist only |

**Additional file 3. IPCAF questionnaires (in English and Bahasa Indonesia)**

**English version**

**Core component 1:** **Infection Prevention and Control (IPC) program**

| **Question** | **Score** |
| --- | --- |
| **1. Do you have an IPC program?**  No  Yes, without clearly defined objectives  Yes, with clearly defined objectives and annual activity plan  **2. Is the IPC program supported by an IPC team comprising of IPC professionals?**  No  Not a team, only an IPC focal person  Yes  **3. Does the IPC team have at least one full-time IPC professional or equivalent (nurse or doctor working 100% in IPC) available?**  No IPC professional available  No, only a part-time IPC professional available  Yes, one per > 100 beds  Yes, one per ≤ 100 beds  **4. Does the IPC team or focal person have dedicated time for IPC activities?**  No  Yes  **5. Does the IPC team include both doctors and nurses?**  No  Yes  **6. Do you have an IPC committee actively supporting the IPC team?**  No  Yes  **7. Are any of the following professional groups represented/included in the IPC committee?**  **- Senior facility leadership (for example, administrative director, chief executive officer [CEO], medical director)**  No  Yes  **- Senior clinical staff (for example, physician, nurse)**  No  Yes  **- Facility management (for example, biosafety, waste, and those tasked with addressing water, sanitation, and hygiene [WASH])**  No  Yes  **8. Do you have clearly defined IPC objectives (that is, in specific critical areas)?**  No  Yes, IPC objectives only  Yes, IPC objectives and measurable outcome indicators (that is, adequate measures for improvement)  Yes, IPC objectives, measurable outcome indicators and set future targets  **9. Does the senior facility leadership show clear commitment and support for the IPC program:**  **- By an allocated budget specifically for the IPC program (that is, covering IPC activities, including salaries)?**  No  Yes  **- By demonstrable support for IPC objectives and indicators within the facility (for example, at executive level meetings, executive rounds, participation in morbidity and mortality meetings)?**  No  Yes  **10. Does your facility have microbiological laboratory support (either present on or off site) for routine day-to-day use?**  No  Yes, but not delivering results reliably (timely and of sufficient quality)  Yes, and delivering results reliably (timely and of sufficient quality) | 0  5  10  0  5  10  0  2.5  5  10  0  10  0  10  0  10  0  5  0  2.5  0  2.5  0  2.5  5  10  0  5  0  5  0  5  10 |

**Core component 2:** **Infection Prevention and Control (IPC) guidelines**

| **Question** | **Score** |
| --- | --- |
| **1. Does your facility have the expertise (in IPC and/or infectious diseases) for developing or adapting guidelines?**  No  Yes  **2. Does your facility have guidelines available for:**  **- Standard precautions?**  No  Yes  **- Hand hygiene?**  No  Yes  **- Transmission-based precautions?**  No  Yes  **- Outbreak management and preparedness?**  No  Yes  **- Prevention of surgical site infection?**  No  Yes  **- Prevention of vascular catheter-associated bloodstream infections?**  No  Yes  **- Prevention of hospital-acquired pneumonia ([HAP]; all types of HAP, including (but not exclusively) ventilator-associated pneumonia)?**  No  Yes  **- Prevention of catheter-associated urinary tract infections?**  No  Yes  **- Prevention of transmission of multidrug-resistant (MDR) pathogens?**  No  Yes  **- Disinfection and sterilization?**  No  Yes  **- Health care worker protection and safety**  No  Yes  **- Injection safety?**  No  Yes  **- Waste management?**  No  Yes  **- Antibiotic stewardship?**  No  Yes  **3. Are the guidelines in your facility consistent with national/international guidelines (if they exist)?**  No  Yes  **4. Is implementation of the guidelines adapted according to the local needs and resources while maintaining key IPC standards?**  No  Yes  **5. Are frontline health care workers involved in both planning and executing the implementation of IPC guidelines in addition to IPC personnel?**  No  Yes  **6. Are relevant stakeholders (for example, lead doctors and nurses, hospital managers, quality management) involved in the development and adaptation of the IPC guidelines in addition to IPC personnel?**  No  Yes  **7. Do health care workers receive specific training related to new or updated IPC guidelines introduced in the facility?**  No  Yes  **8. Do you regularly monitor the implementation of at least some of the IPC guidelines in your facility?**  No  Yes | 0  7.5  0  2.5  0  2.5  0  2.5  0  2.5  0  2.5  0  2.5  0  2.5  0  2.5  0  2.5  0  2.5  0  2.5  0  2.5  0  2.5  0  2.5  0  10  0  10  0  10  0  10  0  7.5  0  10  0  10 |

**Core component 3:** **Infection Prevention and Control (IPC) education and training**

| **Question** | **Score** |
| --- | --- |
| **1. Are there personnel with the IPC expertise (in IPC and/or infectious diseases) to lead IPC training?**  No  Yes  **2. Are there additional non-IPC personnel with adequate skills to serve as trainers and mentors (for example, link nurses or doctors, champions)?**  No  Yes  **3. How frequently do health care workers receive training regarding IPC in your facility?**  Never or rarely  New employee orientation only for health care workers  New employee orientation and regular (at least annually) IPC training for health care workers offered but not mandatory  New employee orientation and regular (at least annually) mandatory IPC training for all health care workers  **4. How frequently do cleaners and other personnel directly involved in patient care receive training regarding IPC in your facility?**  Never or rarely  New employee orientation only for other personnel  New employee orientation and regular (at least annually) training for other personnel offered but not mandatory  New employee orientation and regular (at least annually) mandatory IPC training for other personnel  **5. Does administrative and managerial staff receive general training regarding IPC in your facility?**  No  Yes  **6. How are health care workers and other personnel trained?**  No training available  Using written information and/or oral instruction and/or e-learning only  Includes additional interactive training sessions (for example, simulation and/or bedside training)  **7. Are there periodic evaluations of the effectiveness of training programs (for example, hand hygiene audits, other checks on knowledge)?**  No  Yes, but not regularly  Yes, regularly (at least annually)  **8. Is IPC training integrated in the clinical practice and training of other specialties (for example, training of surgeons involves aspects of IPC)?**  No  Yes, in some disciplines  Yes, in all disciplines  **9. Is there specific IPC training for patients or family members to minimize the potential for health care-associated infections (for example, immunosuppressed patients, patients with invasive devices, patients with multidrug-resistant infections)?**  No  Yes  **10. Is ongoing development/education offered for IPC staff (for example, by regularly attending conferences, courses)?**  No  Yes | 0  10  0  10  0  5  10  15  0  5  10  15  0  5  0  5  10  0  5  10  0  5  10  0  5  0  10 |

**Core component 4: Health care-associated infection (HAI) surveillance**

| **Question** | **Score** |
| --- | --- |
| **1. Is surveillance a defined component of your IPC program?**  No  Yes  **2. Do you have personnel responsible for surveillance activities?**  No  Yes  **3. Have the professionals responsible for surveillance activities been trained in basic epidemiology, surveillance and IPC (that is, capacity to oversee surveillance methods, data management and interpretation)?**  No  Yes  **4. Do you have informatics/IT support to conduct your surveillance (for example, equipment, mobile technologies, electronic health records)?**  No  Yes  **5. Do you go through a prioritization exercise to determine the HAIs to be targeted for surveillance according to the local context (that is, identifying infections that are major causes of morbidity and mortality in the facility)?**  No  Yes  **6. In your facility is surveillance conducted for:**  **- Surgical site infections?**  No  Yes  **- Device-associated infections (for example, catheter-associated urinary tract infections, central line-associated bloodstream infections, peripheral-line associated bloodstream infections, ventilator-associated pneumonia)?**  No  Yes  **- Clinically-defined infections (for example, definitions based only on clinical signs or symptoms in the absence of microbiological testing)?**  No  Yes  **- Colonization or infections caused by multidrug-resistant pathogens according to your local epidemiological situation?**  No  Yes  **- Local priority epidemic-prone infections (for example, norovirus, influenza, tuberculosis [TB], severe acute respiratory syndrome [SARS], Ebola, Lassa fever)?**  No  Yes  **- Infections in vulnerable populations (for example, neonates, intensive care unit, immunocompromised, burn patients)?**  No  Yes  **- Infections that may affect health care workers in clinical, laboratory, or other settings (for example, hepatitis B or C, human immunodeficiency virus [HIV], influenza)?**  No  Yes  **7. Do you regularly evaluate if your surveillance is in line with the current needs and priorities of your facility?**  No  Yes  **8. Do you use reliable surveillance case definitions (defined numerator and denominator according to international definitions [e.g., CDC NHSN/ECDC] or if adapted, through an evidence-based adaptation process and expert consultation?**  No  Yes  **9. Do you use standardized data collection methods (for example, active prospective surveillance) according to international surveillance protocols (for example, CDC NHSN/ECDC) or if adapted, through an evidence-based adaptation process and expert consultation?**  No  Yes  **10. Do you have processes in place to regularly review data quality (for example, assessment of case report forms, review of microbiology results, denominator determination, etc.)?**  No  Yes  **11. Do you have adequate microbiology and laboratory capacity to support surveillance?**  No  Yes, can differentiate gram-positive/negative strains but cannot identify pathogens  Yes, can reliably identify pathogens (for example, isolate identification) in a timely manner  Yes, can reliably identify pathogens and antimicrobial drug resistance patterns (that is, susceptibilities) in a timely manner  **12. Are surveillance data used to make tailored unit/facility-based plans for the improvement of IPC practices?**  No  Yes  **13. Do you analyze antimicrobial drug resistance on a regular basis (for example, quarterly/half-yearly/annually)?**  No  Yes  **14. Do you regularly (for example, quarterly/half-yearly/annually) feedback up-to-date surveillance information to:**  **- Frontline health care workers (doctors/nurses)?**  No  Yes  **- Clinical leaders/heads of department**  No  Yes  **- IPC committee**  No  Yes  **- Non-clinical management/administration (chief executive officer/chief financial officer)?**  No  Yes  **15. How do you feedback up-to-date surveillance information? (at least annually)**  No feedback  By written/oral information only  By presentation and interactive problem-orientated solution finding | 0  5  0  5  0  5  0  5  0  5  0  2.5  0  2.5  0  2.5  0  2.5  0  2.5  0  2.5  0  2.5  0  5  0  5  0  5  0  5  0  2.5  5  10  0  5  0  5  0  2.5  0  2.5  0  2.5  0  2.5  0  2.5  7.5 |

**Core component 5: Multimodal strategies for implementation of infection prevention and control (IPC) interventions**

| **Question** | **Score** |
| --- | --- |
| **1. Do you use multimodal strategies to implement IPC interventions?**  No  Yes  **2. Do your multimodal strategies include any or all of the following elements:**  **- System change**  Element not included in multimodal strategies  Interventions to ensure the necessary infrastructure and continuous availability of supplies are in place  Interventions to ensure the necessary infrastructure and continuous availability of supplies are in place and addressing ergonomics and accessibility, such as the best placement of central venous catheter set and tray  **- Education and training**  Element not included in multimodal strategies  Written information and/or oral instruction and/or e-learning only  Additional interactive training sessions (includes simulation and/or bedside training)  **- Monitoring and feedback**  Element not included in multimodal strategies  Monitoring compliance with process or outcome indicators (for example, audits of hand hygiene or catheter practices)  Monitoring compliance and providing timely feedback of monitoring results to health care workers and key players  **- Communications and reminders**  Element not included in multimodal strategies  Reminders, posters, or other advocacy/awareness-raising tools to promote the intervention  Additional methods/initiatives to improve team communication across units and disciplines (for example, by establishing regular case conferences and feedback rounds)  **- Safety climate and culture change**  Element not included in multimodal strategies  Managers/leaders show visible support and act as champions and role models, promoting an adaptive approach and strengthening a culture that supports IPC, patient safety and quality  Additionally, teams and individuals are empowered so that they perceive ownership of the intervention (for example, by participatory feedback rounds)  **3. Is a multidisciplinary team used to implement IPC multimodal strategies?**  No  Yes  **4. Do you regularly link to colleagues from quality improvement and patient safety to develop and promote IPC multimodal strategies?**  No  Yes  **5. Do these strategies include bundles or checklists?**  No  Yes | 0  15  0  5  10  0  5  10  0  5  10  0  5  10  0  5  10  0  15  0  10  0  10 |

**Core component 6: Monitoring/audit of IPC practices and feedback**

| **Question** | **Score** |
| --- | --- |
| **1. Do you have trained personnel responsible for monitoring/audit of IPC practices and feedback?**  No  Yes  **2. Do you have a well-defined monitoring plan with clear goals, targets and activities (including tools to collect data in a systematic way)?**  No  Yes  **3. Which processes and indicators do you monitor in your facility?** (Tick all that apply)  None  Hand hygiene compliance (using the WHO hand hygiene observation tool or equivalent)  Intravascular catheter insertion and/or care  Wound dressing change  Transmission-based precautions and isolation to prevent the spread of multidrug resistant organisms (MDRO)  Cleaning of the ward environment  Disinfection and sterilization of medical equipment/instruments  Consumption/usage of alcohol-based handrub or soap  Consumption/usage of antimicrobial agents  Waste management  **4. How frequently is the WHO Hand Hygiene Self-Assessment Framework Survey undertaken?**  Never  Periodically, but no regular schedule  At least annually  **5. Do you feedback auditing reports (for example, feedback on hand hygiene compliance data or other processes) on the state of the IPC activities/performance?** (Tick all that apply)  No reporting  Yes, within the IPC team  Yes, to department leaders and managers in the areas being audited  Yes, to frontline health care workers  Yes, to the IPC committee or quality of care committees or equivalent  Yes, to hospital management and senior administration  **6. Is the reporting of monitoring data undertaken regularly (at least annually)?**  No  Yes  **7. Are monitoring and feedback of IPC processes and indicators performed in a “blame-free” institutional culture aimed at improvement and behavioural change?**  No  Yes  **8. Do you assess safety cultural factors in your facility (for example, by using other surveys such as HSOPSC, SAQ, PSCHO, HSC)**  No  Yes | 0  10  0  7.5  0  5  5  5  5  5  5  5  5  5  0  2.5  5  0  2.5  2.5  2.5  2.5  2.5  0  10  0  5  0  5 |

**Core component 7: Workload, staffing and bed occupancy**

| **Question** | **Score** |
| --- | --- |
| **1. Are appropriate staffing levels assessed in your facility according to patient workload using national standards or a standard staffing needs assessment tool such as the WHO Workload indicators of staffing need method?**  No  Yes  **2. Is an agreed (that is, WHO or national) ratio of health care workers to patients maintained across your facility?**  No  Yes, for staff in less than 50% of units  Yes, for staff in more than 50% of units  Yes, for all health care workers in the facility  **3. Is a system in place in your facility to act on the results of the staffing needs assessments when staffing levels are deemed to be too low?**  No  Yes  **4. Is the design of wards in your facility in accordance with international standards regarding bed capacity?**  No  Yes, but only in certain departments  Yes, for all departments (including emergency department and pediatrics)  **5. Is bed occupancy in your facility kept to one patient per bed?**  No  Yes, but only in certain departments  Yes, for all units (including emergency departments and pediatrics)  **6. Are patients in your facility placed in beds standing in the corridor outside of the room (including beds in the emergency department)?**  Yes, more frequently than twice a week  Yes, less frequently than twice a week  No  **7. Is adequate spacing of > 1 meter between patient beds ensured in your facility?**  No  Yes, but only in certain departments  Yes, for all departments (including emergency department and pediatrics)  **8. Is a system in place in your facility to assess and respond when adequate bed capacity is exceeded?**  No  Yes, this is the responsibility of the head of department  Yes, this is the responsibility of the hospital administration/management | 0  5  0  5  10  15  0  10  0  5  15  0  5  15  0  5  15  0  5  15  0  5  10 |

**Core component 8: Built environment, materials and equipment for IPC at the facility level**

| **Question** | **Score** |
| --- | --- |
| **1. Are water services available at all times and of sufficient quantity for all uses (for example, hand washing, drinking, personal hygiene, medical activities, sterilization, decontamination, cleaning and laundry)?**  No, available on average < 5 days per week  Yes, available on average ≥ 5 days per week or every day but not of sufficient quantity  Yes, every day and of sufficient quantity  **2. Is a reliable safe drinking water station present and accessible for staff, patients and families at all times and in all locations/wards?**  No, not available  Sometimes, or only in some places or not available for all users  Yes, accessible at all times and for all wards/groups  **3. Are functioning hand hygiene stations (that is, alcohol-based handrub solution or soap and water and clean single-use towels) available at all points of care?**  No, not present  Yes, stations present, but supplies are not reliably available  Yes, with reliably available supplies  **4. In your facility, are ≥ 4 toilets or improved latrines available for outpatient settings or ≥ 1 per 20 users for inpatient settings?**  Less than required number of toilets or latrines available and functioning  Sufficient number present but not all functioning  Sufficient number present and functioning  **5. In your health care facility, is sufficient energy/power supply available at day and night for all uses (for example, pumping and boiling water, sterilization and decontamination, incineration or alternative treatment technologies, electronic medical devices, general lighting of areas where health care procedures are performed to ensure safe provision of health care and lighting of toilet facilities and showers)?**  No  Yes, sometimes or only in some of the mentioned areas  Yes, always and in all mentioned areas  **6. Is functioning environmental ventilation (natural or mechanical) available in patient care areas?**  No  Yes  **7. For floors and horizontal work surfaces, is there an accessible record of cleaning, signed by the cleaners each day?**  No record of floors and surfaces being cleaned  Record exists, but is not completed and signed daily or is outdated  Yes, record completed and signed daily  **8. Are appropriate and well-maintained materials for cleaning (for example, detergent, mops, buckets, etc.) available?**  No materials available  Yes, available but not well maintained  Yes, available and well-maintained  **9. Do you have single patient rooms or rooms for cohorting patients with similar pathogens if the number of isolation rooms is insufficient (for example, TB, measles, cholera, Ebola, SARS)?**  No  No single rooms but rather rooms suitable for patient cohorting available  Yes, single rooms are available  **10. Is PPE available at all times and in sufficient quantity for all uses for all health care workers?**  No  Yes, but not continuously available in sufficient quantities  Yes, continuously available in sufficient quantities  **11. Do you have functional waste collection containers for non-infectious (general) waste, infectious waste and, sharps waste in close proximity to all waste generation points?**  No bins or separate sharps disposal  Separate bins present but lids missing or more than 3/4 full; only two bins (instead of three); or bins at some but not all waste generation points  Yes  **12. Is a functional burial pit/fenced waste dump or municipal pick-up available for disposal of non-infectious (non-hazardous/general waste)?**  No pit or other disposal method used  Pit in facility but insufficient dimensions; pits/dumps overfilled or not fenced/locked; or irregular municipal waste pick up  Yes  **13. Is an incinerator or alternative treatment technology for the treatment of infectious and sharp waste (for example, an autoclave) present (either present on or off site and operated by a licensed waste management service), functional and of a sufficient capacity?**  No, none present  Present, but not functional  Yes  **14. Is a wastewater treatment system (for example, septic tank followed by drainage pit) present (either on or off site) and functioning reliably?**  No, not present  Yes, but not functioning reliably  Yes and functioning reliably  **15. Does your health care facility provide a dedicated decontamination area and/or sterile supply department (either present on or off site and operated by a licensed decontamination management service) for the decontamination and sterilization of medical devices and other items/equipment?**  No, not present  Yes, but not functioning reliably  Yes and functioning reliably  **16. Do you reliably have sterile and disinfected equipment ready for use?**  No, available on average < five days per week  Yes, available on average ≥ five days per week or every day, but not of sufficient quantity  Yes, available every day and of sufficient quantity  **17. Are disposable items available when necessary? (for example, injection safety devices, examination gloves)**  No, not available  Yes, but only sometimes available  Yes, continuously available | 0  2.5  7.5  0  2.5  7.5  0  2.5  7.5  0  2.5  7.5  0  2.5  5  0  5  0  2.5  5  0  2.5  5  0  2.5  7.5  0  2.5  7.5  0  2.5  5  0  2.5  5  0  1  5  0  2.5  5  0  2.5  5  0  2.5  5  0  2.5  5 |

**Translated IPCAF questionnaire (Bahasa Indonesia version)**

**Komponen Inti 1: Program Pencegahan dan Pengendalian Infeksi (PPI)**

| **Pertanyaan** | **Jawaban** | **Skor** |
| --- | --- | --- |
| **1. Apakah Anda memiliki program PPI*?**  Pilih satu jawaban  *Program PPI merupakan kegiatan terkait PPI di rumah sakit yang memiliki: a. tujuan yang jelas berdasarkan prioritas sesuai dengan data, penilaian risiko, dan data dan situasi di rumah sakit tersebut; b. fungsi target yang ditetapkan sejalan dengan pencegahan infeksi nosokomial atau health care-associated infections (HAIs) dan resistensi antimikroba dirumah sakit; c. Expert/ahli di bidang PPI yang berdedikasi dan terlatih. Untuk lebih lengkapnya dapat melihat the WHO Guidelines on core components of IPC programmes at the national and acute health care facility level for more information (http://www.who.int/infection-prevention/publications/core-components/en/ | □ Tidak | 0 |
|  | □ Ya, tanpa tujuan yang jelas | 5 |
|  | □ Ya, dengan tujuan yang jelas | 10 |
| **2. Apakah program PPI tersebut didukung oleh komite/tim PPI yang terdiri dari IPCN atau IPCD yang telah mengikuti pelatihan bersertifikat?**  Pilih satu jawaban | □ Tidak | 0 |
|  | □ Bukan sebuah tim, tetapi seorang fokal PPI | 5 |
|  | □ Ya | 10 |
| **3. Apakah tim PPI tersebut memiliki setidaknya satu ahli PPI purna waktu atau setara (perawat atau dokter yang 100% bekerja di PPI)?**  Pilih satu jawaban | □ Tidak ada ahli PPI yang tersedia | 0 |
|  | □ Tidak, *hanya* terdapat ahli PPI paruh waktu | 2,5 |
|  | □ Ya, satu per > 250 ranjang | 5 |
|  | □ Ya, satu per ≤ 250 ranjang | 10 |
| **4. Apakah tim PPI atau penanggung jawab PPI memiliki waktu khusus yang didedikasikan untuk kegiatan PPI?** | □ Tidak | 0 |
|  | □ Ya | 10 |
| **5. Apakah tim PPI tersebut termasuk dokter dan perawat?** | □ Tidak | 0 |
|  | □ Ya | 10 |
| **6. Apakah ada komite PPI* yang secara aktif mendukung tim PPI?**  *Komite PPI terdiri dari anggota yang berasal dari berbagai disiplin ilmu dan pemangku kepentingan di rumah sakit, yang berkontribusi dan memberikan masukan kepada tim PPI. Tim PPI merupakan IPCN atau ahli PPI yang berdedikasi yang bertanggung jawab atas program PPI di rumah sakit tersebut. | □ Tidak | 0 |
|  | □ Ya | 10 |
| **7. Apakah ada dari kelompok berikut yang terwakili/termasuk dalam komite PPI?** | | |
| Manajemen/Pimpinan Rumah Sakit (misalnya, direktur administrasi, kepala eksekutif [CEO], direktur medis) | □ Tidak | 0 |
|  | □ Ya | 5 |
| Dokter spesialis/klinisi dan perawat | □ Tidak | 0 |
|  | □ Ya | 2,5 |
| Bagian umum (misalnya, kesling dan *biosafety*) | □ Tidak | 0 |
|  | □ Ya | 2,5 |
| **8. Apakah Anda memiliki tujuan PPI yang jelas (yang terdapat pada area kritis spesifik)?**  Pilih satu jawaban | □ Tidak | 0 |
|  | □ Ya, hanya tujuan PPI | 2,5 |
|  | □ Ya, Tujuan PPI dan indikator hasil yang dapat diukur (yaitu tindakan yang memadai untuk improvement) | 5 |
|  | □ Ya, Tujuan PPI indikator hasil yang dapat diukur, dan target-target di masa depan | 10 |
| **9. Apakah manajemen/pimpinan rumah sakit menunjukkan komitmen dan dukungan yang jelas untuk program PPI:** | | |
| Dengan anggaran yang dialokasikan khusus untuk program PPI (yaitu mencakup kegiatan PPI, termasuk gaji)? | □ Tidak | 0 |
|  | □ Ya | 5 |
| Dengan dukungan nyata yang tertuang dalam tujuan dan indikator PPI di dalam fasilitas (misalnya, pada pertemuan tingkat eksekutif, putaran eksekutif, partisipasi dalam pertemuan morbiditas dan mortalitas)? | □ Tidak | 0 |
|  | □ Ya | 5 |
| **10. Apakah fasilitas Anda memiliki dukungan laboratorium mikrobiologi (baik di dalam atau di luar lokasi) untuk penggunaan rutin sehari-hari?**  Pilih satu jawaban | □ Tidak | 0 |
|  | □ Ya, tetapi tidak memberikan hasil dengan andal (tepat waktu dan berkualitas memadai) | 5 |
|  | □ Ya, dan memberikan hasil dengan andal (tepat waktu dan berkualitas memadai) | 10 |
| **Skor Subtotal** /100 | | |

**Komponen Inti 2: Pedoman Pencegahan dan Pengendalian Infeksi (PPI)**

| **Pertanyaan** | **Jawaban** | **Skor** |
| --- | --- | --- |
| **1. Apakah fasilitas Anda memiliki tenaga kesehatan/spesialis yang ahli dalam PPI dan/atau penyakit infeksi untuk mengembangkan atau mengadaptasi pedoman?** | □ Tidak | 0 |
|  | □ Ya | 7,5 |
| **2. Apakah fasilitas Anda menyediakan pedoman untuk:** | | |
| Kewaspadaan standar? | □ Tidak | 0 |
|  | □ Ya | 2,5 |
| Kebersihan tangan? | □ Tidak | 0 |
|  | □ Ya | 2,5 |
| Kewaspadaan berdasarkan transmisi?*  *Kewaspadaan berbasis penularan digunakan sebagai tambahan Kewaspadaan Standar untuk pasien yang mungkin terinfeksi atau terkolonisasi dengan agen infeksius tertentu yang memerlukan kewaspadaan tambahan untuk mencegah penularan infeksi. Kewaspadaan didasarkan pada rute penularan patogen tertentu (misalnya, kontak versus droplets/tetesan). Informasi lebih lanjut dapat ditemukan di Pusat Pengendalian dan Pencegahan Penyakit Amerika Serikat untuk Tindakan Pencegahan Isolasi (https://www.cdc.gov/infectioncontrol/pdf/guidelines/isolation-guidelines.pdf | □ Tidak | 0 |
|  | □ Ya | 2,5 |
| Manajemen dan kesiapsiagaan wabah? | □ Tidak | 0 |
|  | □ Ya | 2,5 |
| Pencegahan infeksi luka operasi?*  * Jika tidak ada intervensi bedah yang dilakukan di fasilitas Anda, pilih jawaban “Ya”. | □ Tidak | 0 |
|  | □ Ya | 2,5 |
| Pencegahan infeksi aliran darah karena pemasangan kateter pembuluh darah? | □ Tidak | 0 |
|  | □ Ya | 2,5 |
| Pencegahan pneumonia yang didapat di rumah sakit/*hospital-acquired pneumonia* ([HAP]; semua jenis HAP, termasuk (tetapi tidak secara eksklusif), pneumonia dikarenakan pemasangan ventilator)? | □ Tidak | 0 |
|  | □ Ya | 2,5 |
| Pencegahan infeksi saluran kemih dikarenakan pemasangan kateter urin? | □ Tidak | 0 |
|  | □ Ya | 2,5 |
| Pencegahan penularan oleh patogen resisten obat ganda/*multidrug resistant* (MDR)? | □ Tidak | 0 |
|  | □ Ya | 2,5 |
| Disinfeksi dan sterilisasi? | □ Tidak | 0 |
|  | □ Ya | 2,5 |
| Perlindungan dan keselamatan tenaga kesehatan?*  *Termasuk aspek peningkatan kondisi kerja, deteksi penyakit akibat kerja, pengawasan kesehatan pekerja, skrining pra-kerja dan vaksinasi. | □ Tidak | 0 |
|  | □ Ya | 2,5 |
| Penyuntikan yang aman? | □ Tidak | 0 |
|  | □ Ya | 2,5 |
| Manajemen limbah? | □ Tidak | 0 |
|  | □ Ya | 2,5 |
| Pedoman/kebijakan/SPO untuk penatagunaan antibiotik (*antibiotic stewardship*)?*  https://yankes.kemkes.go.id/unduhan/fileunduhan_1658480966_921055.pdf | □ Tidak | 0 |
|  | □ Ya | 2,5 |
| **3. Apakah pedoman di fasilitas Anda konsisten dengan pedoman nasional/internasional (jika ada)?** | □ Tidak | 0 |
|  | □ Ya | 10 |
| **4. Apakah penerapan pedoman ini disesuaikan^*^ dengan kebutuhan dan sumber daya yang ada dengan tetap mempertahankan standar PPI?**  *Komite atau Tim PPI dengan hati-hati meninjau pedoman untuk memprioritaskan aktivitas sesuai dengan kebutuhan dan sumber daya dengan tetap mempertahankan standar utama PPI | □ Tidak | 0 |
|  | □ Ya | 10 |
| **5. Apakah tenaga kesehatan pemberi pelayanan (selain anggota PPI) terlibat dalam perencanaan dan pelaksanaan penerapan pedoman PPI?** | □ Tidak | 0 |
|  | □ Ya | 10 |
| **6. Apakah pemangku kepentingan yang berkaitan (misalnya, komite medik, komite keperawatan, manajer rumah sakit, manajemen mutu) selain anggota PPI, terlibat dalam pengembangan dan adaptasi pedoman PPI?** | □ Tidak | 0 |
|  | □ Ya | 7,5 |
| **7. Apakah tenaga kesehatan menerima pelatihan khusus terkait dengan pedoman PPI yang dipebarui (*update*) yang ada di fasilitas tsb?** | □ Tidak | 0 |
|  | □ Ya | 10 |
| **8. Apakah Anda secara teratur memantau penerapan beberapa item dalam pedoman PPI di fasilitas Anda?** | □ Tidak | 0 |
|  | □ Ya | 10 |
| **Skor Subtotal** /100 | | |

**Komponen Inti 3: Pendidikan dan Pelatihan Pencegahan dan Pengendalian Infeksi (PPI)**

| **Pertanyaan** | **Jawaban** | **Skor** |
| --- | --- | --- |
| **1. Apakah ada personel dengan keahlian PPI (di PPI dan/atau penyakit menular) yang dapat memberikan pelatihan PPI?** | □ Tidak | 0 |
|  | □ Ya | 10 |
| **2. Apakah ada personel non-PPI tambahan dengan keterampilan yang memadai untuk menjadi pelatih dan mentor (misalnya dokter dan *IPCLN*, agen PPI)?**  Pilih satu jawaban | □ Tidak | 0 |
|  | □ Ya | 10 |
| **3. Seberapa sering tenaga kesehatan mendapatkan pelatihan mengenai PPI di rumah sakit Anda?**  Pilih satu jawaban | □ Tidak pernah atau jarang | 0 |
|  | □ Orientasi pegawai baru *hanya* untuk pekerja kesehatan | 5 |
|  | □ Orientasi pegawai baru dan pelatihan PPI teratur (setidaknya tahunan) untuk pekerja kesehatan ditawarkan, tetapi tidak wajib | 10 |
|  | □ Orientasi pegawai baru dan pelatihan PPI teratur (setidaknya tahunan) wajib untuk semua pekerja kesehatan | 15 |
| **4. Seberapa sering petugas kebersihan dan personel lain yang terlibat langsung dalam perawatan pasien menerima pelatihan tentang PPI di fasilitas Anda?**  Pilih satu jawaban | □ Tidak pernah atau jarang | 0 |
|  | □ Orientasi pegawai baru *hanya* untuk personel lain | 5 |
|  | □ Orientasi pegawai baru dan pelatihan teratur (setidaknya tahunan) untuk personel lain ditawarkan, tetapi tidak wajib | 10 |
|  | □ Orientasi pegawai baru dan pelatihan PPI teratur (setidaknya tahunan) wajib untuk personel lain | 15 |
| **5. Apakah staf administrasi dan manajerial menerima pelatihan umum tentang PPI di fasilitas Anda?**  Pilih satu jawaban | □ Tidak | 0 |
|  | □ Ya | 5 |
| **6. Bagaimana tenaga kesehatan dan personel lain dilatih?**  Pilih satu jawaban | □ Tidak ada pelatihan tersedia | 0 |
|  | □ Menggunakan informasi tertulis dan/tau instruksi lisan dan/atau e-learning saja | 5 |
|  | □ Termasuk sesi pelatihan interaktif tambahan (misalnya, simulasi dan/atau pelatihan di samping tempat tidur) | 10 |
| **7. Apakah ada evaluasi berkala terhadap keefektifan program pelatihan (misalnya, audit kebersihan tangan, pemeriksaan mengenai pengetahuan lainnya)?**  Pilih satu jawaban | □ Tidak | 0 |
|  | □ Ya, tapi tidak teratur | 5 |
|  | □ Ya, teratur (setidaknya tahunan) | 10 |
| **8. Apakah pelatihan PPI terintegrasi dengan pelatihan praktik klinis dan spesialisasi lain (misalnya, pelatihan ahli bedah memasukkan unsur PPI)?**  Pilih satu jawaban | □ Tidak | 0 |
|  | □ Ya, dalam beberapa disiplin ilmu | 5 |
|  | □ Ya, dalam semua disiplin ilmu | 10 |
| **9. Apakah ada pelatihan PPI spesifik untuk pasien atau anggota keluarga yang bertujuan untuk meminimalisir potensi infeksi yang berkaitan dengan kesehatan (misalnya, pasien dengan imunosupresi, pasien dengan perangkat invasif, pasien dengan infeksi infeksi resistensi obat ganda/*multidrug resistant*)?** | □ Tidak | 0 |
|  | □ Ya | 5 |
| **10. Apakah staf PPI mendapatkan pelatihan untuk meningkatkan dan mengembangkan keahliannya dalam bidang PPI (misalnya, dengan menghadiri konferensi, pelatihan)?** | □ Tidak | 0 |
|  | □ Ya | 10 |
| **Skor Subtotal**  /100 | | |

**Komponen Inti 4: Surveilans Infeksi Nosokomial**

| **Pertanyaan** | **Jawaban** | **Skor** |
| --- | --- | --- |
| **Organisasi Surveilans** | | |
| **1. Apakah surveilans termasuk ke dalam salah satu program PPI Anda?** | □ Tidak | 0 |
|  | □ Ya | 5 |
| **2. Apakah Anda memiliki personel yang bertanggung jawab atas aktivitas surveilans?** | □ Tidak | 0 |
|  | □ Ya | 5 |
| **3. Apakah para ahli bertanggung jawab untuk kegiatan surveilans telah dilatih tentang epidemiologi dasar, surveilans dan PPI (keahlian untuk mengawasi metode surveilans, manajemen data dan interpretasi)?** | □ Tidak | 0 |
|  | □ Ya | 5 |
| **4. Apakah Anda memiliki dukungan informatika/IT untuk melakukan surveilans (misalnya, peralatan, teknologi seluler, catatan kesehatan elektronik)?** | □ Tidak | 0 |
|  | □ Ya | 5 |
| **Prioritas surveilans – ditentukan menurut ruang lingkup perawatan** | | |
| **5. Apakah Anda menentukan skala prioritas terhadap HAI yang akan menjadi sasaran surveilans menurut kondisi di rumah sakit Anda (yaitu, mengidentifikasi yang merupakan penyebab utama morbiditas dan mortalitas di fasilitas)?***  *Latihan pembuatan prioritas harus dilakukan untuk menentukan HAI mana yang akan ditargetkan untuk surveilans sesuai dengan konteks lokal (misalnya, area dan / atau pasien paling berisiko) sesuai dengan sumber daya yang tersedia (lihat Interim practical manual yang mendukung implementasi Panduan Komponen Program Pencegahan dan Pengendalian Infeksi di http://www.who.int/infection-prevent/tools/core-components/en/, diakses 3 Mei 2018) | □ Tidak | 0 |
|  | □ Ya | 5 |
| **6. Pada fasilitas Anda, apakah surveilans bisa dilakukan untuk:** | | |
| Infeksi daerah operasi?*  *Jika tidak ada intervensi bedah yang dilakukan di fasilitas Anda, pilih jawaban "Ya" | □ Tidak | 0 |
|  | □ Ya | 2,5 |
| Infeksi akibat pemakaian alat kesehatan (misalnya, infeksi saluran kemih akibat pemasangan kateter, infeksi aliran darah akibat pemasangan kateter sentral, infeksi aliran darah akibat pemasangan kateter perifer, pneumonia akibat pemasangan ventilator)? | □ Tidak | 0 |
|  | □ Ya | 2,5 |
| Infeksi yang didiagnosa secara klinis (misalnya, definisi hanya berdasarkan pada tanda atau gejala klinis tanpa adanya pengujian mikrobiologis)? | □ Tidak | 0 |
|  | □ Ya | 2,5 |
| Kolonisasi atau infeksi yang disebabkan oleh kuman yang resistensi beberapa antimikroba* berdasarkan keadaan di rumah sakit Anda?  *Multidrug resistant: Tidak rentan terhadap setidaknya satu agen dalam tiga atau lebih kategori antimikroba; | □ Tidak | 0 |
|  | □ Ya | 2,5 |
| Penyakit infeksi terbanyak yang menjadi prioritas di daerah Anda (misalnya, norovirus, influenza, tuberculosis (TB), sindrom pernapasan akut berat/*severe acute respiratory syndrome* (SARS), Ebola, demam Lassa)? | □ Tidak | 0 |
|  | □ Ya | 2,5 |
| Infeksi pada populasi yang rentan (misalnya, neonatus, instalasi rawat intensif/*Intensive Care Unit* (ICU), kelainan imun, pasien luka bakar)?*  ^*^ Jika populasi pasien yang rentan tidak dirawat di fasilitas Anda, pilih jawaban “Ya”. | □ Tidak | 0 |
|  | □ Ya | 2,5 |
| Infeksi yang dapat terjadi pada petugas kesehatan di klinik, laboratorium, atau pengaturan lain (misalnya, hepatitis B atau C, *human immunodeficiency virus* (HIV, influenza)? | □ Tidak | 0 |
|  | □ Ya | 2,5 |
| **7. Apakah Anda mengevaluasi secara berkala surveilans Anda agar sejalan dengan kebutuhan saat ini dan prioritas di rumah sakit Anda?** | □ Tidak | 0 |
|  | □ Ya | 5 |
| **Metode Surveilans** | | |
| **8. Apakah Anda menggunakan definisi kasus surveilans terstandar (numerator (pembilang) dan denumerator (penyebut)) sesuai definisi internasional (misalnya CDC NHSN/ECDC) atau jika diadaptasi melalui proses adaptasi berbasis bukti dan konsultasi ahli?** | □ Tidak | 0 |
|  | □ Ya | 5 |
| **9. Apakah Anda menggunakan metode pengumpulan data sesuai standar (misalnya, surveilans prospektif dan aktif) sesuai dengan protocol surveilans internasional (misalnya. CDC NHSN/ECDC)* atau jika diadaptasi, melalui proses adaptasi berbasis bukti dan konsultasi ahli?**  *[15 United States Centers for Disease Control and Prevention (CDC) National Healthcare Safety Network (NHSN) (https://www.cdc.gov/nhsn/index.html, diakses 13 April 2018); European Center for Disease Prevention and Control (ECDC) (https://ecdc.europa.eu/en/about-us/partnerships-and-networks/disease-and-laboratory-networks/hai-net, diakses 13 April 2018).](file:////Users/indri/Desktop/15%20United%20States%20Centers%20for%20Disease%20Control%20and%20Prevention%20(CDC)%20National%20Healthcare%20Safety%20Network%20(NHSN)%20(https:/www.cdc.gov/nhsn/index.html,%20diakses%2013%20April%202018);European%20Center%20for%20Disease%20Prevention%20and%20Control%20(ECDC)%20(https:/ecdc.europa.eu/en/about-us/partnerships-and-networks/disease-and-laboratory-networks/hai-net,%20diakses%2013%20April%202018).) | □ Tidak | 0 |
|  | □ Ya | 5 |
| **10. Apakah Anda mengecek kualitas data secara teratur (misalnya, penilaian formulir laporan kasus, tinjauan hasil mikrobiologi, penentuan penyakit, dll)?** | □ Tidak | 0 |
|  | □ Ya | 5 |
| **11. Apakah Anda memiliki kapasitas laboratorium dan mikrobiologi yang memadai untuk mendukung surveilans?**  Pilih satu jawaban | □ Tidak | 0 |
|  | □ Ya, dapat membedakan strain gram positif/negatif tetapi tidak dapat mengidentifikasi patogen | 2,5 |
|  | □ Ya, dapat dapat mengidentifikasi patogen dengan terpercaya (misalnya, mengisolasi identifikasi) pada waktu yang tepat | 5 |
|  | □ Ya, dapat diandalkan untuk mengidentifikasi patogen dan pola resistensi obat antimikroba (yaitu kerentanan) secara tepat waktu | 10 |
| **Analisis informasi dan diseminasi/penggunaan data, jejaring, dan tata kelola** | | |
| **12. Apakah data surveilans digunakan untuk membuat perencanaan perbaikan PPI di rumah sakit Anda?** | □ Tidak | 0 |
|  | □ Ya | 5 |
| **13. Apakah Anda menganalisa resistensi obat antimicrobial secara teratur (misalnya, tiga bulan sekali, enam bulan sekali, setahun sekali)?** | □ Tidak | 0 |
|  | □ Ya | 5 |
| **14. Apakah Anda secara teratur (misalnya, tiga bulan sekali/enam bulan sekali/setahun sekali) memberikan umpan balik informasi surveilans terkini** | | |
| Tenaga kesehatan pemberi pelayanan langsung (dokter/perawat)? | □ Tidak | 0 |
|  | □ Ya | 2,5 |
| komite Medik/kepala departemen | □ Tidak | 0 |
|  | □ Ya | 2,5 |
| Komite PPI | □ Tidak | 0 |
|  | □ Ya | 2,5 |
| Pimpinan atau pihak manajemen non-klinik (bagian keuangan, administrasi)? | □ Tidak | 0 |
|  | □ Ya | 2,5 |
| **15. Bagaimana Anda memberikan umpan balik terbaru mengenai informasi surveilans? (setidaknya setahun sekali)**  Pilih satu jawaban | □ Tidak ada umpan balik | 0 |
|  | □ Dengan informasi oral/tertulis | 2,5 |
|  | □ Dengan presentasi dan penemuan solusi berorientasi masalah interaktif | 7,5 |
| **Skor Subtotal** /100 | | |

**Komponen Inti 5: Strategi Multimodal untuk Implementasi Intervensi Pencegahan dan Pengendalian Infeksi (PPI)**

| **Pertanyaan** | **Jawaban** | **Skor** |
| --- | --- | --- |
| 1. Apakah Anda menggunakan strategi multimodal* untuk mengimplementasikan intervensi PPI?  *Lihat definisi di http://www.who.int/infection-prevention/publications/ipc-cc-mis.pdf?ua=1, diakses 13 April 2018. Penggunaan strategi multimodal di PPI telah terbukti sebagai pendekatan berbasis terbaik untuk mencapai sistem yang berkelanjutan dan perubahan perilaku untuk implementasi intervensi PPI. Strategi multimodal: ≥3 komponen diimplementasikan secara terintegrasi untuk mencapai peningkatan hasil dan mengubah perilaku (misalnya, praktik kebersihan tangan). dapat mencakup (i) perubahan sistem (misalnya, menyediakan infrastruktur, persediaan, dan sumber daya manusia yang diperlukan), (ii) pendidikan dan pelatihan pekerja perawatan kesehatan dan pemain kunci (misalnya, manajer), (iii) memantau infrastruktur, praktik, proses, hasil dan memberikan umpan balik data; (iv) pengingat di tempat kerja / komunikasi; dan (v) perubahan budaya dalam pembentukan atau penguatan iklim keselamatan. Hal ini juga mencakup alat, seperti checklist dan bundel, dikembangkan oleh tim multidisiplin yang mempertimbangkan kondisi lokal. Kelima komponen tsb harus dipertimbangkan berdasarkan konteks dan situasi lokal yang diinformasikan oleh penilaian secara berkala. Pelajaran dari bidang ilmu implementasi menunjukkan bahwa menargetkan hanya satu dari lima elemen ini (yaitu, menggunakan strategi "unimodal") lebih cenderung menghasilkan perbaikan yang berumur singkat dan tidak berkelanjutan. Untuk informasi lebih lanjut, silakan lihat: http://www.who.int/infection-prevention/publications/ipc-cc-mis.pdf?ua=1, diakses 13 April 2018 dan manual praktis Interim yang mendukung implementasi Pedoman WHO tentang Komponen Inti Program Pencegahan dan Pengendalian Infeksi di http://www.who.int/infection-prevention/tools/core-components/en/, diakses 3 April 2018. | □ Tidak | 0 |
|  | □ Ya | 15 |
| **2. Apakah strategi multimodal Anda mencakup salah satu atau semua elemen berikut:**  Pilih satu jawaban (yang paling akurat) per elemen | **Perubahan Sistem** | 0 |
|  | □ Elemen tidak termasuk dalam strategi multimodal | 0 |
|  | □ Ada intervensi untuk memastikan infrastruktur yang diperlukan dan ketersediaan pasokan yang berkelanjutan | 5 |
|  | □ Intervensi untuk memastikan infrastruktur yang diperlukan dan ketersediaan pasokan yang berkelanjutan dan mengatasi persoalan ergonomi^17^ dan aksesibilitas, seperti penempatan terbaik dari set kateter vena sentral dan baki | 10 |
|  | **Pendidikan dan Pelatihan** | |
|  | □ Elemen tidak termasuk dalam strategi multimodal | 0 |
|  | □ Informasi tertulis dan/atau instruksi lisan dan/atau e-learning saja | 5 |
|  | □ Sesi pelatihan interaktif tambahan (termasuk simulasi dan/atau pelatihan di samping tempat tidur) | 10 |
|  | **Monitoring dan umpan balik** | |
|  | □ Elemen tidak termasuk dalam strategi multimodal | 0 |
|  | □ Memantau kepatuhan dengan indikator proses atau hasilnya (misalnya, audit kebersihan tangan atau praktik kateter) | 5 |
|  | □ Memantau kepatuhan dan memberikan umpan balik tepat wakktu dari hasil pemantauan kepada pekerja perawatan kesehatan dan pemain kunci | 10 |
|  | **Komunikasi dan Pengingat** | |
|  | □ Elemen tidak termasuk dalam strategi multimodal | 0 |
|  | □ Pengingat, poster, atau alat advokasi / peningkatan kesadaran lainnya untuk mempromosikan intervensi | 5 |
|  | □ Metode / inisiatif tambahan untuk meningkatkan komunikasi tim lintas unit dan disiplin ilmu (misalnya, dengan mengadakan konferensi kasus reguler dan putaran umpan balik) | 10 |
|  | **Perubahan iklim dan budaya keselamatan** | |
|  | □ Elemen tidak termasuk dalam strategi multimodal | 0 |
|  | □ Manajer/pemimpin menunjukkan dukungan yang terlihat dan bertindak sebagai champion dan role model, mempromosikan pendekatan adaptif^18^ dan memperkuat budaya yang mendukung IPC, keselamatan dan kualitas pasien | 5 |
|  | □ Selain itu, tim dan individu diberdayakan sehingga mereka merasa memiliki intervensi (misalnya, dengan putaran umpan balik partisipatif) | 10 |
| **3. Apakah tim multidisiplin digunakan untuk menerapkan strategi multimodal PPI?** | □ Tidak | 0 |
|  | □ Ya | 15 |
| **4. Apakah Anda secara teratur menghubungkan ke kolega dari peningkatan kualitas dan keselamatan pasien untuk mengembangkan dan mempromosikan strategi multimodal PPI?** | □ Tidak | 0 |
|  | □ Ya | 10 |
| **5. Apakah strategi ini mencakup bundle* atau checklist?**  *Pendekatan adaptif mempertimbangkan kompleksitas perilaku, organisasi dan budaya dalam sistem perawatan kesehatan. | □ Tidak | 0 |
|  | □ Ya | 10 |
| **Skor Subtotal** /100 | | |

**Komponen Inti 6: Pemantauan/Audit Praktik PPI dan Umpan Balik**

| **Pertanyaan** | **Jawaban** | **Skor** |
| --- | --- | --- |
| **1. Apakah Anda memiliki personel terlatih yang bertanggung jawab untuk memantau/ mengaudit pelaksanaan dan umpan balik PPI?** | □ Tidak | 0 |
|  | □ Ya | 10 |
| **2. Apakah Anda memiliki rencana pemantauan yang ditetapkan dengan baik dengan tujuan, target, dan aktivitas yang jelas (termasuk alat untuk mengumpulkan data secara sistematis)?** | □ Tidak | 0 |
|  | □ Ya | 7,5 |
| **3. Kegiatan dan indikator mana yang Anda pantau di fasilitas Anda?**  Centang semua yang sesuai | □ Tidak ada | 0 |
|  | □ Kepatuhan kebersihan tangan (menggunakan alat observasi kebersihan tangan WHO^*^ atau yang setara)  * Alat pemantauan kebersihan tangan dan umpan balik WHO dapat ditemukan di sini: http://www.who.int/infection-prevention/tools/hand-hygiene/evaluation_feedback/en/, diakses pada 18 April 2018. | 5 |
|  | □ Pemasangan dan/atau perawatan kateter intravascular | 5 |
|  | □ Penggantian balutan luka | 5 |
|  | □ Kewaspadaan berdasarkan transmisi dan isolasi untuk mencegah penyebaran mikroorganisme yang resisten multi obat (*multidrug resistant organism*) | 5 |
|  | □ Pembersihan lingkungan bangsal | 5 |
|  | □ Desinfeksi dan sterilisasi peralatan/instrumen medis | 5 |
|  | □ Konsumsi/penggunaan handrub atau sabun berbahan dasar alcohol | 5 |
|  | □ Konsumsi/penggunaan agen antimikroba | 5 |
|  | □ Penanganan limbah | 5 |
| **4. Seberapa sering WHO Hand Hygiene Self-Assessment Framework Survey* dilakukan?**  Pilih 1 jawaban  * WHO Hand Hygiene Self-Assessment Framework dapat ditemukan disini: http://www.who.int/gpsc/country_work/hhsa_framework_October_2010.pdf?ua=1, diakses pada 18 April 2018. | □ Tidak pernah | 0 |
|  | □ Secara periodik, tetapi tidak ada jadwal reguler | 2,5 |
|  | □ Setidaknya setahun sekali | 5 |
| **5. Apakah Anda memberi umpan balik pada laporan audit (misalnya, umpan balik pada data kepatuhan kebersihan tangan atau proses lain) tentang keadaan aktivitas / kinerja PPI?**  Centang semua yang sesuai | □ Tidak ada pelaporan | 0 |
|  | □ Ya, di dalam tim PPI | 2,5 |
|  | □ Ya, ke pemimpin dan manajer departemen di area yang diaudit | 2,5 |
|  | □ Ya, ke pekerja perawatan kesehatan garis depan | 2,5 |
|  | □ Ya, ke komite PPI atau komite perawatan kualitas atau yang setara | 2,5 |
|  | □ Ya, ke manajemen rumah sakit dan administrasi senior | 2,5 |
| **6. Apakah pelaporan data monitoring dilakukan secara rutin (setidaknya setahun sekali)?** | □ Tidak | 0 |
|  | □ Ya | 10 |
| **7. Apakah pemantauan dan umpan balik kegiatan dan indikator PPI dilakukan dalam budaya kelembagaan: “tidak saling menyalahkan" yang bertujuan untuk perbaikan dan perubahan perilaku di rumah sakit Anda?** | □ Tidak | 0 |
|  | □ Ya | 5 |
| **8. Apakah Anda menilai faktor budaya keselamatan di fasilitas Anda (misalnya, dengan menggunakan survei lain seperti HSOPSC, SAQ, PSCHO, HSC22*)**  *HSOPSC: Hospital survey on patient safety culture; SAQ: Safety attitudes questionnaire, PSCHO: Patient safety climate in healthcare organizations; HSC: Hospital safety climate scale. Ringkasan survei ini dapat ditemukan di: Colla JB, et al. Measuring patient safety climate: a review of survey. Qual Saf Health Care. 2005;14(5):364-6 (https://www.ncbi.nlm.nih.gov/pubmed/16195571, diakses pada 13 April 2018). | □ Tidak | 0 |
|  | □ Ya | 5 |
| **Skor Subtotal** /100 | | |

**Komponen Inti 7: Beban Kerja, Penempatan Staf dan Hunian Tempat Tidur***

* Khususnya untuk pertanyaan-pertanyaan ini, tim PPI mungkin perlu berkonsultasi dengan tim terkait lainnya di fasilitas untuk dapat menjawab pertanyaan yang sesuai.

| **Pertanyaan** | **Jawaban** | **Skor** |
| --- | --- | --- |
| **Penempatan Staff** | | |
| **1. Apakah anda melalukan analisis di fasilitas Anda sesuai dengan beban kerja pasien dengan menggunakan standar nasional atau alat penilaian kebutuhan kepegawaian standar seperti indikator beban kerja WHO mengenai metode kebutuhan staf/ WHO *Workload indicators of staffing need**?**  * Indikator Beban Kerja WHO tentang metode kebutuhan staf memberi manajer kesehatan cara sistematis untuk menentukan berapa banyak petugas kesehatan dari jenis tertentu yang diperlukan untuk mengatasi beban kerja fasilitas kesehatan tertentu dan membantu pengambilan keputusan. (http://www.who.int/hrh/resources/wisn_user_manual/en/, diakses pada 13 April 2018). | □ Tidak | 0 |
|  | □ Ya | 5 |
| **2. Apakah rasio yang disepakati (yaitu, WHO atau nasional) antara petugas kesehatan dan pasien dipertahankan di rumah sakit Anda?**  Pilih satu jawaban  * Memperhatikan semua pekerja perawatan kesehatan yang terlibat dalam pemberian layanan dan perawatan pasien, termasuk staf klinis (dokter, perawat, dokter gigi, asisten medis, dll.), Teknisi laboratorium, dan pekerja perawatan kesehatan lainnya (misalnya, pembersih). | □ Tidak | 0 |
|  | □ Ya, untuk staf di kurang dari 50% unit | 5 |
|  | □ Ya, untuk staf di lebih dari 50% unit | 10 |
|  | □ Ya, untuk semua petugas kesehatan di fasilitas tersebut | 15 |
| **3. Apakah sistem di fasilitas Anda untuk bertindak berdasarkan hasil dari penilaian kebutuhan staf ketika tingkat staf dianggap terlalu rendah?** | □ Tidak | 0 |
|  | □ Ya | 10 |
| **Hunian Tempat Tidur** | | |
| **4. Apakah desain bangsal di fasilitas Anda sesuai dengan standar internasional* mengenai kapasitas tempat tidur?**  Pilih satu jawaban  * Standar kesehatan lingkungan esensial WHO dalam panduan perawatan kesehatan memberikan panduan tentang standar yang diperlukan untuk perawatan kesehatan di negara dengan sumber daya menengah dan rendah. Panduan ini telah ditulis untuk digunakan oleh manajer dan perencana kesehatan, arsitek, perencana kota, staf air dan sanitasi, staf klinis dan perawat, penjaga dan penyedia layanan kesehatan lainnya, dan promotor kesehatan. (http://www.who.int/ water_sanitation_health/publications/ehs_hc/en/, diakses pada 13 April 2018). | □ Tidak | 0 |
|  | □ Ya, tapi hanya di departemen tertentu | 5 |
|  | □ Ya, di semua departemen (termasuk departemen darurat dan pediatri) | 15 |
| **5. Apakah hunian tempat tidur di rumah sakit anda satu pasien per tempat tidur?**  Pilih satu jawaban | □ Tidak | 0 |
|  | □ Ya, tapi hanya di departemen tertentu | 5 |
|  | □ Ya, di semua departemen (termasuk departemen darurat dan pediatri) | 15 |
| **6. Adakah pasien yang tempat tidurnyadiletakkan di koridor di luar ruang rawat di rumah sakit Anda (termasuk tempat tidur di unit gawat darurat)?**  Pilih satu jawaban | □ Ya, lebih sering dari 2x seminggu | 0 |
|  | □ Ya, tidak lebih sering dari 2x seminggu | 5 |
|  | □ Tidak | 15 |
| **7. Apakah jarak antara tempat tidur pasien memadai (yaitu > 1 meter) terjamin di fasilitas Anda?**  Pilih satu jawaban | □ Tidak | 0 |
|  | □ Ya, tapi hanya di departemen tertentu | 5 |
|  | □ Ya, di semua departemen (termasuk departemen darurat dan pediatri) | 15 |
| **8. Apakah ada sistem di fasilitas Anda untuk menilai dan merespons ketika kapasitas tempat tidur yang memadai terlampaui?**  Pilih satu jawaban | □ Tidak | 0 |
|  | □ Ya, ini adalah tanggung jawab kepala departemen | 5 |
|  | □ Ya, ini adalah tanggung jawab administrasi/manajemen rumah sakit | 15 |
| **Skor Subtotal** /100 | | |

**Komponen Inti 8: Lingkungan Buatan, Bahan dan Peralatan untuk PPI di Tingkat Fasilitas^*^**

* Komponen ini dapat dinilai secara lebih rinci menggunakan WHO Water and sanitation for health facility improvement tool (WASH FIT) (http://www.who.int/water_sanitation_health/publications/water-and- sanitation-for-health-facility-improvement-tool/en/, diakses April 2018). Khusus untuk pertanyaan-pertanyaan ini, tim PPI mungkin perlu berkonsultasi dengan tim terkait lainnya di fasilitas tersebut agar dapat menjawab pertanyaan-pertanyaan secara tepat dan akurat.

| **Pertanyaan** | **Jawaban** | **Skor** |
| --- | --- | --- |
| **Air** | | |
| **1. Apakah layanan air tersedia setiap saat dan dalam jumlah yang cukup untuk semua penggunaan (misalnya, mencuci tangan, minum, kebersihan diri, aktivitas medis, sterilisasi, dekontaminasi, pembersihan, dan cuci baju)?**  Pilih satu jawaban | □ Tidak, tersedia rata-rata <5 hari per minggu | 0 |
|  | □ Ya, tersedia rata-rata ≥ 5 hari per minggu atau setiap hari tetapi jumlahnya tidak mencukupi | 2,5 |
|  | □ Ya, setiap hari dan dalam jumlah yang cukup | 7,5 |
| **2. Apakah tempat air layak minum dapat diakses oleh staf, pasien, dan keluarga setiap saat dan di semua lokasi / bangsal?**  Pilih satu jawaban | □ Tidak, tidak tersedia | 0 |
|  | □ Terkadang, atau hanya di beberapa tempat atau tidak tersedia untuk semua pengguna | 2,5 |
|  | □ Ya, dapat diakses setiap saat dan untuk semua lingkungan/kelompok | 7,5 |
| **Fasilitas sanitasi dan kebersihan tangan** | | |
| **3. Apakah fasilitas cuci tangan yang berfungsi (yaitu larutan pembersih tangan berbasis alkohol atau sabun dan air dan handuk bersih sekali pakai) tersedia di semua titik perawatan?**  Pilih satu jawaban | □ Tidak, tidak ada | 0 |
|  | □ Ya, stasiun tersedia, tetapi persediaan tidak selalu ada | 2,5 |
|  | □ Ya, dengan persediaan selalu ada | 7,5 |
| **4. Pada fasilitas Anda, apakah ≥ 4 toilet^*^ tersedia untuk pengaturan rawat jalan atau ≥ 1 per 20 pengguna untuk pengaturan rawat inap?**  Pilih satu jawaban  * Fasilitas sanitasi yang lebih baik termasuk toilet siram ke dalam selokan yang dikelola atau tangki septik dan lubang rendam, jamban VIP, jamban jamban dengan toilet slab dan kompos. Agar dianggap dapat digunakan, toilet / kakus harus memiliki pintu yang tidak terkunci saat tidak digunakan (atau yang kuncinya tersedia kapan saja) dan dapat dikunci dari dalam selama digunakan. Tidak boleh ada lubang besar atau retakan atau kebocoran pada struktur toilet, lubang atau pit tidak boleh tersumbat, air harus tersedia untuk toilet siram / siram. Ini harus berada di dalam halaman fasilitas dan harus bersih seperti yang dicatat dengan tidak adanya limbah, kotoran dan kotoran yang terlihat serta serangga. | □ Jumlah toilet yang tersedia dan berfungsi kurang dari yang dibutuhkan | 0 |
|  | □ Jumlahnya mencukupi tetapi tidak semua berfungsi | 2,5 |
|  | □ Jumlahnya cukup dan berfungsi | 7,5 |
| **Power supply ventilasi, dan pembersihan** | | |
| **5. Di fasilitas perawatan kesehatan Anda, apakah energi / power supply yang cukup tersedia pada siang dan malam untuk semua penggunaan (misalnya, memompa dan merebus air, sterilisasi dan dekontaminasi, insinerasi atau teknologi pengobatan alternatif, perangkat medis elektronik, penerangan umum di area tempat dimana prosedur perawatan kesehatan diterapan untuk memastikan penyediaan perawatan kesehatan yang aman dan penerangan fasilitas toilet dan kamar mandi?)**  Pilih satu jawaban | □ Tidak | 0 |
|  | □ Ya, terkadang atau hanya di beberapa area yang disebutkan | 2,5 |
|  | □ Ya, selalu dan di semua area yang disebutkan | 5 |
| **6. Apakah ventilasi lingkungan (alami atau mekanis*) tersedia dan berfungsi dengan baik di area perawatan pasien?**  * Ventilasi alami: udara luar yang digerakkan oleh kekuatan alam (misalnya, angin) melalui bukaan yang dibuat khusus untuk bangunan, termasuk jendela, pintu, cerobong surya, menara angin, dan ventilator tetesan. Ventilasi mekanis: udara yang digerakkan oleh van mekanis yang dipasang langsung di jendela atau dinding atau di saluran udara untuk memasok udara ke, atau membuang udara dari, ruangan. Informasi lebih lanjut di: http://www.who.int/ water_sanitation_health/publications/natural_ventilation/en/, accessed 13 April 2018. | □ Tidak | 0 |
|  | □ Ya | 5 |
| **7. Untuk lantai dan permukaan lingkungan tempat kerja Anda, apakah ada catatan kebersihan yang dapat diakses, dan ditandatangani oleh pembersih setiap hari?**  Pilih satu jawaban | □ Tidak ada catatan lantai dan permukaan yang sedang dibersihkan | 0 |
|  | □ Rekaman ada, tetapi tidak diselesaikan dan ditandatangani setiap hari atau sudah using | 2,5 |
|  | □ Ya, rekaman diselesaikan dan ditandatangani setiap hari | 5 |
| **8. Apakah tersedia bahan yang layak dan terawat dengan baik untuk kebersihan (misalnya, deterjen, pel, ember, dll.)?**  Pilih satu jawaban | □ Tidak ada bahan yang tersedia | 0 |
|  | □ Ya, tersedia tapi tidak terawat dengan baik | 2,5 |
|  | □ Ya, tersedia dan terawat dengan baik | 5 |
| **Penempatan pasien dan alat pelindung diri (APD) dalam pengaturan perawatan kesehatan** | | |
| **9. Apakah Anda memiliki kamar pasien tersendiri atau ruangan untuk mengumpulkan/cohorting* pasien dengan patogen serupa jika jumlah ruang isolasi tidak mencukupi (misalnya, TB, campak, kolera, Ebola, SARS)?****  Pilih satu jawaban  * Strategi pengelompokan harus didasarkan pada penilaian risiko yang dilakukan oleh tim PPI. **Kondisi ventilasi tekanan negatif di ruang isolasi mungkin diperlukan untuk mencegah penularan beberapa organisme (misalnya, TB yang resistan terhadap beberapa obat). | □ Tidak | 0 |
|  | □ Tidak ada kamar *single* melainkan kamar yang sesuai untuk kelompok pasien yang tersedia | 2,5 |
|  | □ Ya, kamar *single* tersedia | 7,5 |
| **10. Apakah APD* tersedia setiap saat dan dalam jumlah yang mencukupi untuk seluruh tenaga kesehatan?**  Pilih satu jawaban  * Alat Pelindung Diri (APD): Sarung tangan medis non-steril dan steril bedah, masker bedah, kacamata pelindung atau pelindung wajah dan gaun pelindung dianggap sebagai APD penting. Respirator dan celemek juga harus tersedia dalam jumlah yang memadai di semua fasilitas untuk digunakan bila diperlukan. | □ Tidak | 0 |
|  | □ Ya, tetapi tidak terus menerus tersedia dalam jumlah yang cukup | 2,5 |
|  | □ Ya, tersedia terus menerus dalam jumlah yang cukup | 7,5 |
| **Pengelolaan limbah medis dan pembuangan limbah** | | |
| **11. Apakah Anda memiliki wadah pengumpulan limbahuntuk**  **limbah tidak menular (umum), limbah infeksius dan, limbah benda tajam yang berfungsi di dekat semua titik pembuangan limbah?**  Pilih satu jawaban | □ Tidak ada tempat sampah atau pembuangan benda tajam yang terpisah | 0 |
|  | □ Ada tempat sampah terpisah tetapi tutupnya hilang atau lebih dari ¾ penuh; hanya dua tempat sampah (bukan tiga); atau tempat sampah di beberapa titik tetapi tidak semua titik timbulan sampah | 2,5 |
|  | □ Iya | 5 |
| **12. Apakah pada lubang galian/tempat pembuangan sampah dipagari atau pengambilan sampah dari pemerintah kota tersedia untuk pembuangan non-infeksius (limbah tidak berbahaya/umum)?**  Pilih satu jawaban | □ Tidak ada lubang atau metode pembuangan lain yang digunakan | 0 |
|  | □ Terdapat lubang di fasilitas tetapi dimensinya tidak memadai; lubang/timbunan terlalu penuh atau tidak dipagari/kunci, atau pengambilan sampah kota yang tidak teratur | 2,5 |
|  | □ Iya | 5 |
| **13. Apakah insinerator atau teknologi pengolahan alternatif untuk pengolahan limbah benda tajam dan menular (misalnya, autoklaf) yang ada (baik ada di dalam atau di luar lokasi dan dioperasikan oleh layanan pengelolaan limbah berlisensi), berfungsi dengan kapasitas yang memadai?**  Pilih satu jawaban | □ Tidak, tidak ada | 0 |
|  | □ Ada, tapi tidak berfungsi | 1 |
|  | □ Ya | 5 |
| **14. Apakah sistem pengolahan air limbah (misalnya, septic tank diikuti dengan lubang drainase) tersedia (baik di dalam maupun di luar lokasi) dan berfungsi dengan baik?**  Pilih satu jawaban | □ Tidak, tidak ada | 0 |
|  | □ Iya, tapi tidak berfungsi dengan baik | 2,5 |
|  | □ Iya, dan berfungsi dengan baik | 5 |
| **Dekontaminasi dan sterilisasi** | | |
| **15.Apakah rumah sakit Anda menyediakan area dekontaminasi khusus dan / atau departemen pasokan steril (baik ada di dalam atau di luar lokasi dan dioperasikan oleh layanan manajemen dekontaminasi berlisensi) untuk dekontaminasi dan sterilisasi alat kesehatan dan barang / perlengkapan lainnya?**  Pilih satu jawaban | □ Tidak, tidak ada | 0 |
|  | □ Iya, tapi tidak berfungsi dengan baik | 2,5 |
|  | □ Iya, dan berfungsi dengan baik | 5 |
| **16. Apakah Anda memiliki peralatan yang steril dan didesinfeksi yang siap pakai?**  Pilih satu jawaban | □ Tidak, tersedia rata-rata <lima hari per minggu | 0 |
|  | □ Ya, tersedia rata-rata ≥ lima hari per minggu atau setiap hari, tetapi jumlahnya tidak mencukupi | 2,5 |
|  | □ Ya, tersedia setiap hari dan dalam jumlah yang cukup | 5 |
| **17. Apakah barang disposable tersedia jika dibutuhkan? (misalnya, alat pengaman injeksi, sarung tangan pemeriksaan)**  Pilih satu jawaban | □ Tidak, tidak tersedia | 0 |
|  | □ Iya, tetapi hanya tersedia kadang-kadang | 2,5 |
|  | □ Iya, tersedia secara berkelanjutan | 5 |
| **Skor Subtotal** /100 | | |

**Additional file 4. Additional questions**

| **No** | **Question** | **Answer** |
| --- | --- | --- |
| 1 | What is the name of your hospital? |  |
| 2 | What is the class of the hospital? | 1. A 2. B 3. C 4. D |
| 3 | Which region does your hospital belong to? | 1. Region 1 (Java. Sumatera and Bali) 2. Region 2 (Sulawesi, Kalimantan, and West Nusa Tenggara) 3. Region 3 (Maluku, Papua, and East Nusa Tenggara) |
| 4 | What is the ownership of your hospital? | 1. Private 2. State-owned enterprise 3. Government |
| 5 | Does your hospital accredit? | 1. Yes 2. No |
| 6 | What do you consider the top challenges for implementing IPC at this facility?  (Please rank them based on the priority from the highest to the lowest) | 1. AMR pattern/antibiogram 2. Surveillance of HAIs 3. Decolonization/disinfection/antiseptics 4. Changes in behaviors 5. Facilities **(**microbiology laboratory, incinerator, logistic support, solid treatment plant, internet and hand hygiene facilities) 6. Funding 7. Availability of IPCN full time 8. Dissemination 9. Training and education 10. Communication with management of the hospitals 11. Changes in organization |
| 7 | What would be your recommendations to improve IPC in this facility? | 1. Hospital level 2. National level |
| 8 | Please mention challenges in implementing IPC in your hospitals for CC, as follow: | 1. Education and training 2. Surveillance of HAIs 3. Multimodal strategies |

**Additional file 5. Questionnaire for the management of the hospitals (English and Bahasa Indonesia)**

**English version**

**Annex 1: Questionnaires used for semi‑structured interviews with health managers**

| **No** | **Question** | **Answer** | **Skip** |
| --- | --- | --- | --- |
| 1 | Does your facility have an IPC program? | 1. No 2. Yes |  |
| 2 | Does your facility have a budget specifically allocated to the IPC program (e.g., to address IPC materials, administrative support, staff )? | 1. No 2. Yes |  |
| 3 | Is there an IPC team with one or more IPC staff with dedicated time for IPC activities (full time healthcare workers?? | 1. No 2. Yes | SKIP to 5 |
| 4 | Does the IPC team have at least one full-time IPC specialist (a nurse or a doctor or an epidemiologist) working 100% on IPC? | 1. All IPC staff work part-time on IPC 2. 1 or more full-time IPC specialist for 100 beds and less 3. Less than 1 full-time IPC specialist for 100 beds and less |  |
| 5 | Do you have an established IPC Committee or an equivalent actively supporting the IPC team? | 1. No 2. Yes |  |
| 6 | When was the last time you led or participated in a meeting to discuss IPC related objectives, targets and challenges? | 1. Never 2. More than 3 months ago 3. Within the past 3 months |  |

**Translated version (Bahasa Indonesia)**

Target responden : Direktur Utama/Direktur Medis RS/Direktur Umum dan Operasional atau yang ditunjuk misalnya Kabid Yanmed **(kecuali komite/tim PPI)**

Nama responden :

Jabatan :

Pewawancara :

Pengentry data :

| **No** |  | **Pertanyaan** | | **Jawaban** | |
| --- | --- | --- | --- | --- | --- |
| M1 | Sudah berapa lama bapak/ibu menduduki posisi sekarang ini? | |  | |  |
| M2 | Apakah rumah sakit Anda memiliki program PPI? | | 1. Tidak 2. Ya | |  |
| M3 | Apakah rumah sakit Anda memiliki anggaran yang dialokasikan secara khusus untuk program PPI (misalnya, untuk menangani perihal PPI, dukungan administratif, staf)? | | 1. Tidak 2. Ya | |  |
| M4 | Apakah menurut anda, budget yang dialokasikan sudah mencukupi untuk pelaksanaan kegiatan PPI di rumah sakit anda? | | - - - 1. Tidak       2. Ya | |  |
| M5 | Apakah Komite atau Tim PPI atau penanggung jawab memiliki waktu yang didedikasikan untuk kegiatan PPI? | | 1. Tidak 2. Ya | |  |
| M6 | Apakah tim PPI tersebut memiliki setidaknya satu ahli PPI purna waktu atau setara (perawat atau dokter yang 100% bekerja di PPI)? Pilih satu jawaban | | 1. Semua staf PPI bekerja paruh waktu di PPI 2. 1 atau lebih spesialis PPI *full-time* untuk 100 atau dibawah 100 tempat tidur 3. dari 1 spesialis PPI penuh waktu untuk 100 atau dibawah 100 tempat tidur | |  |
| M7 | Apakah Anda memiliki Komite PPI yang telah dibentuk atau sejenisnya yang secara aktif mendukung tim PPI? | | 1. Tidak 2. Ya | |  |
| M8 | Kapan terakhir kali Anda memimpin atau berpartisipasi dalam rapat untuk membahas tujuan, target, dan tantangan terkait PPI? | | 1. Tidak Pernah 2. Lebih dari 3 bulan yang lalu 3. Dalam 3 bulan terakhir | |  |
| M9 | Apakah program PPI yang ada memiliki Indikator hasil yang jelas dan dapat diukur untuk rumah sakit tersebut? Pilih satu jawaban | | 1. Tidak 2. Ya, indikator hasil PPI dapat diukur (atau ukuran yang memadai untuk perbaikan) 3. Ya, indikator hasil PPI dapat diukur dan memiliki target untuki masa mendatang | |  |
| M10 | Apakah komite/tim PPI memberikan laporan hasil monitoring atau surveilans minimal satu tahun sekali? | | - - - 1. Tidak       2. Ya, hanya laporan tertulis tanpa pertemuan untuk memberikan penjelasan atau diskusi bersama       3. Ya, dengan pertemuan untuk memberikan penjelasan dan diskusi bersama | |  |
| M11 | Apakah menurut anda, jumlah kasus HAIs berdasarkan hasil surveilans sudah menggambarkan angka HAIs di rumah sakit anda yang sebenarnya? | | Tidak, lanjutkan ke pertanyaan selanjutnya?  Ya | |  |
| M12 | Jeaskan kenapa tidak sesuai | |  | |  |
| M13 | Tindakan apa yang dilakukan apabila angka HAIs di rumah sakit melampaui target yang ditetapkan | |  | |  |
| M14 | Apakah jumlah IPCN di rumah sakit anda sudah sesuai/ideal? | | - - - 1. Sudah       2. Belum, lanjutkan ke pertanyaan berikutnya | |  |
| M15 | Bagaimana RS mengantisipasi keadaan ini? | |  | |  |
| M16 | Apa rekomendasi Anda untuk meningkatkan PPI di rumah sakit ini | |  | |  |
| M17 | Apa rekomendasi Anda untuk meningkatkan PPI di tingkat nasional | |  | |  |

**Additional file 6. Questionnaire for IPC team/committee (English and Bahasa)**

**English version**

**PART 1**

| **No** | **Question** | **Answer** | **Skip** |
| --- | --- | --- | --- |
| 1 | Does your facility have an IPC program?  **Choose one answer**  **Please ask for all the documents that support the answer.**  **Review the document(s) together with the respondents and answer questions 1A-C** | 1. No 2. Yes |  |
| 1a | Does the existing IPC program have clearly defined responsibilities and annual work plan?  **SELECT ALL THAT APPLY** | 1. No 2. The existing program has clearly defined responsibilities 3. The existing program has annual work plan |  |
| 1b | Does the existing IPC program have clearly defined IPC objectives for the facility? Choose one answer | 1. No 2. Yes, IPC objectives, but there is no evidence that they are based on local epidemiology and priorities according to risk assessments 3. Yes, IPC objectives based on local epidemiology and priorities according to risk assessments |  |
| 1c | Does the existing IPC program have clearly defined IPC measurable outcome indicators for the facility? Choose one answer | 1. No 2. Yes, IPC measurable outcome indicators (or adequate measures for improvement) 3. Yes, IPC, measurable outcome indicators and future targets |  |
| 2 | Does your facility have a budget specifically allocated to the IPC programme (e.g., to address IPC materials, administrative support, staff)?  **Choose one answer** | 1. No 2. Yes 3. Don’t know |  |
| 3 | Do you consider the budget allocated for IPC as sufficient to cover your needs?  **Choose one answer** | 1. No 2. Yes |  |
| 4 | Is there an IPC team with one or more IPC staff with dedicated time for IPC activities?  **Choose one answer** | 1. No 2. Yes | SKIP to 8 |
| 5 | How many staff (nurses and/or doctors and/or epidemiologists and/or others) working on the IPC team?  **Please ask for a copy of the Terms of Reference for the Team or the IPC Focal point and the document that certifies their appointment.** | ______epidemiologist  ______nurses  ______physicians  ______others |  |
| 6 | Does the IPC team have at least one full-time IPC specialist (a nurse and/or a doctor and/or others) working 100% on IPC?  **Choose one answer**  **Please verify the answer based on the available documents.** | 1. All IPC staff work part-time on IPC 2. 1 or more full-time IPC specialist for 250 beds and less 3. Less than 1 full-time IPC specialist for 250 beds and less |  |
| 7 | Have members of your IPC team received formal IPC course training?  **Choose one answer**  **Please ask for any documents that would verify the answer** | 1. None were trained 2. Some were trained 3. All were trained |  |
| 8 | What IPC professional development options for the IPC team are available at your facility?  **SELECT ALL THAT** | 1. No professional development offered 2. IPC members attend IPC-related conferences or workshops 3. IPC members attend IPC-related training courses |  |
| 9 | Do you have an IPC Committee or an equivalent actively supporting the IPC team?  **Choose one answer**  **Please ask for a copy of the document appointing members of an Infection Control Committee.** | 1. No 2. Yes | SKIP to 12 |
| 10 | Which, if any, of the following professional groups are represented or included in the IPC Committee or an equivalent?  **SELECT ALL THAT APPLY**  **Please verify the answer based on the available documents.** | 1. Facility management (e.g., administrative director, chief executive officer (CEO), medical director) 2. Senior clinical staff (e.g., chief physician, chief of nursing) 3. Other facility management [e.g., biosafety, waste, those tasked with addressing water, sanitation and health (WASH)] |  |
| 11 | Did the committee meet in the past 12 months?  **Choose one answer** | 1. No 2. Yes | SKIP to 13 |
| 12 | Did you keep notes for the IPC committee meetings conducted in the past 12 months?  **Choose one answer**  **If yes, please ask for a copy of meeting notes from all the meetings conducted during the past 12 months and verify the answer.** | 1. No 2. Yes, for some 3. Yes, for all |  |
| 13 | When was the last time someone from the facility management, led or participated in a meeting to discuss IPC-related objectives, targets and challenges?  **Choose one answer**  **Please verify the answer based on the available documents.** | 1. Never 2. More than 3 months ago 3. Within the past 3 months |  |
| 14 | Does your facility have microbiological laboratory support for routine day-to-day use?  **Choose one answer** | 1. No 2. No, but the facility has access to a clinical laboratory at another site 3. Yes, an on-site laboratory is available |  |
| **IPC Trainings**  **Next, I would like to discuss the IPC trainings available to your staff. We will cover trainings that are part of new employee orientation and continuous educational opportunities for existing staff, regardless of level and position, for example trainings for senior administration and housekeeping staff. Trainings can include classroom, e-learning, bedside, and simulation training. We will also discuss periodic evaluations conducted to determine the effectiveness of your facility’s training program and assess staff knowledge** | | | |
| 15 | Did this facility conduct any IPC trainings for clinical staff and others having contact with patients or wards during the past 12 months?  **SELECT ALL THAT APPLY**  **Please ask for copies of all training materials (agenda and list of participants) from all IPC-related trainings conducted during the past 12 months** | 1. No IPC trainings conducted at this facility 2. Yes, for health care workers (clinical staff) 3. Yes, for non-clinical staff with access to patients or wards at your facility (e.g., cleaners, auxiliary service staff, administrative and managerial staff) 4. Yes, for family members, other caregivers or visitors | SKIP to 23 SKIP to 23 if only 4 is marked |
| 16 | Does this facility keep track of which clinical and non-clinical staff have been trained in IPC?  **Choose one answer**  **Please verify the answer based on the available documents. If there is no documented proof that they track training participation, please mark** | 1. No 2. Yes |  |
| 17 | Did IPC trainings for clinical and non-clinical staff conducted during the past 12 months include interactive training sessions (simulations and/or bedside trainings)?  **Choose one answer**  **Please verify the answer based on the available documents.**  **If not verified, then mark a different answer** | 1. No, interactive trainings only included written information and/or oral instructions and/or e-learning 2. Some trainings also included interactive sessions 3. All trainings included interactive sessions |  |
| 18 | Who led IPC trainings for clinical and non-clinical staff conducted during the past 12 months?  **SELECT ALL THAT APPLY**  **Please verify the answer based on the available documents.** | 1. External trainers from outside the facility 2. IPC team members 3. Non-IPC personnel |  |
| 19 | How did you assess the effectiveness of IPC trainings conducted during the past 12 months?  **SELECT ALL THAT APPLY**  **Please verify the answer based on the available documents.** | 1. No assessment 2. Pre/post test 3. Post-training survey for participants 4. Compliance monitoring of IPC practices |  |
| 20 | In general, which statement best describes when IPC training for clinical staff at your facility is delivered?  **SELECT ALL THAT APPLY**  **Please verify the answer based on the available documents (training registers, tracking sheets, prikazes, etc).** | 1. All new HCWs are trained as part of new employee orientation 2. Ongoing regular training at least annually, but not mandatory 3. Ongoing mandatory training at least annually (not confirmed by any documentation) 4. Ongoing mandatory training at least annually (verified by documentation) 5. None of the above |  |
| 21 | In general, which statement best describes when IPC training for non-clinical staff in your facility is delivered?  **SELECT ALL THAT APPLY**  **Please verify this answer based on the available documents (training registers, tracking sheets, prikazes, etc).** | 1. All new HCWs are trained as part of new employee orientation 2. Ongoing regular training at least annually, but not mandatory 3. Ongoing mandatory training at least annually (not confirmed by any documents) 4. Ongoing mandatory training at least annually (verified by documentation) 5. None of the above |  |
| 22 | Do clinical trainings conducted at your facility for physicians working in specialty areas (for example, surgery or anesthesiology) include IPC?  **For example, if there is a line insertion training, would HH and skin prep standards be embedded in it, not just taught separately as IPC training?**  **Choose one answer**  **Please verify the answer based on the available documents.** | 1. No clinical trainings for specialists conducted at the facility 2. Clinical trainings for specialists are conducted, but IPC is not included 3. Yes, in some trainings 4. Yes, in all trainings |  |
| **IPC Monitoring and Audit**  **One role of the IPC team is to monitor or audit IPC practices and provide feedback to staff in order to improve the quality of care and practice. An example of this is conducting hand hygiene observations to monitor staff compliance with appropriate hand hygiene practices** | | | |
| 23 | Does this facility have an internal IPC monitoring/audit plan with any of the following?  SELECT ALL THAT APPLY  **Please verify the answer based on the available documents. Only select options that were verified by the document review.** | 1. No facility monitoring/audit plan available 2. Yes, with clear goals and objectives 3. Yes, with tools to collect data in a systematic way (for example checklists) 4. Yes, with clearly defined roles and responsibilities 5. Yes, with work plan or schedule |  |
| 24 | 24 When was the last time there was an internal IPC monitoring/audit to assess compliance of any IPC practices at your facility?  **Choose one answer** | 1. Within the past 3 months 2. Within the past 6 months 3. Within the past 12 months 4. More than 12 months ago or never | SKIP to 27 |
| 24a | Did IPC staff document in any form implementation of monitoring/ audits conducted within the past 12 months documented?  **Please ask for a copy of all the available IPC monitoring/audit reports conducted during the past 12 months** | 1. No 2. Yes | SKIP to 27 |
| 25 | During the past 12 months, how often did you conduct monitoring/auditing of different IPC practices listed below in questions 25A-J?  **Choose one answer for each of the following questions 25A-J Please verify the answers based on the available documents.** |  |  |
| 25a | Hand hygiene compliance  (Using any observation tools)  **If monitoring tools are not available (checklist and schedule), but facility staff claim to conduct monitoring frequently, mark “Periodically but no regular schedule”** | 1. Not conducted 2. Periodically but no regular schedule 3. Weekly 4. Monthly 5. Every 6 months 6. Once a year |  |
| 25b | Consumption/usage of alcohol-based hand rub or soap  **If monitoring tools are not available (checklist and schedule), but facility staff claim to conduct monitoring frequently, mark “Periodically but no regular schedule”** | 1. Not conducted 2. Periodically but no regular schedule 3. Weekly 4. Monthly 5. Every 6 months 6. Once a year |  |
| 25c | Injection safety  **If monitoring tools are not available (checklist and schedule), but facility staff claim to conduct monitoring frequently, mark “Periodically but no regular schedule”** | 1. Not conducted 2. Periodically but no regular schedule 3. Weekly 4. Monthly 5. Every 6 months 6. Once a year |  |
| 25d | Waste management  **If monitoring tools are not available (checklist and schedule), but facility staff claim to conduct monitoring frequently, mark “Periodically but no regular schedule”** | 1. Not conducted 2. Periodically but no regular schedule 3. Weekly 4. Monthly 5. Every 6 months 6. Once a year |  |
| 25e | Cleaning of the ward environment  **If monitoring tools are not available (checklist and schedule), but facility staff claim to conduct monitoring frequently, mark “Periodically but no regular schedule”** | 1. Not conducted 2. Periodically but no regular schedule 3. Weekly 4. Monthly 5. Every 6 months 6. Once a year |  |
| 25f | Disinfection and sterilization of medical equipment/instruments **If monitoring tools are not available (checklist and schedule), but facility staff claim to conduct monitoring frequently, mark “Periodically but no regular schedule”** | 1. Not conducted 2. Periodically but no regular schedule 3. Weekly 4. Monthly 5. Every 6 months 6. Once a year |  |
| 25g | Transmission-based precautions, isolation and cohorting (grouping) of patients to prevent the spread of multidrug resistant organisms (MDRO)  **If monitoring tools are not available (checklist and schedule), but facility staff claim to conduct monitoring frequently, mark “Periodically but no regular schedule”** | 1. Not conducted 2. Periodically but no regular schedule 3. Weekly 4. Monthly 5. Every 6 months 6. Once a year |  |
| 25h | Consumption/usage of antimicrobial agents  **If monitoring tools are not available (checklist and schedule), but facility staff claim to conduct monitoring frequently, mark “Periodically but no regular schedule”** | 1. Not conducted 2. Periodically but no regular schedule 3. Weekly 4. Monthly 5. Every 6 months 6. Once a year |  |
| 25i | Intravascular catheter insertion and/or care  **If monitoring tools are not available (checklist and schedule), but facility staff claim to conduct monitoring frequently, mark “Periodically but no regular schedule”** | 1. Not conducted 2. Periodically but no regular schedule 3. Weekly 4. Monthly 5. Every 6 months 6. Once a year |  |
| 25j | Wound dressing change  **If monitoring tools are not available (checklist and schedule), but facility staff claim to conduct monitoring frequently, mark “Periodically but no regular schedule”** | 1. Not conducted 2. Periodically but no regular schedule 3. Weekly 4. Monthly 5. Every 6 months 6. Once a year |  |
| 26 | During the past 12 months, how did you share results of these internal monitoring /auditing of IPC practices with the following facility staff listed in questions 26A-E?  **SELECT ALL THAT APPLY** | | |
| 26a | Clinical staff? | 1. Did not share 2. Shared orally during staff meetings 3. Shared in a form of a written report |  |
| 26b | Non-clinical staff that have direct contact with patients? | 1. Did not share 2. Shared orally during staff meetings 3. Shared in a form of a written report |  |
| 26c | Clinical managers/heads of department? | 1. Did not share 2. Shared orally during staff meetings 3. Shared in a form of a written report |  |
| 26d | IPC committee? | 1. Did not share 2. Shared orally during staff meetings 3. Shared in a form of a written report |  |
| 26e | Non-clinical management (CEO, administration, board)? | 1. Did not share 2. Shared orally during staff meetings 3. Shared in a form of a written report |  |
| 27 | During the past 12 months, how often were your monitoring results used to make unit/facility- specific plans for the improvement of IPC practices?  **Choose one answer**  **If yes, please ask to provide examples. Please verify the answers based on the examples provided.** | 1. Always 2. Sometimes 3. Never |  |
| 28 | Do you assess IPC safety cultural factors in your facility? Show example of the surveys (Appendix 9)  **Choose one answer** | 1. No 2. Yes |  |
| **HAI Surveillance**  **HAI surveillance program describe the incidence and prevalence of HAIs in your facility, detect outbreaks in particular wards or patient populations, guide IPC strategies and priorities, and assess the impact and effectiveness of interventions.** | | | |
| 29 | Does this facility conduct HAI surveillance?  **Choose one answer**  **Please ask for all the available HAI surveillance guidelines or other documents.**  **Review the documents and answer question 29A.** | 1. No 2. Yes | SKIP to 38 |
| 29a | Does HAI surveillance include any of the following?  **Please verify the answers based on the available documents. SELECT ALL THAT APPLY** | 1. List of priority healthcare associated infections which are major causes of morbidity and mortality in the facility   **If prioritization process is not described in the document, please ask to describe the process used to identify infections which are major causes of morbidity and mortality in the facility**   1. Standardized case-definitions (defined numerator and denominator) 2. Standardized data collection methods 3. Processes to review data quality (for example, assessment of case report forms, review of microbiology results, denominator determination, etc.) 4. Clearly defined roles and responsibilities of staff involved in surveillance 5. Annual work plan and schedule 6. None of the above | SKIP to 38 |
| 30 | Are you conducting HAI surveillance in your facility for the following infection types listed in questions 30A-G?  **Choose one answer**  **Please ask for all the available HAI surveillance reports.** | | |
| 30a | Infections or colonization caused by multidrug-resistant pathogens (non-susceptibility to at least one agent in three or more antimicrobial categories)? | 1. No 2. Yes |  |
| 30b | Device-associated infections (for example, catheter-associated urinary tract infections, central line-associated bloodstream infections, peripheral-line associated bloodstream infections, ventilator-associated pneumonia)? | 1. No 2. Yes |  |
| 30c | Surgical site infections? | 1. No 2. Yes |  |
| 30d | Infections that may affect health care workers (for example, hepatitis B or C, HIV, influenza)? | 1. No 2. Yes |  |
| 30e | Infections in targeted vulnerable patient populations (for example, neonates, intensive care unit, immunocompromised, burn patients)? | 1. No 2. Yes |  |
| 30f | Local priority epidemic-prone infections (for example, norovirus, influenza, tuberculosis)? | 1. No 2. Yes |  |
| 30g | Clinically-defined (based on symptoms) infections? | 1. No 2. Yes |  |
| 31 | What data sources do you use for your HAI surveillance?  **SELECT ALL THAT APPLY**  **Please verify the answers based on the available documents.** | 1. Discharge diagnosis data 2. Voluntary notification from physicians or nurses 3. Ward-based assessments (e.g., chart review, discussion with nurses or physicians, patient exam) 4. Laboratory-based assessment (e.g., review of blood cultures) 5. None of these types of surveillance |  |
| 32 | Have staff conducting HAI surveillance been trained in basic epidemiology, surveillance, and IPC (i.e., capacity to oversee surveillance methods and manage/analyze/interpret data)? | 1. No 2. Yes |  |
| 33 | During the past 12 months, how were your HAIs surveillance data shared with facility staff?  SELECT ALL THAT APPLY  Please ask for any available reports or staff meetings to verify the answers | 1. Not shared with facility staff 2. Written reports 3. Oral updates 4. Presentation |  |
| 34 | How often, do you provide up-to-date HAIs surveillance information to the following groups listed in questions 34A-E? **Choose one answer for questions 34A-E**  **Please verify the answers based on the available documents** |  |  |
| 34a | Clinical staff? | 1. Never 2. Quarterly 3. Half-yearly 4. Annually 5. Periodically but no regular schedule |  |
| 34b | Non-clinical staff that have direct contact with patients | 1. Never 2. Quarterly 3. Half-yearly 4. Annually 5. Periodically but no regular schedule |  |
| 34c | Clinical managers/heads of department? | 1. Never 2. Quarterly 3. Half-yearly 4. Annually 5. Periodically but no regular schedule |  |
| 34d | IPC committee? | 1. Never 2. Quarterly 3. Half-yearly 4. Annually 5. Periodically but no regular schedule |  |
| 34e | Non-clinical management? | 1. Never 2. Quarterly 3. Half-yearly 4. Annually 5. Periodically but no regular schedule |  |
| 35 | Are HAIs surveillance data used to make unit/facility- specific plans for the improvement of IPC practices?  **Choose one answer**  **Please ask to provide examples and verify the answer** | 1. No 2. Yes |  |
| 36 | What best describes the microbiology laboratory capacity available to support HAI surveillance in this facility?  **Choose one answer** | 1. Laboratory is able to differentiate between gram positive and gram-negative strains but cannot identify the pathogen 2. The laboratory can identify pathogens (e.g., isolate identification) 3. The laboratory can identify pathogens and antimicrobial susceptibility patterns |  |
| 37 | Do you use any informatics/IT tools to support your HAI surveillance (for example, electronic health records)? | 1. No 2. Yes |  |
| 38 | How often do you analyze and report antimicrobial drug resistance data?  **Choose one answer** | 1. Never or rarely 2. Regularly (e.g., quarterly/ half a year /annually) |  |
| **IPC Guidelines**  **Facility IPC guidelines provide recommendations for IPC practices in a facility and may be adapted from existing international and national standards. Guidelines are often broad and high-level while standard-operating-procedures are more detailed step-by-step instructions more specific to a certain setting** | | | |
| 39 | Does your facility have any IPC Guidelines?  **Please ask for all the IPC Guidelines available at the facility. Review the documents to verify the answer.** | 1. No 2. Yes | SKIP to 44 |
| 40 | Which statement best describes the process you use in this facility to develop or adapt IPC Guidelines?  **Choose one answer** | 1. Facility uses international guidelines that have not been adapted to facility context 2. Facility uses national guidelines 3. Facility develops its own guidelines | SKIP to 42 |
| 41 | Who participates in the development and/or adaptation of the facility level Guidelines?  **SELECT ALL THAT APPLY** | 1. IPC personnel 2. Senior facility leadership Clinical staff 3. Facility management (e.g., biosafety, waste, WASH (i.e those tasked with addressing water, sanitation and health) 4. Quality managers |  |
| 42 | Do the facility training materials reflect the most updated IPC Guidelines?  **Choose one answer** | 1. No training materials available 2. No 3. Yes 4. Don’t know |  |
| 43 | Please describe the process you use to train HCW on IPC guidelines when they are issued/updated?  **Choose one answer**  **Please ask to see any training notes and/or list of participants and agenda from the last training.** | 1. Trainings only included written information and/or oral instructions 2. Some trainings included interactive sessions 3. All trainings included interactive sessions |  |
| 44 | Does your facility have any IPC SOPs?  **Choose one answer**  **Please ask for a copy of all the SOPs available at the facility. Review the documents to verify the answer.** | 1. No 2. Yes, SOPs not adapted to this facility 3. Yes, adapted to this facility | SKIP to 46 |
| 45 | Who participates in the development and/or adaptation of the facility level SOPs?  **Choose one answer**  **SELECT ALL THAT APPLY** | 1. IPC personnel 2. Senior facility leadership 3. Clinical staff 4. Facility management (e.g. Biosafety, Waste, WASH (i.e those tasked with addressing water, sanitation and health) 5. Quality managers |  |
| **Multimodal strategies**  **The term multimodal strategy refers to the implementation of several elements or components in an integrated way with the aim of improving an outcome and changing behavior. This multimodal strategy includes components such as system change which is the availability of infrastructure and supplies to enable IPC practices; education and training of healthcare workers and other hospital staff; monitoring of infrastructure, practices, processes, outcomes, and providing data feedback; reminders in the workplace; and culture change within the facility. In other words, the strategy involves “building” the right system, “teaching” the right things, “checking” the right things, “selling” the right messages, and ultimately “living” IPC throughout the entire health system.** | | | |
| 46 | For hand hygiene (HH) improvement activities, does your facility have any or all of the following elements listed in questions 46A-E?  **SELECT ALL THAT APPLY for questions 46A-E** | | |
| 46a | System change | 1. Element not included in work activities 2. Interventions to ensure the necessary infrastructure and continuous 3. Availability of supplies 4. Interventions to ensure optimal use and accessibility and prevent human error   **Please ask to provide examples to verify the answer** |  |
| 46b | Education and training on hand hygiene practices | 1. Element not included in work activities 2. Written information and/or oral instruction and/or e-learning 3. Interactive training sessions (includes simulation and/ or bedside training) |  |
| 46c | Monitoring of HH compliance and feedback | 1. Element not included in work activities 2. Audits of hand hygiene conducted 3. Audit results shared and discussed with health care workers and key players |  |
| 46d | Communication and reminders | 1. Element not included in work activities 2. Reminders, posters, or other tools used to promote or raise awareness of hand hygiene 3. Additional methods/initiatives to improve team communication across units and specialties (for example, multidisciplinary rounds?) |  |
| 46e | Safety climate and culture change | 1. Element not included in work activities 2. Managers/leaders (i.e. head of the hospital, chief clinicians, head of nursing) show visible support and act as champions and role models, promoting an adaptive approach and strengthening a culture that supports hand hygiene 3. Facility staff (clinical and non-clinical) are empowered to participate in hand hygiene improvement activities |  |
| 47 | How frequently is the WHO Hand Hygiene Self-Assessment Framework Survey conducted?  **Choose one answer**  **Show the Survey to remind people what it is.**  **Please verify the answers based on the available documents** | 1. Never 2. Periodically but not annually or on a regular schedule 3. At least annually |  |
| 48 | As far as your injection safety improvement activities, does your facility have any or all of the following elements listed in questions 48A-E? **SELECT ALL THAT APPLY for questions 48A-E** | | |
| 48a | System change | 1. Element not included in work activities 2. Interventions to ensure the necessary infrastructure and continuous availability of supplies 3. Interventions to ensure optimize use and accessibility and prevent human error   **Please ask to provide examples to verify the answer** |  |
| 48b | Education and training on hand hygiene practices | 1. Element not included in work activities 2. Written information and/or oral instruction and/or e-learning 3. Interactive training sessions (includes simulation and/ or bedside training) |  |
| 48c | Monitoring of injection safety compliance and feedback GIVE THE Injection Safety CHECKLIST as an example (Appendix 8) | 1. Element not included in work activities 2. Audits of injection safety conducted 3. Results shared and discussed with health care workers and key players |  |
| 48d | Communication and reminders | 1. Element not included in work activities 2. Reminders, posters, or other tools used to promote or raise awareness of injection safety 3. Additional methods/initiatives to improve team communication across units and disciplines (for example by facilitating multidisciplinary rounds?) |  |
| 48e | Safety climate and culture change | 1. Element not included in work activities 2. Managers/leaders (i.e. head of the hospital, chief clinicians, head of nursing) show visible support and act as champions and role models, promoting an adaptive approach and strengthening a culture that supports injection safety 3. Facility staff (clinical and non-clinical) are empowered to participate in injection safety improvement activities |  |
| 49 | Are your quality improvement staff involved in IPC activities? **Choose one answer** | 1. No quality improvement unit/staff at the facility 2. Quality improvement unit/staff available, but not involved in IPC 3. Quality improvement unit/staff available and involved in IPC |  |
| **IPC Infrastructure, Staffing, Workload and Supplies**  **Finally, I would like to ask a few questions about the facility’s infrastructure, and availability of staff and IPC supplies.** | | | |
| 50 | Are water services available at all times and of sufficient quantity for all uses (e.g., hand washing, drinking, personal hygiene, medical activities, sterilization, decontamination, cleaning and laundry)?  **Choose one answer** | 1. No, available on average < 5 days per week 2. Yes, available on average ≥ 5 days per week or every day but not of sufficient quantity 3. Yes, every day and of sufficient quantity |  |
| 51 | Is a reliable safe drinking water station present and accessible for staff, patients and families at all times and in all locations/wards?  **Choose one answer** | 1. No, not available 2. Sometimes, or only available in some places or not available for all users 3. Yes, accessible at all times and for all wards/groups |  |
| 52 | Is bed occupancy in your facility kept to one patient per bed? **Choose one answer** | 1. No 2. Yes, but not in all departments 3. Yes, for all units including pediatrics/neonatal and emergency |  |
| 53 | Do you place patients in beds outside of the room (in the corridor)  **Choose one answer** | 1. Never 2. Sometimes, or only in some departments |  |
| 54 | Do you ensure adequate spacing of >1 meter between patient beds?  **Choose one answer** | 1. No 2. Yes, but not in all departments 3. Yes, for all units including pediatrics and emergency |  |
| 55 | Do you have a responsible person/party to assess and respond when adequate bed capacity is exceeded?  **Choose one answer** | 1. No 2. Yes, the clinical head of department is responsible 3. Yes, the hospital administration/management is responsible |  |
| 56 | Are functioning hand hygiene stations (e.g., alcohol-based hand rub solution or soap and water with a basin/pan and clean single-use towels) available at all points of care?  **Choose one answer** | 1. No 2. Yes, stations present, but supplies are not always available 3. Yes, always available |  |
| 57 | In your facility, are ≥ 4 toilets or improved latrines (clean and functional) available for outpatient settings or ≥ 1 per 20 users for inpatient settings?  **Choose one answer** | 1. Less than the required number of latrines available and functioning 2. Sufficient number present but not all functioning or insufficient 3. Sufficient number present and functioning (4 or more (outpatients) and one per 20 users (inpatients)) |  |
| 58 | In your health care facility, is sufficient energy/power supply available at day and night for all uses (for example, pumping and boiling water, sterilization and decontamination, incineration or alternative treatment technologies, electronic medical devices, general lighting of areas where health care procedures are performed to ensure safe provision of health care and lighting of toilet hospitals and showers)?  **Choose one answer** | 1. No, never available 2. Yes, sometimes or only in some of the mentioned areas 3. Yes, always and in all mentioned areas |  |
| 59 | Is functioning environmental ventilation available in-patient care areas, including natural (using natural forces to vent air through windows/ doors) or mechanical ventilation?  **Choose one answer** | 1. No 2. Yes, in some patient care services 3. Yes, in all patient care areas |  |
| 60 | For floors and horizontal work surfaces, is there a visible record of cleaning, signed by the cleaners each day?  **Choose one answer** | 1. No record of floors and surfaces being cleaned 2. Record exists, but is not completed daily or is outdated 3. Yes, record completed daily |  |
| 61 | Are appropriate and well-maintained materials for cleaning (for example, detergent, mops, buckets, etc.) available?  **Choose one answer** | 1. No materials available 2. Yes, available but not well maintained (not labeled, broken, or dirty, etc.) 3. Yes, available and well-maintained |  |
| 62 | Do you have single patient rooms or rooms for cohorting (grouping based on common illness) patients with similar pathogens if the number of isolation rooms is insufficient or unavailable (for example, TB, measles, cholera)?  **SELECT ALL THAT APPLY** | 1. No single rooms and no rooms for cohorting (grouping) patients 2. Rooms suitable for patient cohorting available 3. Single rooms are available |  |
| 63 | Do you have functional waste collection containers for non-infectious (general) waste, infectious waste and, sharps waste at all waste generation points?  **Choose one answer** | 1. No bins or separate sharps disposal 2. Separate bins present but lids missing or more than 3/4 full; or two bins (instead of three); or bins at some but not all waste generation points. 3. Yes, all three containers |  |
| 64 | Is a functional burial pit/fenced waste dump or municipal pick-up available for disposal of non-infectious (non-hazardous/general waste)?  **Choose one answer** | 1. No pit or other disposal method used 2. Pit in facility but insufficient dimensions; pits/dumps overfilled or not fenced/locked; or irregular municipal waste pick up 3. Yes |  |
| 65 | Is outsourced waste disposal or an incinerator or alternative treatment for infectious and sharp waste (for example, an autoclave) functional and of a sufficient capacity?  **Choose one answer** | 1. No, none present 2. Yes |  |
| 66 | Are at least two pairs of household cleaning gloves and one pair of overalls or apron and boots in a good state and available for each cleaning and waste disposal staff member?  **Choose one answer** | 1. No, not available 2. Yes, available but in poor condition 3. Yes, in good condition |  |
| 67 | Is wastewater safely managed using on-site treatment (for example, septic tank followed by drainage pit) or sent to a functioning sewer system?  **Choose one answer** | 1. No, not present 2. Yes, available but in poor condition 3. Yes, in good condition |  |
| 68 | Does your health care facility provide a dedicated decontamination area and/or sterile supply department for the decontamination and sterilization of medical devices and other items/equipment?  **Choose one answer** | 1. No, not present 2. Yes, present, but not functioning 3. Yes |  |
| 69 | Do you reliably have sterile and disinfected equipment ready for use?  **Choose one answer** | 1. Available on average < 5 days per week 2. Available on average ≥ 5 days per week or every day, but not of sufficient quantity 3. Available every day and of sufficient quantity |  |
| 70 | Are disposable items available when necessary? (For example, injection safety devices (such as sharps injury protection syringes and reuse prevention syringes), examination gloves) **Choose one answer** | 1. Not available 2. Only sometimes available 3. Continuously available |  |

**PART 2**

**The checklist should be completed by the assessment team based on direct observations during a random walk around the facility. It is recommended that the random walk will include visits to one ICU (if available) and a typical ward (department). In case of hospitals that have pediatric and adult wards, the team needs to visit one adult ward and one pediatric ward. In each ward, the team must check a minor procedures room and 2–5 patient rooms.**

**Please check the availability of the following**

| **No** | **Question** | **Answer** | **Skip** |
| --- | --- | --- | --- |
| 73 | Visible reminders, posters, or other tools to promote or raise awareness of hand hygiene **Choose one answer** | 1. Not available 2. Available at some hand hygiene stations observed 3. Available at all hand hygiene stations observed |  |
| 74 | Visible reminders, posters, or other tools to promote or raise awareness of injection safety  **Choose one answer** | 1. Not available 2. Available at some units/wards/departments observed 3. Available at all units/wards/departments observed |  |
| 75 | Any patients in beds outside of the room (in the corridor) | 1. No 2. Yes |  |
| 76 | More than 1 meter between patient beds? | 1. No 2. Yes, but not in all departments 3. Yes, for all units including pediatrics and emergency |  |
| 77 | Hand hygiene stations (e.g., alcohol-based hand rub solution or soap and water with a basin/pan and clean single-use towels) available at all points of care?  Choose one answer | 1. Not available 2. Yes, stations present, but supplies are not always available 3. Yes, always available |  |
| 78 | Functional waste collection containers for non-infectious (general) waste, infectious waste and, sharps waste at all waste generation points? | 1. No bins or separate sharps disposal 2. Separate bins present but lids missing or more than 3/4 full; or two bins (instead of three); or bins at some but not all waste generation points. 3. Yes, all three containers |  |
| 79 | For floors and horizontal work surfaces, a visible record of cleaning, signed by the cleaners each day | 1. No record of floors and surfaces being cleaned 2. Record exists, but is not completed daily or is outdated 3. Yes, record completed daily |  |

**Translated version (Bahasa Indonesia)**

Target responden :

Nama responden :

Jabatan :

Nama pewawancara:

Nama petugas entry:

**BAGIAN 1**

| **No** | **Pertanyaan** | **Jawaban** | **Lewati** | **Dokumen** |
| --- | --- | --- | --- | --- |
| N1 | Kapan SK terakhir PPI ditanda tangani oleh direktur rumah sakit? |  |  |  |
| N2 | Kapan Ketua Komite/PPI diangkat sesuai dengan SK terakhir? |  |  |  |
| N3 | Kapan IPCN diangkat sesuai dengan SK terakhir? |  |  |  |
| N4 | Apakah IPCN sudah mengikuti pelatihan dasar PPI | - - - 1. Belum       2. Sudah, sebutkan tahunnya |  |  |
| N5 | Apakah IPCN sudah mengikuti pelatihan lanjutan PPI? | 1. Belum  2. Sudah, sebutkan tahunnya |  |  |
| N6 | Kapan IPCN diangkat sesuai dengan SK terakhir? |  |  |  |
| N7 | Apakah IPCN sudah mengikuti pelatihan dasar PPI | 1. Belum  2. Sudah, sebutkan tahunnya |  |  |
| N8 | Apakah IPCN sudah mengikuti pelatihan lanjutan PPI? | 1. Belum  2. Sudah, sebutkan tahunnya |  |  |
| **Komponen Utama 3: Pelatihan PPI**  **Selanjutnya, saya ingin mendiskusikan pelatihan PPI yang tersedia untuk staf Anda. Hal ini mencakup pelatihan yang merupakan bagian dari orientasi karyawan baru dan peluang pendidikan berkelanjutan untuk staf yang ada, terlepas dari level dan posisi, misalnya pelatihan untuk staf administrasi senior dan petugas kebersihan. Pelatihan dapat mencakup pelatihan di ruang kelas, e-learning, di samping tempat tidur, dan simulasi. Kami juga akan membahas evaluasi berkala yang dilakukan untuk menentukan keefektifan program pelatihan rumah sakit Anda dan menilai pengetahuan staf** | | | | |
| P1 | Apakah rumah sakit ini mengadakan pelatihan PPI untuk tenaga kesehatan (dokter, perawat, dll) dan personil lain (administrasi, petugas kebersihan, dll) yang berhubungan dengan pasien atau ruangan selama 12 bulan terakhir?  **PILIH SEMUA YANG SESUAI DENGAN KEADAAN RS**  **Silakan meminta salinan semua materi pelatihan (agenda dan daftar peserta) dari semua pelatihan terkait PPI yang dilakukan selama 12 bulan terakhir** | 1. Tidak ada pelatihan PPI yang dilakukan di rumah sakit ini 2. Ya, untuk petugas perawatan kesehatan (staf klinis) 3. Ya, untuk staf non-klinis yang memiliki akses ke pasien atau bangsal di rumah sakit Anda (misalnya petugas kebersihan, staf layanan tambahan, staf administrasi dan manajerial) 4. Ya, untuk keluarga anggota, pemberi perawatan lain atau pengunjung |  | Agenda pelatihan PPI di RS beserta daftar hadir peserta |
| P2 | Apakah rumah sakit ini mengidentifikasi **tenaga kesehatan** (dokter, perawat, dll) dan **personil lain (administrasi, petugas kebersihan**, dll) yang telah dilatih di PPI?  **Pilih satu jawaban**  **Mohon verifikasi jawaban berdasarkan dokumen yang tersedia. Jika tidak ada bukti terdokumentasi bahwa mereka melacak partisipasi pelatihan, harap tandai** | 1. Tidak 2. Ya |  | List/daftar pegawai yang sudah mengikuti pelatihan PPI |
| P3 | Apakah pelatihan PPI tenaga kesehatan (dokter, perawat, dll) dan personil lain (administrasi, petugas kebersihan, dll) yang dilakukan selama 12 bulan terakhir mencakup sesi pelatihan interaktif (simulasi dan/atau bed-side training (praktek langsung di lapangan) )?  **Pilih satu jawaban**  **Mohon verifikasi jawaban berdasarkan dokumen yang tersedia.**  **Jika belum diverifikasi, maka tandai jawaban yang berbeda** | 1. Tidak, pelatihan interaktif hanya menyertakan informasi tertulis dan / atau instruksi lisan dan / atau e-learning 2. Beberapa pelatihan juga menyertakan sesi interaktif 3. Semua pelatihan termasuk sesi interaktif |  | TOR Pelatihan PPI, Jadwal Pelatihan,dan Modul pelatihan PPI |
| P4 | Siapa yang memimpin pelatihan PPI untuk tenaga kesehatan (dokter, perawat, dll) dan personil lain (administrasi, petugas kebersihan, dll) yang dilakukan selama 12 bulan terakhir?  **PILIH SEMUA YANG SESUAI DENGAN KEADAAN RS**  **Harap verifikasi jawaban berdasarkan dokumen yang tersedia.** | 1. Pelatih eksternal dari luar rumah sakit 2. Anggota tim 3. Personil non-PPI |  | TOR Pelatihan PPI, Jadwal Pelatihan, Modul pelatihan PPI |
| P5 | Bagaimana Anda menilai efektivitas pelatihan PPI yang dilakukan selama 12 bulan terakhir?  **PILIH SEMUA YANG SESUAI DENGAN KEADAAN RS**  **Harap verifikasi jawaban berdasarkan dokumen yang tersedia.** | 1. Tidak ada penilaian 2. Tes pra / pasca 3. Survei pasca pelatihan untuk peserta 4. Pemantauan kepatuhan terhadap praktik PPI |  | Bukti audit kepatuhan kebersihan tangan |
| P6 | Secara umum, pernyataan mana yang paling menggambarkan saat pelatihan PPI untuk tenaga kesehatan di rumah sakit Anda diberikan?  **PILIH SEMUA YANG SESUAI DENGAN KEADAAN RS**  **Harap verifikasi jawaban berdasarkan dokumen yang tersedia (register pelatihan, lembar pelacakan, prikaze, dll).** | 1. Semua petugas kesehatan baru dilatih sebagai bagian dari orientasi karyawan baru 2. Pelatihan reguler berkelanjutan setidaknya setiap tahun, tetapi tidak wajib 3. Pelatihan wajib berkelanjutan setidaknya setiap tahun (tidak dikonfirmasi oleh dokumentasi apa pun) 4. Pelatihan wajib berkelanjutan setidaknya setiap tahun (diverifikasi dengan dokumentasi) 5. Tidak satu pun di atas |  | TOR Pelatihan PPI, Jadwal Pelatihan, Modul pelatihan, PPI Daftar hadir peserta, foto atau laporan kegiatan |
| P7 | Secara umum, pernyataan mana yang paling tepat menggambarkan kapan pelatihan PPI untuk personil lain (administrasi, petugas kebersihan, dll) di rumah sakit Anda diberikan?  **PILIH SEMUA YANG SESUAI DENGAN KEADAAN RS**  **Harap verifikasi jawaban ini berdasarkan dokumen yang tersedia (register pelatihan, lembar pelacakan, prikaze, dll).** | 1. Semua petugas kesehatan baru dilatih sebagai bagian dari orientasi karyawan baru 2. Pelatihan reguler berkelanjutan setidaknya setiap tahun, tetapi tidak wajib 3. Pelatihan wajib berkelanjutan setidaknya setiap tahun (tidak dikonfirmasi oleh dokumen apa pun) 4. Pelatihan wajib berkelanjutan setidaknya setiap tahun (diverifikasi dengan dokumentasi) 5. Tidak satu pun dari pilihan di atas |  | Daftar hadir peserta, foto atau laporan kegiatan |
| P8 | Apakah dilakukan pelatihan klinis di rumah sakit Anda untuk dokter yang bekerja di bidang khusus/spesialisasi (misalnya, pembedahan atau anestesiologi) dan menyertakan PPI?  **Misalnya, jika ada pelatihan *line insertion*, apakah kebersihan tangan / *hand hygiene* dan standar *skin prep* akan tertanam di dalamnya, tidak hanya diajarkan secara terpisah sebagai pelatihan PPI?**  **Pilih satu jawaban**  **Mohon verifikasi jawaban berdasarkan dokumen yang tersedia.** | 1. Tidak ada pelatihan klinis yang dilakukan di rumah sakit untuk spesialis 2. Pelatihan klinis untuk spesialis dilakukan, tetapi PPI tidak disertakan 3. Ya, di beberapa pelatihan 4. Ya, di semua pelatihan |  | Daftar hadir peserta atau foto atau daftar hadir |
| P9 | Apakah ada pelatihan PPI spesifik untuk pasien atau anggota keluarga yang bertujuan untuk meminimalisir potensi infeksi yang berkaitan dengan kesehatan (misalnya, pasien dengan imunosupresi, pasien dengan perangkat invasif, pasien dengan infeksi resistensi obat ganda / multidrug resistant)? | - - - 1. Ya       2. Tidak |  | Program PPI |
| P10 | Sebutkan 3 buah tantangan utama dalam pelaksanaan pelatihan PPI di RS anda |  |  |  |
| **Komponen utama 4: Surveilans HAI**  **Program surveilans HAI menjelaskan kegiatan dan prevalensi HAI di rumah sakit Anda, mendeteksi wabah di bangsal atau populasi pasien tertentu, memandu strategi dan prioritas PPI, dan menilai dampak dan efektivitas intervensi.** | | | | |
| P11 | Menurut pengertian anda, apa yang dimaksud dengan surveilans HAIs? |  |  |  |
| P12 | Apakah rumah sakit ini melakukan surveilans/pengawasan HAI?  **Pilih satu jawaban**  **Silakan tanyakan semua pedoman pengawasan HAI yang tersedia atau dokumen lainnya.** | 1. Tidak 2. Ya, siapakah penanggung jawabnya? | Jika jawaban tidak, LEWATI ke P68 | Program PPI, Pedoman Kerj PI, Dokumen surveilans HAIs, laporan surveilans |
| P13 | Bagaimana alur surveilans di rumah sakit anda, jelaskan secara singkat dari diagnosa sampai pelapora |  |  |  |
| P14 | Siapakah yang menjadi penentu untuk mendiagnosa HAIs di rumah sakit anda? | - - - 1. DPJP       2. IPCN       3. IPCD       4. Komite/tim PPI |  |  |
| P15 | Menurut anda, siapa saja yang sebaiknya terlibat dalam penentuan diagnosis HAIs? | - - - 1. DPJP       2. IPCN       3. IPCD       4. Komite/tim PPI |  |  |
| P16 | Apakah ada perbedaan persepsi antara DPJP dan IPCN dalam mendiagnosa HAIs? | 1. Tidak  2. Ada, cukup sering terjadi  3. Ada, tetapi jarang terjadi |  |  |
| P17 | Tindakan apa yang dilakukan untuk mengatasi perbedaan persepsi diagnosa HAIs antara DPJP dan IPCN? |  |  |  |
| P18 | Apakah surveilans HAI mencakup salah satu dari hal berikut ini?  **Silakan verifikasi jawaban berdasarkan dokumen yang tersedia.**  **PILIH SEMUA YANG SESUAI DENGAN KEADAAN RS** | - - - - 1. Daftar prioritas *healthcare associated infections* yang merupakan penyebab utama morbiditas dan mortalitas di rumah sakit. ICRA (Infection Control Risk Assessment) HAIs   **Jika proses pembuatan prioritas tidak dijelaskan dalam dokumen, harap tanyakan responden untuk menjelaskan proses yang digunakan dalam mengidentifikasi infeksi penyebab utama morbiditas dan mortalitas di rumah sakit**   - 1. Definisi kasus yang terstandarisasi (pembilang dan penyebut yang ditentukan)   2. Metode pengumpulan data yang terstandarisasi   3. Proses untuk meninjau kualitas data (misalnya, penilaian formulir laporan kasus, peninjauan hasil mikrobiologi, penentuan penyebut, dll.)   4. Peran dan tanggung jawab yang ditentukan dengan jelas dari staf yang terlibat dalam surveilans   5. Rencana dan jadwal kerja tahunan   6. Tidak satu pun dari pilihan diatas |  | Dokumen surveilans HAIs, laporan surveilans dan ICRA HAI |
| P19 | Apakah Anda melakukan surveilans HAI di rumah sakit Anda untuk Infeksi atau kolonisasi yang disebabkan oleh patogen yang resistan terhadap beberapa obat (tidak sensitif terhadap setidaknya satu agen dalam tiga atau lebih kategori antimikroba)? | 1. Tidak 2. Ya |  | Laporan surveilans HAIs |
| P20 | Apakah Anda melakukan surveilans HAI di rumah sakit Anda untuk Infeksi terkait alat (device associated infections) dibawah ini:  Boleh pilih lebih dari satu jawaban | 1. CAUTI (catheter-associated urinary tract infections), lanjutkan ke pertanyaan 2. PLABSI (peripheral-line associated bloodstream infections), lanjutkan ke pertanyaan 3. CLABSI (central line-associated bloodstream infections), lanjutkan ke pertanyaan 4. VAP (ventilator-associated pneumonia) |  | Laporan surveilans HAIs |
| P21 | Apakah kriteria yang digunakan untuk mendiagnosis CAUTI? |  |  |  |
| P22 | Guideline apa yang digunakan sebagai dasar untuk diganosa CAUTI? |  |  |  |
| P23 | Apakah ada petugas yang bertanggung jawab untuk memonitor penggunaan kateter urin pada pasien? | 1. Tidak 2. Ya, sebutkan |  |  |
| P24 | Apakah kriteria yang digunakan untuk mendiagnosis CLABSI? |  |  |  |
| P25 | Guideline apa yang digunakan sebagai dasar untuk diganosa CLABSI? |  |  |  |
| P26 | Apakah ada petugas yang bertanggung jawab untuk memonitor penggunaan kateter sentral pada pasien? | - - - 1. Tidak       2. Ya, sebutkan |  |  |
| P27 | Apakah kriteria yang digunakan untuk mendiagnosis IDO? |  |  |  |
| P28 | Guideline apa yang digunakan sebagai dasar untuk indakan IDO? |  |  |  |
| P29 | Apakah surveilans IDO dilakukan untuk seluruh tindakan operasi? | 1. Ya, lanjutkan ke pertanyaan P30  2. Tidak, lanjutkan ke pertanyaan selanjutnya |  |  |
|  | Sebutkan target surveilans IDO |  |  |  |
| P30 | Apakah dilakukan monitoring terhadap setiap pasien paska operasi? | 1. Ya, lanjutkan ke pertanyaan P31  2. Tidak, lanjutkan ke pertanyaan selanjutnya |  |  |
|  | Sebutkan monitoring paska operasi apa yang dilakukan |  |  |  |
| P31 | Apakah Anda melakukan surveilans HAI di rumah sakit Anda untuk Infeksi daerah operasi? | 1. Tidak 2. Ya |  | Laporan surveilans HAIs |
| P32 | Apakah Anda melakukan surveilans HAI di rumah sakit Anda untuk Infeksi yang dapat menyerang petugas kesehatan (misalnya, hepatitis B atau C, HIV, influenza)? | 1. Tidak 2. Ya, sebutkan |  | Laporan surveilans HAIs |
| P33 | Apakah Anda melakukan surveilans HAI di rumah sakit Anda untuk Infeksi pada populasi pasien rentan (misalnya, neonatus, unit perawatan intensif, gangguan kekebalan, pasien luka bakar)? | 1. Tidak 2. Ya, sebutkan |  | Laporan surveilans HAIs |
| P34 | Pada Rumah Sakit Anda, apakah surveilans dilakukan untuk penyakit infeksi terbanyak yang menjadi prioritas di daerah Anda (misalnya, difteri, tuberkulosis [TB], Multidrug-Resistant Tuberculosis [MDR-TB], thypoid, Covid-19)? | 1. Tidak 2. Ya, sebutkan |  | Laporan surveilans HAIs |
| P35 | Pada saat melakukan surveilans HAIs di rumah sakit anda, apakah diagnosis HAIs hanya berdasarkan pada tanda atau gejala klinis tanpa adanya pengujian mikrobiologis? | 1. Tidak 2. Ya, jelaskan kriteria gejala klinisnya |  | Laporan surveilans HAIs |
| P36 | Apakah Rumah Sakit Anda mengecek kualitas data secara teratur?  Apabila ya, sumber data apa yang Anda gunakan untuk surveilans HAI Anda?  **PILIH SEMUA YANG SESUAI DENGAN KEADAAN RS**  **Harap verifikasi jawaban berdasarkan dokumen yang tersedia.** | - - - 1. Tidak       2. Ya, penilaian indakan kasus       3. Ya, Tinjauan hasil mikrobiologi       4. Ya, Penentuan penyebut       5. Ya, Data penegakan diagnosis  1. Ya, Pemberitahuan sukarela dari dokter atau perawat 2. Ya, Penilaian berbasis lingkungan (misalnya, tinjauan grafik, diskusi dengan perawat atau dokter, pemeriksaan pasien) 3. Ya, Penilaian berbasis laboratorium (misalnya, tinjauan kultur darah) |  | Kamus indakan  Hasil kultur |
| P37 | Sudahkan staf anda yang bertanggung jawab untuk melakukan surveilans HAI dilatih tentang epidemiologi dasar, surveilans dan PPI (contohnya: kapasitas untuk mengawasi metode surveilans dan mengelola / menganalisis / menginterprestasi data)? | 1. Tidak 2. Ya |  | Sertifikat pelatihan surveilans HAIs |
| P38 | Selama 12 bulan terakhir, bagaimana data surveilans HAI Anda disosialisasikan/diinfokan kepada staf rumah sakit?  **PILIH SEMUA YANG SESUAI DENGAN KEADAAN RS**  Silakan meminta laporan atau rapat staf yang tersedia untuk memverifikasi jawaban | 1. Tidak dibagikan dengan staf rumah sakit 2. Laporan tertulis 3. Pembaruan lisan 4. Presentasi |  | Laporan HAIs, tanda terima laporan HAIs, bukti email penyampaian laporan atau daftar hadir sosialisasi surveilans HAIs/pertemuan rutin, rapat rutin komite PPI, performance board di unit masing2 |
| P39 | Seberapa sering, Anda memberikan informasi terkait surveilans/pengawasan HAI terkini kepada Tenaga indakan? | 1. Tidak pernah 2. Triwulanan 3. Setengah tahunan 4. Tiap tahun 5. Berkala tetapi tidak ada jadwal rutin |  | Bukti pelatihan, daftar hadir peserta |
| P40 | Seberapa sering, Anda memberikan informasi terkait surveilans/pengawasan HAI terkini kepada Komite medik/kepala instalasi/kepalaunit | 1. Tidak pernah 2. Triwulanan 3. Setengah tahunan 4. Tiap tahun 5. Berkala tetapi tidak ada jadwal rutin |  | Bukti pelatihan, daftar hadir peserta |
| P41 | Seberapa sering, Anda memberikan informasi terkait surveilans/pengawasan HAI terkini kepada Komite/tim PPI? | 1. Tidak pernah 2. Triwulanan 3. Setengah tahunan 4. Tiap tahun 5. Berkala tetapi tidak ada jadwal rutin |  | Bukti pelatihan, daftar hadir peserta |
| P42 | Seberapa sering, Anda memberikan informasi terkait surveilans/pengawasan HAI terkini kepada Manajemen rumah sakit? | 1. Tidak pernah 2. Triwulanan 3. Setengah tahunan 4. Tiap tahun 5. Berkala tetapi tidak ada jadwal rutin |  | Bukti pelatihan, daftar hadir peserta |
| P43 | Apakah data surveilans/pengawasan HAI digunakan untuk membuat rencana perbaikan pelaksanaan PPI di rumah sakit anda?  **Pilih satu jawaban**  **Silakan tanyakan untuk memberikan contoh dan verifikasi jawabannya** | 1. Tidak 2. Ya |  |  |
| P44 | Apa yang paling menggambarkan kapasitas laboratorium mikrobiologi yang tersedia untuk mendukung surveilans HAI di rumah sakit ini?  **Pilih satu jawaban** | 1. Laboratorium dapat membedakan antara strain gram positif dan gram negatif tetapi tidak dapat mengidentifikasi patogen 2. Laboratorium dapat mengidentifikasi patogen (misalnya identifikasi isolat) 3. Laboratorium dapat mengidentifikasi patogen dan pola kerentanan antimikroba |  |  |
| P45 | Apakah Anda menggunakan IT/indak informasi online untuk mendukung pengawasan HAI Anda (misalnya, catatan kesehatan elektronik)? | 1. Tidak 2. Ya |  |  |
| P46 | Bagaimana cara Rumah Sakit Anda memberikan umpan balik terhadap hasil surveilans HAIs? (setidaknya setahun sekali) | - - - 1. Tidak pernah       2. Dengan lisan/tertulis       3. Dengan presentasi/diskusi |  |  |
| P47 | Apakah Rumah Sakit Anda mengevaluasi secara berkala surveilans Rumah Sakit Anda agar sejalan dengan kebutuhan saat ini dan prioritas di Rumah Sakit Anda? | - - - 1. Ya  1. Tidak |  |  |
| P48 | Seberapa sering Anda menganalisis dan melaporkan data resistensi obat antimikroba?  **Pilih satu jawaban** | - - - 1. Tidak pernah atau jarang       2. Secara teratur (misalnya triwulanan / setengah tahunan / tahunan) |  | Laporan AMR/antibiogram |
| P49 | Apakah staf komite mutu dan keselamatan pasien Anda terlibat dalam kegiatan PPI? **Pilih satu jawaban** | 1. Tidak ada unit peningkatan kualitas / staf di rumah sakit 2. Unit/staf peningkatan kualitas tersedia, tetapi tidak terlibat dalam PPI 3. Unit/staf peningkatan Kualitas tersedia dan terlibat dalam PPI |  |  |
|  | Menurut Anda, apa tiga tantangan utama dalam melakukan surveilans HAIs di rumah sakit ini? |  |  |  |
| **Komponen utama 5: Strategi multimodal** | | | | |
| P50 | Sebelum mengikuti kajian ini, apakah anda sudah pernah mendengar atau mengetahui tentang strategi multimodal? | - - - 1. Tidak, lanjutkan ke pertanyaan P53       2. Ya, lanjutkan ke pertanyaan berikutnya |  |  |
| P51 | Apakah anda mengetahui apa yang dimaksud dengan multi modal strategi? | - - - 1. Tidak, lanjutkan ke pertanyaan P53       2. Ya, lanjutkan ke pertanyaan berikutnya |  |  |
| P52 | Menurut pendapat anda, apakah yang dimaksud dengan strategi multimodal?  Setelah RS menjawab, kita jelaskan tentang strategi multi modal  Penggunaan strategi multimodal di PPI telah terbukti sebagai pendekatan berbasis terbaik untuk mendapatkan indak yang berkelanjutan dan perubahan perilaku dalam implementasi indaka/intervensi PPI.  Strategi multimodal adalah implementasi ≥3 komponen secara terintegrasi untuk mencapai perbaikan dengan tujuan perubahan perilaku (misalnya, praktik kebersihan tangan).  Komponen-komponen tersebut meliputi: (i) perubahan sistem (misalnya, menyediakan infrastruktur, persediaan, dan sumber daya manusia yang diperlukan), (ii) indakan dan pelatihan bagi tenaga indakan dan personal lainnya (misalnya, manajer), (iii) monitoring dan umpan balik; (iv) komunikasi dan pengingat; dan (v) budaya keselamatan dan perubahan perilaku.  Alat monitoring, seperti checklist dan indak yang dikembangkan oleh tim yang terdiri dari multidisiplin ilmu dengan mempertimbangkan kondisi yang ada di rumah sakit. Kelima komponen tersebut harus dipertimbangkan dalam mengambil keputusan selain mempertimbangkan situasi di rumah sakit dan juga hasil dari surveilans yang dilakukan secara berkala. |  |  |  |
| P53 | Apakah RS anda menerapkan multi modal strategi dalam intervensi PPI? | - - - 1. Tidak       2. Ya |  |  |
| P54 | Apakah strategi modal berupa perubahan system dilakukan dalam upaya peningkatan kebersihan tangandi rumah sakit anda?  **Mohon berikan contoh untuk memverifikasi jawaban**  Sistem pemasangan handrub: di setiap tempat tidur, lift, dll  Sistem pengadaan handrub: Pembuatan handrub sendiri sesuai dengan standar WHO  Memasang poster-poster | 1. Tidak termasuk dalam strategi multimodal 2. Perubahan sistem dengan adanya intervensi untuk memastikan infrastruktur yang diperlukan dan ketersediaan pasokan yang berkesinambunganKetersediaan pasokan 3. Perubahan sistem dengan intervensi untuk memastikan infrastruktur yang diperlukan dan ketersediaan pasokan yang berkesinambungan, dekat dan mudah dijangkau, misalnya penempatan handrub di setiap tempat tidur |  |  |
| P55 | Apakah strategi multimodal di Rumah Sakit Anda meliputi Pendidikan dan Pelatihan (termasuk pelatihan online) tentang praktik kebersihan tangan? | 1. Tidak termasuk dalam strategi multimodal 2. Informasi tertulis dan/atau instruksi lisan dan / atau e-learning 3. Sesi pelatihan interaktif (termasuk simulasi dan / atau pelatihan di samping tempat tidur) |  |  |
| P56 | Apakah strategi multimodal di rumah sakit anda meliputi pemantauan kepatuhan kebersihan tangan dan umpan baliknya? | 1. Tidak termasuk dalam strategi multimodal 2. Audit kebersihan tangan dilakukan 3. Hasil audit dibagikan dan didiskusikan dengan petugas kesehatan dan pemain inti |  |  |
| P57 | Apakah strategi multimodal di rumah sakit anda meliputi komunikasi dan reminder tentang kebersihan tangan? | 1. Tidak termasuk dalam strategi multimodal 2. Pengingat, poster, atau alat lain yang digunakan untuk mempromosikan atau meningkatkan kesadaran akan kebersihan tangan 3. Metode / inisiatif tambahan untuk meningkatkan komunikasi tim lintas unit dan lintas spesialisasi (Contoh: komunikasi multidisiplin atau *multidisciplinary rounds*?) |  |  |
| P58 | Apakah strategi multimodal di rumah sakit anda meliputi Perubahan iklim dan budaya keselamatan terkait dengan kebersihan tangan? | 1. Tidak termasuk dalam strategi multimodal 2. Manajer / pemimpin (yaitu kepala rumah sakit, kepala dokter, kepala keperawatan) menunjukkan dukungan yang nyata dan bertindak sebagai juara dan teladan, mempromosikan adaptasi pendekatan dan penguatan budaya yang mendukung kebersihan tangan 3. Staf Rumah sakit (klinis dan non-klinis) diberdayakan untuk berpartisipasi dalam kegiatan peningkatan kebersihan tangan |  |  |
| P59 | Seberapa sering Survei Hand Hygiene Self-Assessment Framework (alat penilaian mandiri untuk kebersihan tangan) dari WHO dilakukan?  **Pilih satu jawaban**  **Tunjukkan Survei agar orang mendapat gambaran tentang survey tersebut.**  **Harap verifikasi jawaban berdasarkan dokumen yang tersedia** | 1. Tidak pernah 2. Secara berkala tetapi tidak setiap tahun atau dengan jadwal reguler 3. Setidaknya setiap tahun |  | Tools survey dan laporannya |
| P60 | Apakah strategi multimodal di rumah sakit anda meliputi perubahan sistem untuk peningkatan keselamatan injeksi  Sistem peningkatan keamanan pemberian injeksi  Penggunaan jarum untuk satu pasien untuk satu tindakan untuk satu pemberian  Obat-obat diusahakan single dose, maksimal penyimpanan obat 1x24 jam disimpan dalam kulkas dengan suhu yang terpantau  Dispensing obat  Sistem keamanan  Safety box | 1. Elemen tidak termasuk dalam kegiatan kerja 2. Intervensi untuk memastikan infrastruktur yang diperlukan dan ketersediaan pasokan yang berkelanjutan 3. Intervensi untuk memastikan penggunaan dan aksesibilitas yang optimal dan mencegah kesalahan manusia   **Mohon berikan contoh untuk memverifikasi jawaban** |  |  |
| P61 | Apakah strategi multimodal di rumah sakit anda meliputi Pendidikan dan pelatihan tentang peningkatan keselamatan injeksi | 1. Elemen tidak termasuk dalam kegiatan kerja 2. Informasi tertulis dan/atau instruksi lisan dan / atau e-learning 3. Sesi pelatihan interaktif (termasuk simulasi dan / atau pelatihan di samping tempat tidur) |  |  |
| P63 | Apakah strategi multimodal di rumah sakit anda meliputi Pemantauan kepatuhan dan umpan balik keselamatan injeksi  Berikan laporan audit keselamatan injeksi sebagai contoh (contoh jumlah nakes yang pernah tertusuk jarum) | 1. Elemen tidak termasuk dalam kegiatan kerja 2. Audit keselamatan injeksi dilakukan 3. Hasil dibagikan dan didiskusikan dengan petugas kesehatan dan pemain inti |  | Output surveilans |
| P64 | Apakah strategi multimodal di rumah sakit anda meliputi Komunikasi dan pengingat untuk peningkatan keselamatan injeksi | 1. Elemen tidak termasuk dalam aktivitas kerja 2. Pengingat, poster, atau alat lain yang digunakan untuk mempromosikan atau tingkatkan kesadaran tentang keamanan injeksi. 3. Tambahan saya gagasan / inisiatif untuk meningkatkan komunikasi tim lintas unit dan disiplin ilmu (misalnya dengan memrumah sakiti komunikasi multidisiplin atau *multidisciplinary round)* |  |  |
| P65 | Apakah strategi multimodal di rumah sakit anda meliputi Perubahan iklim dan budaya keselamatan untuk peningkatan keselamatan injeksi | 1. Elemen tidak termasuk dalam kegiatan kerja 2. Manajer/pemimpin (yaitu kepala rumah sakit, kepala dokter, kepala keperawatan) menunjukkan dukungan nyata dan bertindak sebagai juara dan panutan, mempromosikan pendekatan adaptif dan memperkuat budaya yang mendukung keselamatan injeksi 3. Staf Rumah sakit (klinis dan non-klinis) diberdayakan untuk berpartisipasi dalam kegiatan peningkatan keselamatan injeksi |  |  |
| P66 | Apakah penerapan strategi multimodal PPI melibatkan tim multidisiplin? | 1. Tidak  2. Ya |  |  |
| P67 | Apakah Anda secara teratur berkoordinasi dengan sejawat dari mutu dan keselamatan pasien untuk membangun dan meningkatkan strategi multimodal PPI? | 1. Tidak  2. Ya |  |  |
| P68 | Apakah strategi ini mencakup bundles atau daftar tilik/checklist?  Bundel adalah sekumpulan intervensi atau tindakan yang secara shahih dapat menurunkan kejadian HAIs | 1. Tidak  2. Ya |  |  |
| P69 | Menurut Anda, apa tiga tantangan utama dalam menerapkan strategi multimodal di rumah sakit ini? |  |  |  |
| **LAINNYA** | | | | |
| P70 | Apakah PMK No 27 tahun 2017 tentang PPI didiseminasikan kepada rumah sakit anda? | - - - 1. Ya, lanjutkan ke pertanyaan P72       2. Tidak, lanjut ke pertanyaan selanjutnya |  |  |
| P71 | Darimana anda mengetahui tentang PMK No.27 tahun 2017 tentang PPI? |  |  |  |
| P72 | Apakah ada topik yang perlu ditambahkan dalam PMK No. 27 tahun 2017 tentang PPI? | 1. Ya, lanjut ke pertanyaan selanjutnya  2. Tidak |  |  |
| P73 | Sebutkan topik yang perlu ditambahkan dalam PMK No. 27 tahun 2017 tentang PPI? |  |  |  |
|  | Apa kendala yang anda hadapi dalam pelaksanaan penggunaan APD yang rasional? |  |  |  |
|  | Apakah menurut anda juknis APD yang dikeluarkan oleh Kemenkes sudah jelas? | . Tidak  2. Ya |  |  |
|  | Sebutkan topik yang perlu ditambahkan dalam juknis APD yang dikeluarkan oleh Kemenkes? |  |  |  |

**PENGAMATAN**

**Daftar periksa harus dilengkapi oleh tim kajian berdasarkan pengamatan langsung (melalui video/video call) di sekitar rumah sakit. Direkomendasikan bahwa pengamatan (melalui video/video call) yang dilakukan ke satu ICU (jika tersedia) dan rawat biasa (departemen) untuk dewasa dan anak. Apabila tidak memungkinkan rumah sakit dapat mem-videokan keadaan di ICU dan ruang rawat inap biasa (anak dan dewasa) sebelum wawancara dilakukan. Di setiap ruangan, tim harus memeriksa ruang prosedur minor dan 2–5 ruang pasien.**

**Silakan periksa ketersediaan berikut**

| **No** |  | **Pertanyaan** | **Answer** | **Dokumen** |
| --- | --- | --- | --- | --- |
| P74 | P92 | Terlihat pengingat, poster, atau alat lain yang terlihat untuk mempromosikan atau meningkatkan kesadaran akan kebersihan tangan  **Pilih satu jawaban** | 1. Tidak tersedia 2. Tersedia di beberapa stasiun kebersihan tangan yang diamati 3. Tersedia di semua stasiun kebersihan tangan yang diamati | Video 1 atau video call |
| P75 | P92 | Terlihat pengingat, poster, atau alat lain untuk mempromosikan atau meningkatkan kesadaran akan keamanan injeksi  **Pilih satu jawaban** | 1. Tidak tersedia 2. Tersedia di beberapa unit / bangsal / departemen yang diamati 3. Tersedia di semua unit / bangsal / departemen yang diamati |  |
| P76 | D76 | Ada pasien di tempat tidur di luar ruangan (di koridor) | 1. Tidak 2. Ya | Video 2 atau video call |
| P77 | D77 | Jarak lebih dari 1 meter di antara tempat tidur pasien? | 1. Tidak 2. Ya, tetapi tidak di semua departemen 3. Ya, untuk semua unit termasuk pediatri dan darurat | Video 3 atau video call |
| P78 | D87 | Untuk lantai dan permukaan kerja horizontal, terlihat catatan pembersihan, ditandatangani oleh pembersih setiap hari | 1. Tidak ada catatan lantai dan permukaan yang dibersihkan 2. Catatan ada, tetapi tidak diselesaikan setiap hari atau sudah usang 3. Ya, catatan diselesaikan setiap hari | Video 6 atau video call |

**Additional file 7. Flow of the study**

**Additional file 8. Category of IPCAF score**

| **IPCAF Score** | **Category** | **Interpretation** |
| --- | --- | --- |
| 0–200 | Inadequate | IPC core components implementation is deficient. Significant improvement is required |
| 201–400 | Basic | Some aspects of the IPC core components are in place, but not sufficiently implemented. Further improvement is required |
| 401–600 | Intermediate | Most aspects of the IPC core components are appropriately implemented. The facility should continue to improve the scope, implementation, and quality and focus on the development of long-term plans to sustain and promote the existing IPC program activities |
| 601–800 | Advanced | The IPC core components are fully implemented according to the WHO recommendations and appropriate to the facility’s needs |

**Additional file 9. Total IPCAF score**


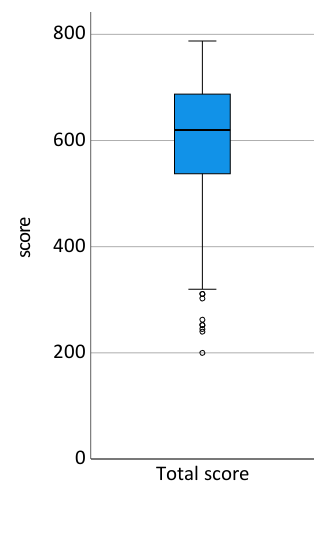


**Additional file 10. Core components with a score more than 90%**

| **Core component (CC)** | **Answer** | | | | **N (%)** |
| --- | --- | --- | --- | --- | --- |
| **CC1. IPC program** | | | | | |
| IPC team included both doctors and nurse | Yes | | | | 347 (97.7) |
| The IPC committee actively supporting the IPC team | Yes | | | | 327 (92.1) |
| Representation of professional groups in the IPC committee |  | | | |  |
| Facility management (e.g. biosafety, waste, and those tasked with addressing water, sanitation, and hygiene [WASH]) | Yes | | | | 325 (91.5) |
| **CC2. IPC guidelines** | | | | | |
| Guideline | Standard precautions | | | | 347 (97.7) |
|  | Hand hygiene | | | | 352 (99.2) |
|  | Transmission-based precautions | | | | 335 (94.4) |
|  | Prevention of surgical site infection (SSI) | | | | 336 (94.6) |
|  | Prevention of CAUTI* | | | | 338 (95.2) |
|  | Disinfection and sterilization | | | | 352 (99.2) |
|  | Health care worker protection and safety | | | | 331 (93.2) |
|  | Injection safety | | | | 345 (97.2) |
|  | Waste management | | | | 352 (99.2) |
| Guidelines available in facilities consistent with national/international guidelines | Yes | | | | 352 (99.2) |
| Guidelines available in facilities adapted according to the local needs and resources while maintaining key IPC standards | Yes | | | | 339 (95.5) |
| **CC4. Surveillance HAIs** | | | | | |
| **Organization of surveillance** |  | | | |  |
| Surveillance is a defined component of the IPC program | Yes | | | | 349 (98.3) |
| Availability of personnel that responsible for surveillance activities | Yes | | | | 339 (95.5) |
| **Conducted surveillance for:** |  | | | |  |
| Surgical Site Infection (SSI) | Yes | | | | 333 (93.8) |
| Device-associated infections (for example, CAUTI*, CLABSI**, PLABSI***, VAP****) | Yes | | | | 329 (92.7) |
| **Methods of surveillance** |  | | | |  |
| Availability of reliable surveillance case definitions (defined numerator and denominator according to international definitions [e.g. CDC NHSN/ECDC] or if adapted, through an evidence-based adaptation process and expert consultation | Yes | | | | 336 (94.6) |
| Surveillance data is used to make tailored unit/facility-based plans for the improvement of IPC practices | Yes | | | | 334 (94.1) |
| **CC6. Monitoring/audit of IPC practices and feedback** | | | | | |
| Indicators and processes monitored in the facility | |  |  | | |
| Hand hygiene compliance (using the WHO hand hygiene observation tool or equivalent) | | Yes | 354 (99.7) | | |
| Cleaning of the ward environment | | Yes | 333 (93.8) | | |
| Disinfection and sterilization of medical equipment/instruments | | Yes | 323 (91.0) | | |
| Waste management | | Yes | 335 (94.4) | | |
| **Availability of feedback auditing reports (for example, feedback on hand hygiene compliance data or other processes) on the state of the IPC activities/performance** | | | | | |
| The reporting of monitoring data undertaken regularly (at least annually) | | Yes | | 327 (92.1) | |
| Monitoring and feedback of IPC processes and indicators performed in a “blame-free” institutional culture aimed at improvement and behavioural change | | Yes | | 339 (95.5) | |
| **CC7. Workload, staffing and bed occupancy** | | | | | |
| **Bed occupancy** | |  | |  | |
| Patients in the facility placed in beds standing in the corridor outside of the room (including beds in the emergency department) | | No | | 324 (91.2) | |
| **CC8. Built environment, materials, and equipment for IPC at the facility level** | | | | | |
| **Water** | |  | |  | |
| Water services available at all times and of sufficient quantity for all uses (for example, hand washing, drinking, personal hygiene, medical activities, sterilization, decontamination, cleaning and laundry) | | Yes, every day and of sufficient quantity | | 330 (93.0) | |
| **Power supply, ventilation and cleaning** | |  | |  | |
| Sufficient energy/power supply available at day and night for all uses (for example, pumping and boiling water, sterilization and decontamination, incineration or alternative treatment technologies, electronic medical devices, general lighting of areas where health care procedures are performed to ensure safe provision of health care and lighting of toilet facilities and showers) | | Yes, always and in all mentioned areas | | 348 (98.0) | |
| Functioning environmental ventilation (natural or mechanical) available in patient care areas | | Yes | | 329 (92.7) | |
| **Medical waste management and sewage** | |  | |  | |
| Functional burial pit/fenced waste dump or municipal pick-up available for disposal of non-infectious (non-hazardous/general waste) | | Yes | | 331 (93.3) | |
| Wastewater treatment system (for example, septic tank followed by drainage pit) present (either on or off site) and functioning reliably | | Yes and functioning reliably | | 322 (90.7) | |
| **Decontamination and sterilization** | |  | |  | |
| Disposable items available when necessary? (for example, injection safety devices, examination gloves) | | Yes, continuously available | | 344 (96.9) | |

**Additional file 11. Core components with a score less than 50%**

| **Core component (CC)** | **Answer** | **N (%)** |
| --- | --- | --- |
| **CC2. IPC guidelines** | | |
| Availability of expertise (in IPC and/or infectious disease) for developing or adapting guidelines | Yes | 135 (38.0) |
| **CC3. Education and training** | | |
| Availability of personnel with IPC expertise (in IPC and/or infectious disease) to lead IPC training | Yes | 161 (45.4) |
| IPC training integrated in the clinical practice and training of other specialties (for example, training of surgeons involves aspects of IPC) | No | 180 (50.7) |
|  | Yes, in some disciplines | 115 (32.4) |
|  | Yes, in all disciplines | 60 (16.9) |
| Availability of specific IPC training for patients or family members to minimize the potential for HAIs (e.g., immunosuppressed patients, patients with invasive devices, patients with MDR infections) | Yes | 162 (45.6) |
| **CC4. Surveillance HAIs** | | |
| Organization of surveillance |  |  |
| Colonization or infections caused by multidrug-resistant pathogens according to your local epidemiological situation | Yes | 128 (35.8) |
| Infections in vulnerable populations (for example, neonates, ICU, immunocompromised, burn patients) | Yes | 168 (47.3) |
| Infections that may affect health care workers in clinical, laboratory, or other settings (e.g. hepatitis B or C, HIV, influenza) | Yes | 174 (48.9) |
| Methods of surveillance: availability of adequate microbiology and laboratory capacity to support surveillance | No | 203 (57.2) |
|  | Yes, can differentiate gram-positive/negative strains but cannot identify pathogens | 54 (15.2) |
|  | Yes, can reliably identify pathogens (e.g. isolation identification) in a timely manner | 29 (8.2) |
|  | Yes, can reliably identify pathogens and antimicrobial drug | 69 (19.4) |
| Information analysis and dissemination/data use, linkage, and governance: analysed antimicrobial drug resistance on a regular basis (for example, quarterly/half-yearly/annually | Yes | 119 (33.5) |
| **CC6. Monitoring/audit of IPC practices and feedback** | | |
| Indicators and processes monitored in the facility: consumption/usage of antimicrobial agents | Yes | 110 (31.0) |

**Additional file 12. Characteristics of the interviewed hospitals and interviewees (N = 38 hospitals)**

| **Characteristics of hospitals (38 hospitals)** | **Number (%)** |
| --- | --- |
| Class:  A  B  C  D | 3 (7.9)  9 (23.7)  13 (34.2)  13 (34.2) |
| Region  1 (Java, Sumatera, Bali)  2 (Sulawesi, West Nusa Tenggara, and Kalimantan)  3 (East Nusa Tenggara, Maluku, Papua, and West Papua) | 19 (50)  11 (28.9)  8 (21.1) |
| Ownership of the hospitals  Government  Private  State-owned enterprises | 22 (57.9)  14 (36.8)  9 (5.3) |
| Interviewees (management of the hospital)  Director  Director as well as the head of the committee  Senior management (medical director, head of medical division, head of nurse division)  Management staff | 10 (26.3)  1 (2.6)  26 (68.4)  1 (2.6) |
| Interviewees (IPC team/committee)  Head of ICP committee/team  IPCN  Members of IPC committee  Head of ICP committee and IPCN  Head of ICP committee and members of IPC committee  IPCN and members of the committee | 4 (10.5)  15 (39.5)  2 (5.3)  9 (23.7)  4 (10.5)  4 (10.5) |

**Additional file 13. Interviews with the management of the hospitals (N = 38 hospitals)**

| **Question** | **Answer** | **N (%)** |
| --- | --- | --- |
| The last time managements joined/participated in the IPC meeting to discuss about objectives, targets and challenges in implementing IPC in the hospital. | More than 3 months ago | 15 (39.5) |
|  | Never | 2 (5.3) |
|  | Within the past 3 months | 21 (55.3) |
| Management allocated specific budget to IPC programmes (to address IPC materials, administrative support, staff) | Yes | 23 (60.5) |
| The budget allocated for IPC sufficient to cover the IPC programmes? | No | 23 (60.5) |
| The IPC committee or team communicate the report of monitoring and surveillance with the management | No | 4 (10.5) |
|  | Yes, only written report without explanation | 15 (39.5) |
|  | Yes, with explanation and we discuss this together on a meeting | 19 (50.0) |
| Actions done when the hospital has a high number of HAIs cases | Identification the problem and find the possible solution with IPC committee/team | 19 (50.0) |
|  | Re-education by conducting in house training for particular issue | 10 (26.3) |
|  | Collect sample and performed culture or re-cultured (if possible) | 3 (7.9) |
|  | Improving role of IPCLN in reporting and provide rewards for IPCLN | 2 (5.3) |
|  | Develop system for reporting HAIs and intensive coordination with IPC committee/team | 2 (5.3) |
|  | Add funding for training | 1 (2.6) |
|  | Improved facilities and infra structure | 1 (2.6) |
| How do you overcome a shortage of IPCN? | Enhance the coordination and activate IPCLN,IPCD and head of the ward to help IPCN and | 9 (23.7) |
|  | Motivated and train more nurses to be IPCN | 7 (18.4) |
|  | Add more IPCN and funding | 3 (7.9) |
|  | Includes position of IPCN in the structure | 2 (5.3) |

**Additional file 14. Interviews with the IPC committee/team of the hospitals (N = 38 hospitals)**

| **Position of the interviewees in the hospital** | **N (100%)** |
| --- | --- |
| Head of IPC committee | 4 (10.5) |
| IPCN | 15 (39.5) |
| Members of IPC committee | 2 (5.3) |
| Head of IPC committee and IPCN | 9 (23.7) |
| Head of IPC committee and members of IPC committee | 4 (10.5) |
| IPCN and members of the IPC committee | 4 (10.5) |

| **Core component (CC)** | **Answers** | **N (%)** |
| --- | --- | --- |
| **CC3. Education and training** |  |  |
| Availability of personnel with IPC expertise (in IPC and/or infectious disease) to lead IPC training | No | 11 (28.9) |
|  | Yes | 27 (71.1) |
| Availability of additional non-IPC personnel with adequate skills to serve as trainers and mentors (for example, link nurses or doctors, champions?) | No | 30 (78.9) |
|  | Yes | 8 (21.1) |
| Frequency of healthcare workers receive training regarding IPC in your facility | Never or rarely | 2 (5.3) |
|  | New employee orientation only for other personnel | 31 (81.6) |
|  | New employee orientation and regular (at least annually) training for other personnel offered but not mandatory | 12 (31.6) |
|  | New employee orientation and regular (at least annually) mandatory IPC training for all health care workers | 12 (31.6) |
| Frequency of cleaners and other personnel directly involved in patient care receive training regarding IPC in the facility | Never or rarely | 3 (7.9) |
|  | New employee orientation only for other personnel | 31 (81.6) |
|  | New employee orientation and regular (at least annually) training for other personnel offered but not mandatory | 8 (21.1) |
|  | New employee orientation and regular (at least annually) mandatory IPC training for all health care workers | 13 (34.2) |
| Administrative and managerial staff receive general training regarding IPC in the facility | No | 3 (7.9) |
|  | Yes | 35 (92.1) |
| How healthcare workers and other personnel trained | No training available | 12 (31.6) |
|  | Using written information and/or oral instruction and/or e-learning only | 12 (31.6) |
|  | Includes additional interactive training sessions (for example, simulation and/or bedside training) | 14 (36.8) |
| Periodic evaluations of the effectiveness of training programmes (for example, hand hygiene audits, other checks on knowledge) | No | 9(23.7) |
|  | Yes, but not regularly | 29 (76.3) |
|  | Yes, regularly (at least annual) |  |
| IPC training integrated in the clinical practice and training of other specialties (for example, training of surgeons involves aspects of IPC) | No | 18 (47.4) |
|  | Yes, in some disciplines | 13 (34.2) |
|  | Yes, in all disciplines | 3 (7.9) |
| Availability of specific IPC training for patients or family members to minimize the potential for health care-associated infections (for example, immunosuppressed patients, patients with invasive devices, patients with multidrug-resistant infections) | No | 8 (21.1) |
|  | Yes | 30 (78.9) |
| Ongoing development/education offered for IPC staff (for example, by regularly attending conferences, courses) | No |  |
|  | Yes |  |
| **CC4. Surveillance of HAIs** |  |  |
| **Organization of surveillance** |  |  |
| Surveillance is a defined component of the IPC programme | No | 5 (13.2) |
|  | Yes | 33 (86.8) |
| Availability of personnel that responsible for surveillance activities | No |  |
|  | Yes |  |
| The professionals responsible for surveillance activities been trained in basic epidemiology, surveillance and IPC (that is, capacity to oversee surveillance methods, data management and interpretation) | No | 12 (31.6) |
|  | Yes | 26 (68.4) |
| Availability of informatics/IT support to conduct your surveillance (for example, equipment, mobile technologies, electronic health records) | No | 28 (73.7) |
|  | Yes | 10 (26.3) |
| Conducted a prioritization exercise to determine the HAIs to be targeted for surveillance according to the local context (that is, identifying infections that are major causes of morbidity and mortality in the facility) | No | 19 (50) |
|  | Yes | 19 (50) |
| **Conducted surveillance for:** |  |  |
| Surgical site infections | No | 11 (28.9) |
|  | Yes | 27 (71.1) |
| Device-associated infections (for example, catheter-associated urinary tract infections, central line-associated bloodstream infections, peripheral-line associated bloodstream infections, ventilator-associated pneumonia) | No |  |
|  | Yes |  |
| Clinically-defined infections (for example, definitions based only on clinical signs or symptoms in the absence of microbiological testing) | No | 11 (28.9) |
|  | Yes | 27 (71.1) |
| Colonization or infections caused by multidrug-resistant pathogens according to your local epidemiological situation | No | 31 (81.6) |
|  | Yes | 7 (18.4) |
| Local priority epidemic-prone infections (for example, typhoid, tuberculosis [TB], COVID-19) | No | 11 (28.9) |
|  | Yes | 27 (71.1) |
| Infections in vulnerable populations (for example, neonates, intensive care unit, immunocompromised, burn patients)? | No | 18 (47.4) |
|  | Yes | 20 (52.6) |
| Infections that may affect health care workers in clinical, laboratory, or other settings (for example, hepatitis B or C, human immunodeficiency virus [HIV], influenza) | No | 18 (47.4) |
|  | Yes | 20 (52.6) |
| Conduct a regular evaluation to determine if the surveillance is in line with the current needs and priorities of the facility | No | 10 (26.3) |
|  | Yes | 28 (73.7) |
| **Methods of surveillance** |  |  |
| Availability of reliable surveillance case definitions (defined numerator and denominator according to international definitions [e.g. CDC NHSN/ECDC] or if adapted, through an evidence-based adaptation process and expert consultation | No | 14 (36.8) |
|  | Yes | 24 (63.2) |
| There is a standardized data collection methods (for example, active prospective surveillance) according to international surveillance protocols (for example, CDC NHSN/ECDC) or if adapted, through an evidence-based adaptation process and expert consultation | No | 15 (39.5) |
|  | Yes | 23 (60.5) |
| There is a processes in place to regularly review data quality (for example, assessment of case report forms, review of microbiology results, denominator determination, etc.) | No | 22 (57.9) |
|  | Yes | 16 (42.1) |
| Availability of adequate microbiology and laboratory capacity to support surveillance | No | 25 (65.8) |
|  | Yes, can differentiate gram-positive/negative strains but cannot identify pathogens | 3 (7.9) |
|  | Yes, can reliably identify pathogens (for example, isolate identification) in a timely manner | 1 (2.6) |
|  | Yes, can reliably identify pathogens and antimicrobial drug | 9 (23.7) |
| Surveillance data is used to make tailored unit/facility-based plans for the improvement of IPC practices | No | 7 (18.4) |
|  | Yes | 31 (81.6) |
| Analysed antimicrobial drug resistance on a regular basis (for example, quarterly/half-yearly/annually) | No | 27 (71.1) |
|  | Yes | 11 (28.9) |
| Frontline health care workers (doctors/nurses) | No | 8 (21.1) |
|  | Yes | 31 (78.9) |
| Clinical leaders/heads of department | No | 9 (23.7) |
|  | Yes | 29 (76.3) |
| IPC committee | No | 9 (23.7) |
|  | Yes | 29 (76.3) |
| Non-clinical management/administration (chief executive officer/chief financial officer) | No | 6 (15.8) |
|  | Yes | 32 (84.2) |
| How feedback up-to-date surveillance information (at least annually) is done | No feedback | 14 (36.8) |
|  | By written/oral information only | 11 (28.9) |
|  | By presentation and interactive problem-oriented solution finding | 13 (34.2) |
| **CC4. Multimodal Strategies** |  |  |
| Before this study, have you ever heard about multimodal strategy? | No | 30 (78.9) |
|  | Yes | 8 (21.1) |
| Do you know what multimodal strategy is | No | 31 (81.6) |
|  | Yes | 7 (18.4) |
| MMS used to implement IPC interventions | No | 7 (18.4) |
|  | Yes | 31 (81.6) |
| **MMS includes any or all the following elements** |  | Hand Hygiene |
| System change | Element not included in multimodal strategies | 6 (15.8) |
|  | Interventions to ensure the necessary infrastructure and continuous availability of supplies are in place | 22 (57.9) |
|  | Interventions to ensure the necessary infrastructure and continuous availability of supplies are in place and addressing ergonomics and accessibility, such as the best placement of central venous catheter set and tray | 24 (63.2) |
| Education and training | Element not included in multimodal strategies | 3 (7.9) |
|  | Written information and/or oral instruction and/or e-learning only | 28 (73.7) |
|  | Additional interactive training sessions (includes simulation and/or bedside training) | 31 (81.6) |
| Monitoring and feedback | Element not included in multimodal strategies | 5 (13.2) |
|  | Monitoring compliance with process or outcome indicators (for example, audits of hand hygiene or catheter practices) | 28 (73.7) |
|  | Monitoring compliance and providing timely feedback of monitoring results to health care workers and key players | 22 (57.9) |
| Communication and reminders | Element not included in multimodal strategies | 1 (2.6%) |
|  | Reminders, posters, or other advocacy/awareness-raising tools to promote the intervention | 36 (94.7) |
|  | Additional methods/initiatives to improve team communication across units and disciplines (for example, by establishing regular case conferences and feedback rounds) | 22 (57.9) |
| Safety climate and cultural change | Element not included in multimodal strategies | 1 (2.6) |
|  | Managers/leaders show visible support and act as champions and role models, promoting an adaptive approach and strengthening a culture that supports IPC, patient safety and quality | 31 (81.6) |
|  | Additionally, teams and individuals are empowered so that they perceive ownership of the intervention (for example, by participatory feedback rounds) | 32 (84.2) |
| Multidisciplinary team is used to implement IPC multimodal strategies | No | 10 (26.3) |
|  | Yes | 28 (73.7) |
| Regularly link to colleagues from quality improvement and patient safety to develop and promote IPC multimodal strategies | No | 13 (34.2) |
|  | Yes | 25 (65.8) |
| Strategies included bundles or checklists | No | 11 (28.9) |
|  | Yes | 27 (71.1) |
